# Targeted therapy in advanced *BRAF*-mutated colorectal cancer: systematic review and network meta-analysis

**DOI:** 10.1136/bmj-2025-086026

**Published:** 2025-11-19

**Authors:** Bao-Dong Qin, Xiao-Dong Jiao, Zhan Wang, Ke Liu, Yan Ling, Ying Wu, Yuan-Sheng Zang

**Affiliations:** Department of Medical Oncology, Changzheng Hospital, Naval Medical University, Shanghai 200003, China

## Abstract

**Objective:**

To investigate the individual and comparative efficacy and safety of targeted therapy based strategies in advanced *BRAF*-mutated colorectal cancer.

**Design:**

Systematic review and network meta-analysis.

**Data sources:**

PubMed, Embase, Cochrane Library, and ClinicalTrials.gov from inception to 31 May 2025, and international conference proceedings.

**Eligibility criteria for selecting studies:**

Clinical trials and multicentre, real world studies investigating the efficacy and safety of targeted therapies for advanced *BRAF*-mutated colorectal cancer with at least one of the clinical outcomes of interest.

**Data synthesis:**

The primary endpoint was overall survival in the first line and second or later line settings. Secondary endpoints included progression-free survival, objective response rate, disease control rate, and grade ≥3 adverse events. Single arm meta-analysis, pairwise meta-analysis, and network meta-analysis were performed to pool hazard ratios with 95% credible intervals (CrIs) for overall survival and progression-free survival, and odds ratios with 95% CrIs for objective response rate, disease control rate, and adverse events. The rankogram and surface under the cumulative ranking curve (SUCRA) evaluated the relative superiority of regimens in the network meta-analysis.

**Results:**

60 studies involving 4633 patients with advanced *BRAF*-mutated colorectal cancer were included. Pooled estimates indicated that patients could benefit from an anti-EGFR (epidermal growth factor receptor)/BRAF based regimen. Doublet chemotherapy (DCT)-anti-EGFR/BRAF was associated with the best overall survival, providing significant benefits compared with DCT-anti-VEGF (vascular endothelial growth factor) (hazard ratio 0.49, 95% CrI 0.36 to 0.66), triplet chemotherapy-anti-VEGF (0.51, 0.33 to 0.80), and anti-EGFR/BRAF (0.70, 0.51 to 0.96) regimens in the first line setting. Chemotherapy-anti-EGFR/BRAF showed significant superiority in overall survival (SUCRA=0.94 for DCT-anti-EGFR/BRAF, 0.90 for single agent chemotherapy (SCT)-anti-EGFR/BRAF) and progression-free survival (0.93 for DCT-anti-EGFR/BRAF and 0.92 for SCT-anti-EGFR/BRAF) among all first line targeted therapy strategies. In the second or later line setting, anti-EGFR/BRAF with or without an additional inhibitor (anti-MEK (mitogen-activated protein kinase kinase) or anti-PI3K (phosphoinositide 3-kinase)), exhibited better efficacy compared with other alternative strategies, ranking highest across study endpoints based on rank probability and SUCRA.

**Conclusions:**

For initial treatment of advanced *BRAF*-mutated colorectal cancer, combining doublet chemotherapy with anti-EGFR/BRAF therapy offers the best survival benefit. For patients who have had previous treatment, anti-EGFR/BRAF regimens (with or without a MEK inhibitor) are the most effective and tolerable options.

**Systematic review registration:**

PROSPERO CRD420250653959.

## Introduction

Mutations of the *BRAF* gene represent critical molecular alterations in colorectal cancer, with the *BRAF V600E* variant being the most frequently observed subtype.^1^ ^2^ Notably, *BRAF*-mutated colorectal cancers account for about 8-15% of all patients with colorectal cancer, establishing it as a clinically distinct subtype characterised by highly aggressive tumours, including poor prognosis and intrinsic resistance to conventional chemotherapy.^3^ ^4^ ^5^ This subgroup of patients presents substantial challenges for treatment in advanced disease settings, particularly due to the limited efficacy of current regimens and the urgent need for optimal biomarker driven therapeutic strategies. Currently, anti-VEGF agents in combination with chemotherapy are often recommended as first line treatment for advanced *BRAF*-mutated colorectal cancer.^6^ ^7^ However, some important controversies persist about key clinical decisions, including optimal intensity of chemotherapy (see box 1 for definitions of abbreviations)—doublet (eg, FOLFOX or FOLFIRI) versus triplet (FOLFOXIRI) chemotherapy, and the selection of targeted therapy agents (anti-VEGF versus anti-EGFR (epidermal growth factor receptor) agents.^8^ ^9^ ^10^ ^11^ ^12^ ^13^ These debates stem from insufficient evidence, particularly the lack of large scale studies on this molecularly defined population.

Box 1 **Definition of abbreviations**

*BRAF*—B-Raf proto-oncogene, serine/threonine kinase
*EGFR*—epidermal growth factor receptor
*ERK*—extracellular signal-regulated kinase
*FOLFIRI*—folinic acid, 5-fluorouracil, and irinotecan
*FOLFOX*—folinic acid, 5-fluorouracil, and oxaliplatin
*FOLFOXIRI*—folinic acid, 5-fluorouracil, oxaliplatin, and irinotecan
*MEK*—mitogen-activated protein kinase kinase
*PD-1*—programmed cell death protein-1
*PI3K*—phosphoinositide 3-kinase
*VEGF*—vascular endothelial growth factor
*WNT*—wingless/integrated

Owing to the limited efficacy of conventional therapeutic strategies for *BRAF*-mutated colorectal cancer, BRAF targeted therapies have been explored to improve therapeutic efficacy. BRAF inhibitor monotherapy has not yielded clinically significant improvements,^14^ ^15^ however, likely due to compensatory feedback reactivation of EGFR mediated mitogen-activated protein kinase signalling pathways, a well documented mechanism for resistance in preclinical models.^16^ ^17^ Subsequently, the finding of EGFR mediated feedback activation has driven the exploration of dual EGFR/BRAF blockade in *BRAF*-mutated colorectal cancer. Trials, including BEACON, have shown the efficacy of EGFR/BRAF co-inhibition in second or later line settings, whereas BREAKWATER and SWOG1406 further established the superiority of anti-EGFR/BRAF regimens combined with chemotherapy in both first line and later line treatments.^18^ ^19^ ^20^ Although targeted therapy has been shown to improve outcomes, heterogeneity in the design of clinical trials raises important questions about the optimal line of therapy and combination strategies, including the optimal combination regimen among available strategies targeting VEGF and EGFR signalling pathways; the sufficiency of targeted therapy alone versus its combination with chemotherapy, and, if combined, whether single agent or doublet chemotherapy provides superior efficacy; the survival benefit of first line versus later line initiation of targeted based therapy; and the comparative safety profiles of targeted therapy based regimens, specifically whether they show a manageable increase in toxicity while maintaining acceptable safety.

To clarify these key clinical uncertainties, we conducted a systematic review and meta-analysis with two primary objectives: to integrate pooled efficacy and safety data for individual regimens through conventional meta-analysis, overcoming the limitations of small-sample studies, and to perform direct and indirect comparisons of targeted therapies through pairwise and network meta-analyses, addressing the scarcity of head-to-head trials. We identified current optimal first line and later line therapeutic strategies for *BRAF*-mutated colorectal cancer while guiding future trial design by prioritising high value combination approaches based on comparative effectiveness and safety profiles.

## Methods

This systematic review, meta-analysis, and network meta-analysis was conducted using a predetermined protocol following the Preferred Reporting Items for Systematic Reviews and Meta-Analyses (PRISMA) extension statement for network meta-analyses (see supplementary table S1).^21^ The study protocol was registered in the Prospective Register of Systematic Reviews (PROSPERO).

### Search strategy

We performed a systematic literature search of PubMed, Embase, the Cochrane library, and ClinicalTrials.gov for eligible studies investigating the efficacy and safety of targeted therapy based strategies for advanced *BRAF*-mutated colorectal cancer (last updated on 31 May 2025). We also searched abstracts and presentations from international conferences, such as the American Society of Clinical Oncology, European Society for Medical Oncology, and American Association for Cancer Research. The reference lists of relevant articles were checked for additional studies. Supplementary table S2 provides the complete search details.

### Study selection and data extraction

To be included, studies had to meet the following criteria: phase 1, 2, and 3 clinical trials or multicentre, large, real world studies (published and unpublished); enrolled participants with histologically or cytologically confirmed advanced *BRAF*-mutated colorectal cancer; evaluated targeted therapy based regimens on at least one of the clinical outcomes of interest, including overall survival, progression-free survival, objective response rate, disease control rate, and grade ≥3 adverse events, or with sufficient data to enable calculation of these outcomes.^22^ ^23^ We excluded studies that included patients with advanced *BRAF*-mutated colorectal cancer but did not independently report relevant data for the population with the mutation, contained insufficient published data or original data for meta-analysis, and focused on populations with non-*BRAF V600E* mutations. If studies reported updated data multiple times, we included information from the most recent version. All treatment regimens were classified according to their drug class or mechanism of action (eg, doublet chemotherapy, triplet chemotherapy, EGFR inhibitor (anti-EGFR), VEGF inhibitor (anti-VEGF), BRAF inhibitor (anti-BRAF), MEK inhibitor (anti-MEK)) irrespective of the specific agent type. This classification approach was motivated by both the well established therapeutic equivalence among regimens within the same drug class, as shown by clinical evidence and treatment guideline, and the necessity to maintain analytical robustness by preventing network fragmentation and preserving statistical validity. Two reviewers (B-DQ and X-DJ) independently screened titles and abstracts and then evaluated the full texts of potentially eligible studies for final inclusion. A third reviewer (Y-SZ) resolved any disagreements.

Two reviewers (B-DQ and XD-J) independently extracted relevant data from each study using a prespecified form, including study characteristics (first author, publication year, country, study identification, trial registration number, study design), demographic information (sample size, data source), treatment (regimen, treatment line), and outcomes (hazard ratios for overall survival and progression-free survival, and the number of participants with objective response rate, disease control rate, and grade ≥3 adverse events). We applied several requirements during data extraction: although the study reported data on multiple *BRAF* mutation subtypes, we prioritised the analysis of *BRAF V600E* variants; we preferred blinded independent central review assessed data derived from the intention-to-treat population; we preferred treatment related grade ≥3 adverse events; we prioritised the latest updated data from repeated reports of individual trials with varying follow-up periods for inclusion; we deemed targeted therapy based regimens with fewer than 10 patients to be insufficient for statistical analysis; and we exclusively used the data from single arm studies for single arm meta-analyses of objective response rate, disease control rate, and grade ≥3 adverse events, whereas they were not incorporated into pairwise meta-analysis or network meta-analyses as these studies lacked comparator arms. During data extraction, study characteristics were categorised according to prespecified classifications, including data source (trial level data versus subgroup level data), *BRAF* mutation type (*BRAF V600E* versus mixed type), treatment history (uniform treatment versus mixed treatment), and publication type (published literature versus conference report).

### Risk assessment of bias across included studies

We evaluated the methodological quality of the included studies using validated assessment tools: the Cochrane Risk of Bias tool version 2 (RoB 2) for randomised controlled trials,^24^ the Methodological Index for Non-randomised Studies for single arm interventional studies and non-randomised controlled trials,^25^ the Assessment of Real-World Observational Studies framework and Risk Of Bias In Non-randomized Studies of Interventions for real world evidence investigations^26^ (see supplementary tables S3-S6). In addition, the Confidence in Network Meta-Analysis (CINeMA) framework was used to evaluate the confidence in effect estimates across six domains: within study bias, reporting bias, indirectness, imprecision, heterogeneity, and incoherence. Two reviewers (B-DQ and X-DJ) independently evaluated the methodological quality and risk of bias of the individual studies, with a third reviewer (Y-SZ) resolving any disagreements by consensus.

### Statistical analysis

The enrolled studies were stratified by treatment line: first line therapy versus second or later line therapy. The primary outcome analysed was overall survival, and the secondary outcomes analysed were progression-free survival, objective response rate, disease control rate, and grade ≥3 adverse events. Data analyses were performed in R (version 4.3.2) by implementing three core methods: single arm meta-analysis and pairwise meta-analyses using the meta package, and bayesian network meta-analysis using the gemtc package.

A single arm meta-analysis was performed to synthesise pooled estimates for objective response rate, disease control rate, and the occurrence of grade ≥3 adverse events for each regimen, based on all available arm level data from the included studies reporting quantifiable outcome measures. We restricted pairwise meta-analyses to direct comparisons between two or more controlled trials. Hazard ratios with 95% credible intervals (CrIs) were used to quantify time-to-event outcomes, including progression-free survival and overall survival, and odds ratios with 95% CrIs were used to assess binary endpoints, including objective response rate, disease control rate, and grade ≥3 adverse events. Heterogeneity across studies was evaluated using the χ^2^ statistic based on Cochran’s Q and was quantified using the inconsistency index (I^2^) test. When I^2^ was >50% or the P value based on Cochran’s Q was <0.10, we implemented random effects models. Otherwise, we used fixed effects models.

Network meta-analysis was performed in a bayesian framework using the Markov Chain Monte Carlo simulation technique, comparing any two targeted therapy based strategies by simultaneously synthesising direct and indirect evidence. We generated network plots for each outcome to illustrate the geometries and clarify which treatments were compared directly or indirectly across the included studies. A random effects consistency model was applied to allow for heterogeneity between studies in the treatment comparison effects. Markov Chain Monte Carlo sampling was performed with four parallel chains, each consisting of 20 000 iterations, after a 5000 iteration adaptation phase for algorithm tuning using JAGS.

We used trace plots, density plots, and the Brooks-Gelman-Rubin method^27^ to evaluate convergence of the modes (see supplementary figures S1 and S2). Within the bayesian framework, the network meta-analysis estimated the overall rankings of treatments by calculating the surface under the cumulative ranking curve (SUCRA) and rankogram: 0 denoted the worst treatment regimen (certainly the least efficacious or toxic treatment) and 1 denoted the best treatment regimen.

To ensure the transitivity of the key assumptions underlying the network meta-analysis, we included studies with strict patient allocation and an optimised balance for the same conditions. The transitivity assumption underlying indirect comparisons was evaluated using bayesian meta-regression analyses, which examined the potential modifying effects of covariates, including sample size, study design, data source, mutation type, treatment history, and publication type. Inconsistency was evaluated locally by comparing the pooled estimates from network and pairwise meta-analyses, and globally by comparing the fit and parsimony of consistency and inconsistency models using the deviance information criterion value.^28^ Inconsistencies in the entire network were also assessed using the node splitting method, in which direct and indirect evidence were compared separately.

### Patient and public involvement

No patients or members of the public were directly involved in this study as it was a secondary analysis of existing trial data. Limited funding, methodological complexity of network meta-analysis, and time constraints precluded formal patient and public involvement engagement. Importantly, this research directly addresses priorities identified by patients with advanced *BRAF*-mutated colorectal cancer, specifically, the need for rigorous efficacy and safety evaluations of targeted regimens to optimise treatment outcomes.

## Results

**Fig 1:**
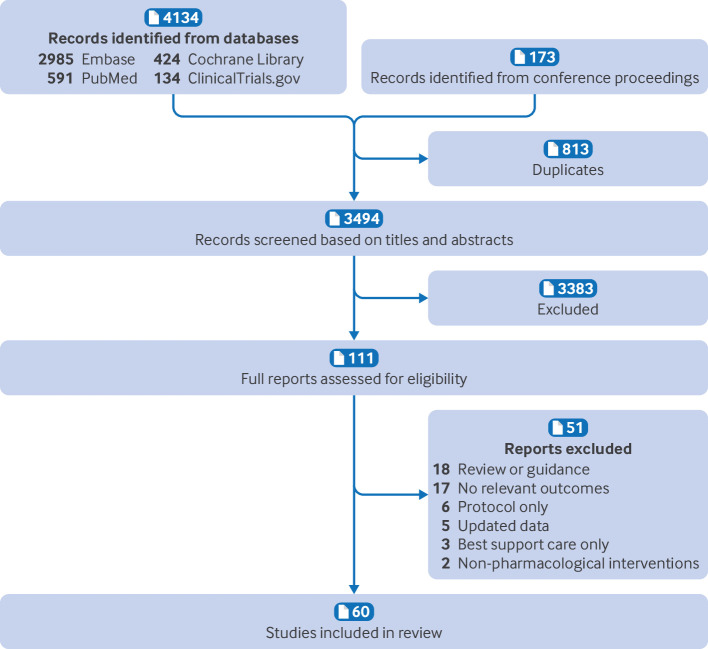
Flow chart of studies in systematic review and meta-analysis

Figure 1 shows the flow chart of studies through this systematic review and meta-analysis. We retrieved a total of 4134 citations from the databases and identified 173 records from conference proceedings. After the removal of duplicate records and the screening of abstracts and titles, 111 studies were further assessed through a full text review. Overall, 60 studies were eligible for inclusion: 25 single arm clinical trials,^5^ ^14^ ^29^ ^30^ ^31^ ^32^ ^33^ ^34^ ^35^ ^36^ ^37^ ^38^ ^39^ ^40^ ^41^ ^42^ ^43^ ^44^ ^45^ ^46^ ^47^ ^48^ ^49^ ^50^ ^51^ 28 controlled clinical trials,^4^ ^9^ ^10^ ^11^ ^18^ ^19^ ^20^ ^52^ ^53^ ^54^ ^55^ ^56^ ^57^ ^58^ ^59^ ^60^ ^61^ ^62^ ^63^ ^64^ ^65^ ^66^ ^67^ ^68^ ^69^ ^70^ ^71^ ^72^ and seven multicentre real world studies,^73^ ^74^ ^75^ ^76^ ^77^ ^78^ ^79^ comprising 4633 patients with advanced *BRAF*-mutated colorectal cancer (see supplementary table S7).

### First line setting

Of the 60 included studies, 32 focused on first line therapy in 2460 patients with advanced *BRAF*-mutated colorectal cancer. The regimens were categorised into 12 types (see supplementary table S8, also see box 1 for definitions of abbreviations): EGFR inhibitor based combinations (doublet chemotherapy-anti-EGFR, triplet chemotherapy-anti-EGFR), VEGF inhibitor based combinations (doublet chemotherapy-anti-VEGF, triplet chemotherapy-anti-VEGF), EGFR/VEGF inhibitor based combinations (doublet chemotherapy-anti-EGFR/VEGF), EGFR/BRAF inhibitor based combinations (single agent chemotherapy-anti-EGFR/BRAF, doublet chemotherapy-anti-EGFR/BRAF), chemotherapy only regimens (doublet or triplet chemotherapy only), targeted therapy only regimens (anti-EGFR/BRAF, anti-EGFR/BRAF/MEK), and VEGF/PD-1 inhibitor based combinations. Regimens for triplet chemotherapy only and VEGF/PD-1 inhibitor based combinations were excluded from subsequent meta-analyses owing to fewer than 10 patients in each regimen.

### Single arm meta-analysis

*Objective response rate*—In the single arm meta-analysis, 25 studies evaluated objective response rate and 11 evaluated disease control rate in a first line setting. Overall, the pooled objective response rate across all regimens was 44.2% (95% CrI 37.7% to 50.8%). For individual regimens, the pooled objective response rates were 39.7% (30.5% to 49.7%) for anti-EGFR based combinations, 42.7% (37.5% to 47.9%) for anti-VEGF based combinations, and 65.8% (58.2% to 72.7%) for anti-EGFR/BRAF based combinations. Doublet chemotherapy-anti-EGFR/BRAF showed the highest objective response rate (67.0%, 58.3% to 74.6%), followed by single agent chemotherapy-anti-EGFR/BRAF (62.0%, 45.3% to 76.2%) (see supplementary figure S3).

*Disease control rate*—The pooled estimates for disease control rate were 73.2% (95% CrI 63.0% to 81.3%) for anti-EGFR based regimens, 73.1% (66.9% to 78.6%) for anti-VEGF based regimens, and 87.7% (81.1% to 92.3%) for anti-EGFR/BRAF based regimens.

*Adverse events*—Doublet chemotherapy-anti-EGFR/BRAF was associated with the highest risk of grade ≥3 adverse events, and anti-EGFR/BRAF was associated with the lowest risk.

### Pairwise meta-analysis

A pairwise meta-analysis was conducted for the overall survival, progression-free survival, and objective response rate in the first line setting (see supplementary figure S4).

*Overall survival*—No statistically significant differences were observed in the available head-to-head comparison analysis for overall survival (triplet chemotherapy-anti-VEGF versus doublet chemotherapy-anti-VEGF, doublet chemotherapy-anti-EGFR versus doublet chemotherapy, doublet chemotherapy-anti-VEGF versus doublet chemotherapy, and doublet chemotherapy-anti-EGFR versus doublet chemotherapy-anti-VEGF).

*Progression-free survival*—Anti-EGFR/BRAF combination regimens (single agent chemotherapy-anti-EGFR/BRAF) showed significantly prolonged progression-free survival than anti-VEGF regimens (doublet chemotherapy-anti-VEGF) (hazard ratio 0.55, 95% CrI 0.35 to 0.85).

*Objective response rate*—Single agent chemotherapy-anti-EGFR/BRAF regimens showed a significantly higher objective response rate than doublet chemotherapy-anti-VEGF (odds ratio 5.17, 95% CrI 2.51 to 10.7) regimens.

### Network meta-analysis

**Fig 2:**
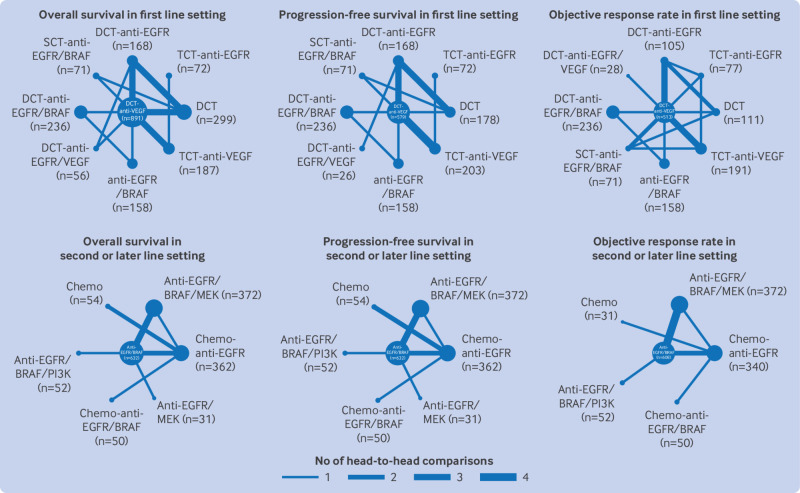
Eligible comparisons for efficacy outcomes of first line and second or later line regimens in network meta-analysis. Network plots illustrate direct and indirect comparisons for overall survival, progression-free survival, and objective response rate. Nodes represent each regimen, with size proportional to number of participants. Lines represent direct comparisons between regimens, with thickness indicating number of direct comparisons. Indirect comparisons arose from combining direct comparisons within the network. BRAF=B-Raf proto-oncogene, serine/threonine kinase; Chemo=chemotherapy; DCT=doublet chemotherapy; EGFR=epidermal growth factor receptor; MEK=mitogen-activated protein kinase kinase; PI3K=phosphoinositide 3-kinase; TCT=triplet chemotherapy; VEGF=vascular endothelial growth factor

Overall, 20 studies (n=2138) with nine first line regimens were included in the network meta-analysis for overall survival, 19 studies (n=1691) with nine first line regimens in the network meta-analysis for progression-free survival, and 17 studies (n=1490) with nine first line regimens in the network meta-analysis for objective response rate (fig 2).

**Fig 3:**
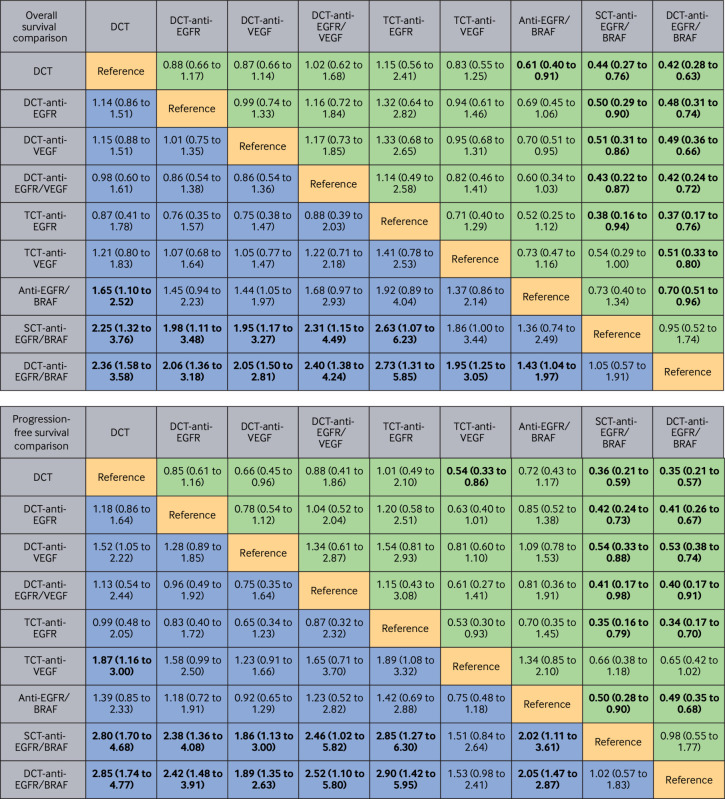
Pooled efficacy estimates of multiple comparisons in first line setting based on network meta-analysis. Data are pooled hazard ratios (95% credible intervals) for overall survival and progression-free survival. Bold data indicate statistical significance. BRAF=B-Raf proto-oncogene, serine/threonine kinase; Chemo=chemotherapy; DCT=doublet chemotherapy; EGFR=epidermal growth factor receptor; TCT=triplet chemotherapy; VEGF=vascular endothelial growth factor

*Overall survival*—Anti-EGFR/BRAF based regimens were found to be the most effective therapeutic approaches. Both doublet chemotherapy-anti-EGFR/BRAF and single agent chemotherapy-anti-EGFR/BRAF had comparable overall survival benefits (hazard ratio 0.95, 95% CrI 0.52 to 1.74), showing significant superior benefit compared with other regimens. Doublet chemotherapy-anti-EGFR/BRAF yielded the best overall survival, with significant benefits compared with doublet chemotherapy (hazard ratio 0.42, 95% CrI 0.28 to 0.63), doublet chemotherapy-anti-EGFR (0.48, 0.31 to 0.74), doublet chemotherapy-anti-VEGF (0.49, 0.36 to 0.66), doublet chemotherapy-anti-EGFR/VEGF (0.42, 0.24 to 0.72), triplet chemotherapy-anti-EGFR (0.37, 0.17 to 0.76), triplet chemotherapy-anti-VEGF (0.51, 0.33 to 0.80), and anti-EGFR/BRAF (0.70, 0.51 to 0.96) regimens. Similarly, single agent chemotherapy-anti-EGFR/BRAF regimens showed a statistically significant advantage in overall survival benefits compared with the alternative treatment strategies (doublet chemotherapy: hazard ratio 0.44, 95% CrI 0.27 to 0.76, doublet chemotherapy-anti-EGFR: 0.50, 0.29 to 0.90, doublet chemotherapy-anti-VEGF: 0.51, 0.31 to 0.86, doublet chemotherapy-anti-EGFR/VEGF: 0.43, 0.22 to 0.87, triplet chemotherapy-anti-EGFR: 0.38, 0.16 to 0.94) (fig 3).

*Progression-free survival*—Anti-EGFR/BRAF based regimens (doublet chemotherapy-anti-EGFR/BRAF and single agent chemotherapy-anti-EGFR/BRAF) also showed a statistically significant improvement in progression-free survival compared with traditional anti-EGFR based or anti-VEGF based combinations, as well as anti-EGFR/BRAF (hazard ratio 0.49, 95% CrI 0.35 to 0.68; 0.50, 0.28 to 0.90; respectively) (fig 3).

*Objective response rate and disease control rate*—Objective response rate showed improvement with dual inhibition of EGFR and BRAF compared with the EGFR based or VEGF based combination regimens (see supplementary figures S5 and S6). A similar trend of high disease control rate was observed for the anti-EGFR/BRAF based regimen; the difference was not, however, significant.

*Adverse events*—Anti-EGFR/BRAF showed a more favourable safety for grade ≥3 adverse events than other strategies, although without statistical significance.

### Bayesian ranking profile

**Fig 4:**
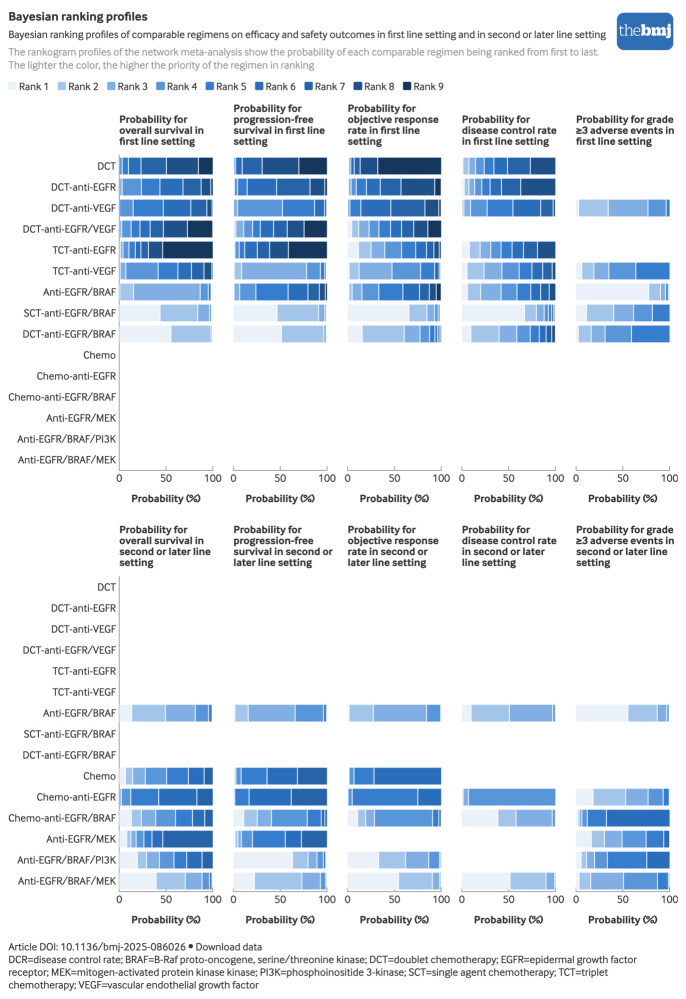
Bayesian ranking profiles of comparable regimens on efficacy and safety outcomes in first line setting and second or later line setting. An interactive version of this graphic is available at https://public.flourish.studio/visualisation/25802694/

**Fig 5:**
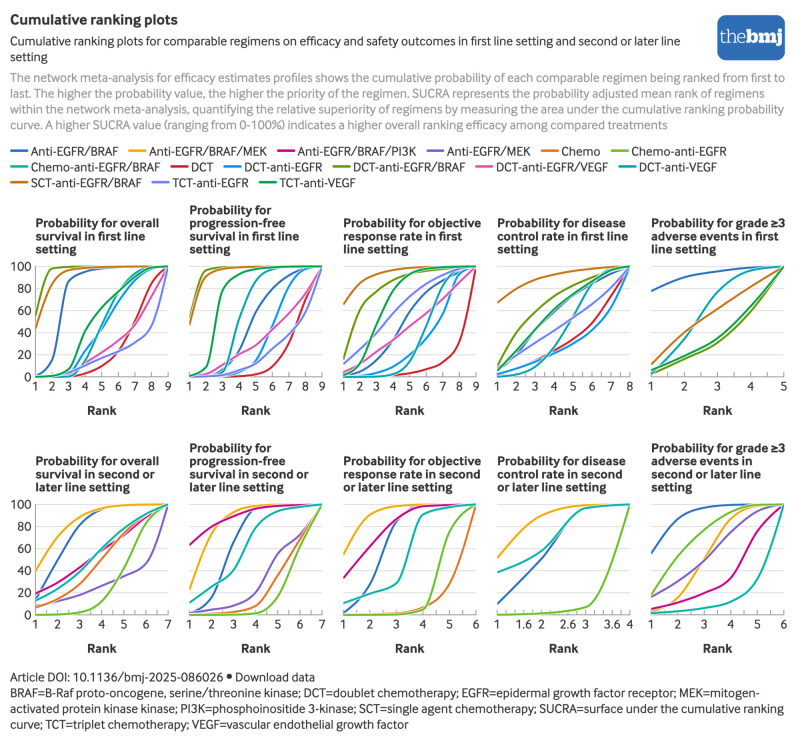
Cumulative ranking plots for comparable regimens on efficacy and safety outcomes in first line setting and second or later line setting. An interactive version of this graphic is available at https://public.flourish.studio/visualisation/25803421/

The bayesian ranking profiles of regimens were almost consistent with those of the pooled analyses using hazard ratios and odd ratios, including rankogram (fig 4), and SUCRA (fig 5). Anti-EGFR/BRAF based regimens consistently ranked among the top two in efficacy outcomes: doublet chemotherapy-anti-EGFR/BRAF showed the highest overall survival and progression-free survival (SUCRA 0.94 and 0.93, respectively) followed by single agent chemotherapy-anti-EGFR/BRAF (0.90 and 0.92, respectively), whereas single agent chemotherapy-anti-EGFR/BRAF achieved superior objective response rate and disease control rate (0.92 and 0.88, respectively). In the safety analysis, anti-EGFR/BRAF showed significant superiority, whereas doublet chemotherapy-anti-EGFR/BRAF ranked last (0.91 and 0.27, respectively) among the five regimens with reported grade ≥3 adverse events.

### Second or later line setting

Thirty studies investigated second or later line therapies for advanced *BRAF*-mutated colorectal cancer in a pooled population of 2173 patients (see supplementary table S7). The second or later line regimens were categorised into 14 types (see supplementary table S8): chemotherapy, chemotherapy-EGFR inhibitor (chemotherapy-anti-EGFR), chemotherapy-EGFR and BRAF inhibitor (chemotherapy-anti-EGFR/BRAF), chemotherapy-EGFR, BRAF, and MEK inhibitors (chemotherapy-anti-EGFR/BRAF/MEK), BRAF inhibitor (anti-BRAF), BRAF inhibitor plus MEK inhibitor (anti-BRAF/MEK), EGFR inhibitor-MEK inhibitor (anti-EGFR/MEK), EGFR inhibitor-BRAF inhibitor (anti-EGFR/BRAF), and anti-EGFR/BRAF with MEK inhibitor (anti-EGFR/BRAF/MEK), with PI3K inhibitor (anti-EGFR/BRAF/PI3K), with ERK inhibitor (anti-EGFR/BRAF/ERK), with WNT inhibitor (anti-EGFR/BRAF/WNT), and with PD-1 inhibitor (anti-EGFR/BRAF/PD-1), and BRAF inhibitor-MEK inhibitor with PD-1 inhibitor (anti-BRAF/MEK/PD-1).

### Single arm meta-analyses

In the single arm meta-analysis, 29 studies evaluated objective response rate and 24 evaluated control rate in second line or later line settings. Overall, the pooled objective response rate across all regimens was 22.6% (95% CrI 19.0% to 26.6%) (see supplementary figure S7). The pooled objective response rate was highest for the anti- EGFR/BRAF/PD-1 regimen (36.9% 18.7% to 59.8%), followed by chemotherapy-anti-EGFR/BRAF (33.3%, 9.0% to 71.5%) and anti-EGFR/BRAF/MEK (27.1%, 21.8% to 33.1%). Similar trends were observed in the meta-analysis of disease control rate, with anti-EGFR/BRAF/MEK showing the highest disease control rate (76.9%, 95% CrI 70.4% to 82.3%), followed by anti-EGFR/BRAF/PD-1 (75.8%, 51.7% to 90.1%) and anti-EGFR/BRAF (69.9%, 64.4% to 75.0%).

Pooled data showed that the incidence of grade ≥3 adverse events was 39.9% (95% CrI 24.7% to 57.4%) for anti-EGFR/BRAF, compared with 48.6% (24.0% to 73.9%) for anti-EGFR/BRAF/MEK. Chemotherapy-anti-EGFR and anti-BRAF/MEK showed a relatively higher risk of grade ≥3 adverse events (54.9%, 46.8% to 62.7% and 52.0%, 36.0% to 67.7%, respectively).

### Pairwise meta-analysis

Pairwise meta-analysis indicated anti-EGFR/BRAF was associated with significantly prolonged overall survival and progression-free survival than chemotherapy-anti-EGFR (overall survival: hazard ratio 0.60, 95% CrI 0.48 to 0.75; progression-free survival: 0.43, 0.35 to 0.53) (see supplementary figure S8). Anti-EGFR/BRAF/MEK could provide significantly higher objective response rate benefit than anti-EGFR/BRAF (odds ratio 1.57, 95% CrI 1.10 to 2.24), but not disease control rate (1.20, 0.84 to 1.73). In contrast, anti-EGFR/BRAF/MEK was associated with a significantly higher risk of grade ≥3 adverse events than anti-EGFR/BRAF (1.51, 1.10 to 2.07).

### Network meta-analysis

Nine studies (n=1553) with seven regimens were incorporated into the network-meta-analysis for overall survival and progression-free survival, and eight studies (n=1453) with six regimens were incorporated into the network meta-analysis for objective response rate (fig 2).

**Fig 6:**
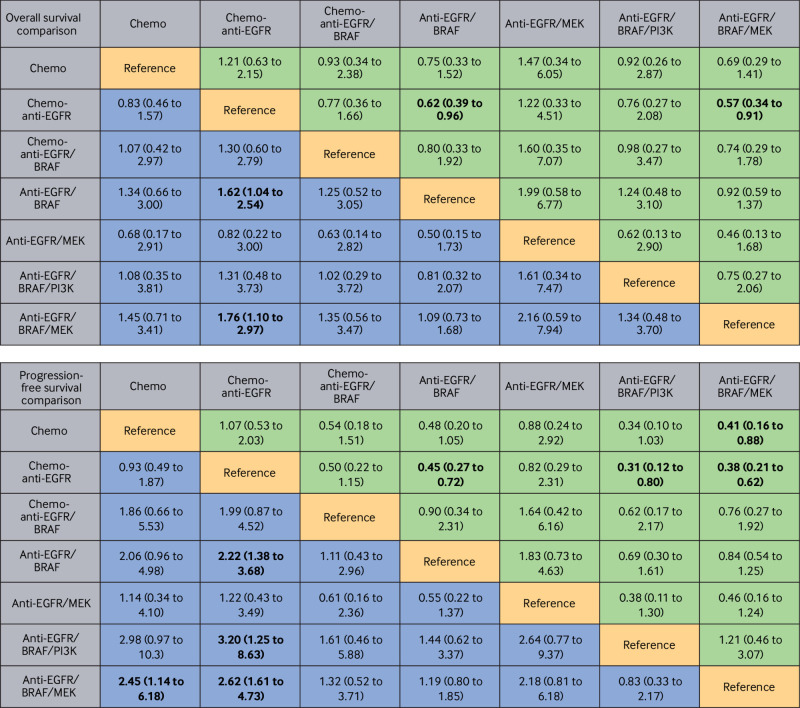
Pooled efficacy estimates of multiple comparisons in second line or later line settings based on network meta-analysis. Data are pooled hazard ratios (95% credible intervals) for overall survival and progression-free survival. Bold data indicate statistical significance. BRAF=B-Raf proto-oncogene, serine/threonine kinase; EGFR=epidermal growth factor receptor; MEK=mitogen-activated protein kinase kinase; PI3K=phosphoinositide 3-kinase; VEGF=vascular endothelial growth factor

*Overall survival and progression-free survival*—Anti-EGFR/BRAF based regimens provided better overall survival and progression-free survival (fig 6). Both anti-EGFR/BRAF and anti-EGFR/BRAF/MEK were associated with significantly prolonged overall survival than chemotherapy-anti-EGFR (hazard ratio 0.62, 95% CrI 0.39 to 0.96 and 0.57, 0.34 to 0.91, respectively). Similar findings could be observed in progression-free survival analysis (0.45, 0.27 to 0.72 and 0.38, 0.21 to 0.62). Anti-EGFR/BRAF/MEK showed no statistically significant differences in overall survival (0.92, 0.59 to 1.37) and progression-free survival (0.84, 0.54 to 1.25) compared with anti-EGFR/BRAF. In contrast, anti-EGFR/MEK showed a relatively higher mortality risk than other regimens. For patients with previous use of traditional therapies, the addition of chemotherapy to anti-EGFR/BRAF did not significantly improve overall survival (1.25, 0.52 to 3.05) and progression-free survival (1.11, 0.43 to 2.96). Compared with chemotherapy-EGFR, the risk of disease progression was also significantly reduced with anti-EGFR/BRAF/PI3K (0.31, 0.12 to 0.80), but anti-EGFR/BRAF/PI3K showed inferior overall survival outcomes compared with both anti-EGFR/BRAF/MEK and anti-EGFR/BRAF.

*Objective response rate*—Anti-EGFR/BRAF based regimens (anti-EGFR/BRAF, anti-EGFR/BRAF/PI3K, and anti-EGFR/BRAF/MEK) also significantly improved objective response rate compared with traditional regimens, but the addition of chemotherapy to anti-EGFR/BRAF was not associated with additional significant objective response rate benefit (see supplementary figure S9).

*Disease control rate*—Anti-EGFR/BRAF/MEK was associated with a higher disease control rate than chemotherapy-anti-EGFR (odds ratio 8.19, 95% CrI 1.42 to 55.9).

*Adverse events*—Anti-EGFR/BRAF was associated with a lower risk of grade ≥3 adverse events, although differences did not reach statistical significance.

### Bayesian ranking profile

Bayesian ranking analyses of targeted therapy based regimens in second line or later line setting was also presented in rankogram (fig 4) and SUCRA (fig 5). Notably, anti-EGFR/BRAF based regimens consistently ranked among the top two for overall survival outcomes: anti-EGFR/BRAF/MEK showed the highest overall survival (SUCRA 0.82), objective response rate (0.89), and disease control rate (0.80) followed by anti-EGFR/BRAF (0.73) for overall survival, anti-EGFR/BRAF/PI3K (0.76) for objective response rate, and chemotherapy-anti-EGFR/BRAF (0.64) for disease control rate. Anti-EGFR/BRAF/PI3K achieved the best progression-free survival (SUCRA=0.88), followed by anti-EGFR/BRAF/MEK (0.81). In the safety profile analysis, anti-EGFR/BRAF showed significant superiority, ranking first (0.88) among the six regimens with reported grade ≥3 adverse events.

### Meta-regression analysis and sensitivity analysis

Meta-regression analysis confirmed that the variables—including sample size, study design, data source, mutation type, treatment history, and publication type—had no statistically significant effect on the final pooled estimates, thereby further supporting the acceptance of the transitivity assumption (see supplementary table S9). Similarly, in sensitivity analysis after excluding data from conferences, the results showed no significant influence on the pooled estimates, further supporting the strength of these findings (see supplementary figure S10). The goodness of fit between the consistency and inconsistency models based on the deviance information criterion values confirmed the consistency of the results (see supplementary table S10). Moreover, the node splitting method analysis indicated no significant inconsistency between direct and indirect estimates for most available comparisons. However, for certain comparisons, particularly those involving objective response rate, disease control rate, and adverse events (eg, anti-EGFR/BRAF/MEK versus anti-EGFR/BRAF), the network structure lacked independent indirect evidence pathways, making a valid node split evaluation unfeasible (see supplementary figure S11).

### Risk of bias assessment

Risk of bias assessments for all included studies are summarised in supplementary figures S12-S15. In the ROB 2 assessment of randomised controlled trials, some study data were derived from the results of subgroup analysis. Although the randomisation process was satisfactory in the overall study population, it remains unknown whether randomisation was properly maintained and whether baseline characteristics were balanced specifically within subgroups with the *BRAF* mutation. Consequently, assessors rated these studies as having “some concerns” in the domain of randomisation process. In addition, the results for CINeMA evidence grading showed variable confidence ratings across treatment comparisons and outcomes. Most comparisons in overall survival and progression-free survival achieved high and moderate confidence, whereas others such as comparison in objective response rate were rated low, even very low due to major concerns in imprecision and incoherence. Mixed comparisons evidence in first line settings showed predominantly moderate-to-high confidence, whereas evidence from indirect comparisons and second line or later line analyses frequently showed low confidence (see supplementary table S11).

## Discussion

This systematic review comprehensively summarised the efficacy and safety of currently available targeted therapy strategies for advanced *BRAF*-mutated colorectal cancer. The pooled estimates from the single arm, pairwise, and network meta-analyses were highly consistent, indicating that patients could benefit from anti-EGFR/BRAF based regimens. Particularly, anti-EGFR/BRAF in combination with chemotherapy has shown encouraging antitumor activity, with significant improvements in progression-free survival and overall survival compared with other targeted therapy strategies in the first line setting. Anti-EGFR/BRAF, with or without an additional inhibitor (anti-MEK or anti-PI3K), also showed better efficacy in second line or later line settings, especially compared with traditional chemotherapy-anti-EGFR. For safety outcomes, our analysis found that first line anti-EGFR/BRAF regimens combined with chemotherapy were associated with an increased risk of grade ≥3 adverse events; however, this difference was not statistically significant. In contrast, chemotherapy-free anti-EGFR/BRAF regimens showed improved safety profiles in both first line and second line or later line settings.

Our review investigated a targeted therapy strategy for advanced *BRAF*-mutated colorectal cancer in a large population, comprising 4633 patients, providing comparative analyses of 12 first line regimens and 14 second line regimens. These findings reinforce the established role of anti-EGFR/BRAF based regimens in advanced *BRAF*-mutated colorectal cancer, challenging the traditional first line standard of care, doublet or triplet chemotherapy combined with anti-VEGF regimens, and further validating the clinical utility of anti-EGFR/BRAF strategies as later line therapy for this molecularly defined population.

### Targeted therapy

Chemotherapy combined with anti-EGFR or anti-VEGF agents has become the standard of care for advanced colorectal cancer, guided by molecular profiling and the anatomical characteristics of tumours. Targeted therapy based combinations are critical for advanced *BRAF*-mutated colorectal cancer. We found that chemotherapy alone underperformed compared with targeted regimens across all key efficacy endpoints in both first line and second line or later line settings. For instance, pooled analysis revealed an objective response rate of 39.7-65.8% for targeted combinations, surpassing the efficacy of chemotherapy alone in the first line setting. Similar trends were observed for overall survival, reinforcing the need for targeted therapy in this population.

### Optimal selection of targeted therapy

Targeted therapy for *BRAF*-mutated colorectal cancer has evolved from conventional anti-VEGF/anti-EGFR based regimens to BRAF targeted therapy; however, none achieved substantial clinical benefits until the BEACON trial established the efficacy of dual EGFR/BRAF inhibition. The underlying mechanism is the incomplete suppression of the oncogenic signalling pathway due to rapid EGFR mediated feedback activation when using a BRAF inhibitor alone. Dual inhibition of BRAF and EGFR significantly enhanced antitumor activity. Our study further validated that the rational selection of targeted agents is critical for optimising outcomes. EGFR/BRAF inhibitor based regimens as first line therapy showed superior efficacy compared with anti-VEGF based or anti-EGFR based strategies, with significant improvements in overall survival and progression-free survival. Anti-EGFR/BRAF regimens outperformed other targeted combinations in pretreated patients, reinforcing that EGFR and BRAF are pivotal therapeutic targets for *BRAF*-mutated colorectal cancer.

The anti-EGFR/BRAF regimen retains a therapeutic cornerstone even when integrated with additional modalities. Emerging evidence suggests that combining PI3K, ERK, or MEK inhibitors with anti-EGFR/BRAF therapy may theoretically overcome residual resistance by targeting the downstream BRAF signalling pathways. Pooled analysis revealed that the anti-EGFR/BRAF regimen exhibited effective antitumor activity and served as a therapeutic backbone. The anti-EGFR/BRAF/MEK regimens showed a non-significant trend toward superior efficacy compared with dual EGFR/BRAF inhibition. Specifically, pairwise meta-analysis indicated that adding a MEK inhibitor significantly improved the objective response rate, but this significant benefit was not confirmed in network meta-analyses. Although insufficient survival data precluded comprehensive assessment of another inhibitor to anti-EGFR/BRAF backbones, our analyses suggest potential improvements in efficacy with these combinations, especially anti-EGFR/BRAF/MEK. Notably, the observed efficacy trends coincided with increased risks of toxicity, particularly relevant in pretreated populations where the safety-efficacy balance is crucial. These findings carry important implications for the design of future trials. In addition, the most promising synergistic antitumor effect emerged from the combination of PD-1 inhibitors and EGFR/BRAF blockade,^80^ ^81^ ^82^ which was associated with the highest objective response rate across the evaluated regimens. Combining EGFR/BRAF inhibition with PD-1 blockade achieved superior clinical outcomes compared with BRAF/MEK/PD-1 combinations, underscoring the critical importance of EGFR/BRAF co-targeting in optimising both independent and immune enhanced therapeutic strategies.

### Role of chemotherapy combinations

Our findings also underscore the important role of chemotherapy combined with anti-EGFR/BRAF-targeted therapy in the first line setting. The network meta-analysis showed significantly superior progression-free survival and overall survival with chemotherapy combined with anti-EGFR/BRAF compared with anti-EGFR/BRAF alone. This finding showed high consistency with the direct comparative evidence from the BREAKWATER study. Additionally, results from the ANCHOR study evaluating the anti-EGFR/BRAF/MEK triplet regimen provide indirect support for the significance of chemotherapy in first line treatment. Our investigation also provides the first systematic evaluation of differential intensities of chemotherapy combined with anti-EGFR/BRAF therapy, a previously unexplored area, specifically analysing the comparative efficacy and safety profiles in this context. Although no statistically significant differences were observed between single agent and dual agent chemotherapy combined with anti-EGFR/BRAF therapy, dual agent regimens showed higher efficacy at the cost of increased adverse events. This finding necessitates a careful balance between efficacy and safety in first line treatment, with dual agent chemotherapy recommended for patients with adequate tolerance and single agent chemotherapy remaining a viable alternative for those at a higher risk of toxicity. In contrast, chemotherapy-free anti-EGFR/BRAF based regimens emerged as the preferred therapeutic strategy in pretreated patients, as pooled analyses indicated no improved benefit from added toxicity with chemotherapy combinations after first line therapy, although these trends lacked statistical significance. Collectively, our study found that dual target inhibition of EGFR/BRAF drives the core antitumor activity in *BRAF*-mutated colorectal cancers. In chemotherapy-naïve populations in the first line setting, the addition of chemotherapy further enhances efficacy, although the optimal intensity requires further investigation. Conversely, the necessity for chemotherapy diminished in previously treated patients, with no benefit observed for intensified regimens beyond targeted therapy in this setting. Overall, our data suggest a progressive de-escalation of chemotherapy’s role across treatment lines among patients with advanced *BRAF*-mutated colorectal cancer, highlighting implications for the design of future trials.

### Early initiation of targeted therapy

Our findings established the anti-EGFR/BRAF based regimen as optimal strategy for patients with *BRAF*-mutated colorectal cancer in both first line and second line settings. The approval of anti-EGFR/BRAF regimens for second line treatment of *BRAF*-mutated colorectal cancer underscores its clinical significance. However, a critical question remains as to whether early initiation of anti-EGFR/BRAF therapy confers superior outcomes compared with delayed use in later lines. Although head-to-head comparative trials are lacking, our data revealed notable differences in efficacy. For instance, frontline chemotherapy combined with anti-EGFR/BRAF achieved a superior objective response rate of 65.8% versus 33.3% in second line or later line settings. Network meta-analysis showed significantly greater reductions in both disease progression and mortality when used in the first line setting compared with later line use. These findings indirectly support the prioritisation of anti-EGFR/BRAF regimens in first line settings.

### Safety profile of targeted therapy based regimen

Safety assessments showed that anti-EGFR/BRAF combinations with chemotherapy in the first line setting were associated with higher rates of grade ≥3 adverse events but were not significantly different from traditional anti-VEGF based regimens. Anti-EGFR/BRAF showed a favourable safety profile, with adverse event rates significantly lower than those of chemotherapy containing regimens. However, the addition of a third targeted agent (eg, PI3K inhibitors) increased the risk of toxicity, underscoring the need to balance efficacy and tolerability. For instance, frontline strategies may prioritise intensive chemotherapy regimens, whereas later line therapy may favour low toxicity anti-BRAF/EGFR alone, as reflected in recent guideline updates.

### Strengths and limitations of this review

Previous meta-analyses investigating first line therapies for *BRAF*-mutated colorectal cancer have provided limited insights. For example, a meta-analysis of individual patient data comparing triplet chemotherapy-anti-VEGF with doublet chemotherapy-anti-VEGF showed no significant differences in efficacy in subgroups with *BRAF* mutations, a finding consistent with our observations.^12^ Similarly, another meta-analysis confirmed the value of doublet chemotherapy-anti-VEGF combinations in first line settings.^13^ However, these analyses were constrained by narrow therapeutic contexts (eg, first line comparisons of anti-VEGF with anti-EGFR regimens), small sample sizes, and the exclusion of emerging therapeutic strategies such as dual or triple target inhibition and integration of immunotherapy. In contrast, our study dealt with these limitations through several key innovations. Firstly, we incorporated the most recent data covering new and updated information (to May 2025), enabling comprehensive evaluation of both first line and subsequent line targeted therapies in *BRAF*-mutated colorectal cancer. Secondly, beyond comparisons of conventional chemotherapy backbones, we analysed novel therapeutic strategies, such as chemotherapy-anti-EGFR/BRAF and targeted immunotherapy, reflecting current clinical trial priorities. Thirdly, integrating data from randomised controlled trials, single arm trials, and high quality real world evidence using diverse meta-analytical methods substantially strengthened the validity of the results while capturing new developments in therapy.

Our study has several limitations. Firstly, the analysis relied exclusively on trial level rather than individual patient data. Variations in the study design and potential heterogeneity across study populations might have introduced bias, although the meta-regression analyses and sensitivity analysis indicated that these factors did not substantially influence the pooled results. Secondly, several previous trials investigating *BRAF*-mutated colorectal cancer primarily reported subgroup analyses from landmark studies with limited sample sizes, which could affect the reliability of the meta-analytical estimates; however, meta-regression also showed no substantial effect. Thirdly, data sparsity due to the paucity of trials and participants may have affected the reliability of the findings. For several therapeutic regimens in both first line and second or later line settings, only single arm study data were available, permitting analyses of objective response rate and disease control rate but precluding comparative evaluations of progression-free survival or overall survival, thereby limiting interpretability. Furthermore, six of the 60 eligible studies enrolled patients with both *BRAF V600E* and other subtypes. Although non-V600E mutations constituted a minimal proportion of the total study population, and further analysis excluded their considerable influence on pooled outcomes, stratified analysis by molecular characteristics was precluded by insufficient data. Finally, most patients in the second line or later line studies did not receive previous anti-EGFR/BRAF dual target therapy, suggesting that these findings primarily applied to populations treated with conventional regimens. Therefore, optimal sequencing strategies for patients treated with first line anti-EGFR/BRAF targeted combinations warrant further exploration.

### Conclusion

Using multiple meta-analysis approaches, we found that anti-EGFR/BRAF based regimens represent an optimal therapeutic strategy for patients with advanced *BRAF*-mutated colorectal cancer. The integration of chemotherapy with EGFR/BRAF targeted therapies is essential to maximise therapeutic efficacy in chemotherapy-naïve patients, albeit at the cost of increased toxicity. For pretreated patients, anti-EGFR/BRAF therapy without chemotherapy is the preferred strategy, balancing efficacy with reduced toxicity. These findings highlight the necessity of line specific therapeutic decision making and clarify the roles of anti-EGFR/BRAF based treatment strategies in patients with advanced *BRAF*-mutated colorectal cancer, potentially complementing the current guidelines.

**What is already known on this topic**

Patients with advanced *BRAF*-mutated colorectal cancer often have a poor prognosis and suboptimal response to conventional therapies
While multiple targeted strategies have been explored, substantial improvements in treatment efficacy were observed after the introduction of anti-EGFR/BRAF based regimens
Owing to the relatively low incidence of *BRAF*-mutated colorectal cancer, however, direct comparisons of the efficacy and safety of these targeted regimens remain limited

**What this study adds**

In this systematic review and meta-analysis, first line chemotherapy-anti-EGFR/BRAF regimens were found to offer optimal efficacy and acceptable safety for *BRAF*-mutated colorectal cancer
The anti-EGFR/BRAF regimen also showed promising efficacy and tolerability in second line or later line settings
The addition of an additional targeted agent to anti-EGFR/BRAF, such as anti-EGFR/BRAF/MEK, should ensure a balance between efficacy and safety

## Data Availability

Data and code for reproducing analyses are available on Github (https://github.com/qbdchnontheway/code/tree/main/Metaanalyses_code_for_BRAFmut_CRC).

## Web extra

Extra material supplied by authors

Supplementary information: Tables S1-S12, figures S1-S15, and supplementary note 1

## Notes

Contributors: B-DQ and Y-SZ conceived the study and drafted the study protocol. They are the guarantors. B-DQ, X-DJ, ZW, and KL performed the literature search, selected the studies, and extracted data. B-DQ, X-DJ, YL, and YW performed the statistical analysis. KL, YL, and YW conducted the risk of bias assessment. B-DQ, X-DJ, ZW, and KL drafted the manuscript and took responsibility for critical revision. B-DQ, X-DJ, ZW, and KL contributed equally to this work. All authors reviewed and approved the final manuscript, and the guarantors have agreed to be accountable for all aspects of the work in ensuring that questions related to the accuracy or integrity of any part of the work are appropriately investigated and resolved. The corresponding author attests that all listed authors meet authorship criteria and that no others meeting the criteria have been omitted.

Transparency: The corresponding author (Y-SZ) affirms that the manuscript is an honest, accurate, and transparent account of the study being reported; that no important aspects of the study have been omitted; and that any discrepancies from the study as registered have been explained.

Dissemination to participants and related patient and public communities: We plan to present our findings and discuss them with the public through social media and at conferences attended by patient groups and advocacy organisations. Plain language summaries of key findings will be disseminated through social media platforms.

Provenance and peer review: Not commissioned; externally peer reviewed.
