# Supplementary material for: Targeted therapy in advanced BRAF-mutated colorectal cancer: systematic review and network meta-analysis
Source: BMJ. 2025 Nov 19;391:e086026. doi: 10.1136/bmj-2025-086026 (PMC12628362; doi:10.1136/bmj-2025-086026)
Supplement: Supplementary file 1 — Supplementary information: Tables S1-S12, figures S1-S15, and supplementary note 1 [file donb086026.ww.pdf]

Supplementary appendix

Table S1. Checklist of the PRISMA extension for network meta-analysis.

Table S2. Literature search strategy.

Table S3. MINORS evaluation Criteria: Risk of bias assessments for the single-arm trials and non-randomised comparative studies.

Table S4. Cochrane Risk of Bias tool version 2 (RoB 2.0) for randomised controlled trials.

Table S5. ArRoWS evaluation criteria: risk of bias assessments for the real-world study.

Table S6. ROBINS-I evaluation criteria: risk of bias assessments for the real-world study.

Table S7. Baseline characteristics of the included studies.

Table S8. Categorization of treatment regimens for first-line, second-line, and later-line therapy

Table S9. Results of network meta-regression analysis assessing potential effect modifiers.

Table S10. Comparison of the fit goodness between consistency and inconsistency models based on DIC values in network meta-analysis.

Table S11. CINeMA Evidence Grading Results

Table S12. Abbreviation list

Figure S1. Assessment of convergence for OS, PFS, ORR, DCR and grade 3 or higher AE in first-line setting using trace plots, density plots, and the Brooks–Gelman–Rubin method.

Figure S2. Assessment of convergence for OS, PFS, ORR, DCR and grade 3 or higher AE in second- or later-line setting using trace plots, density plots, and the Brooks–Gelman–Rubin method.

Figure S3. Pooled efficacy and safety estimates (objective response rate, disease control rate, grade-3 or higher AE) of the first-line regimen in single-arm meta-analysis.

Figure S4. Pooled efficacy estimates (overall survival, OS; progression-free survival, PFS; objective response rate, ORR) of the first-line regimen in head-to-head comparisons in pairwise meta-analysis.

Figure S5. Eligible comparisons for disease control rate and grade 3 or higher AEs in the network meta-analysis.

Figure S6. Pooled efficacy (objective response rate, ORR; disease control rate, DCR) and safety (grade 3 or higher AEs) estimates of multiple comparisons in first-line setting based on the network meta-analysis.

Figure S7. Pooled efficacy and safety estimates (objective response rate, ORR; disease control rate, DCR, grade-3 or higher AE) of the second- or later-line setting in single-arm meta-analysis.

Figure S8. Pooled efficacy and safety estimates (overall survival, OS; progression-free survival, PFS; objective response rate, ORR; disease control rate, DCR; grade 3 or higher AEs) of the second- or later-line regimen in head-to-head comparisons in pairwise meta-analysis.

Figure S9. Pooled efficacy (objective response rate, ORR; disease control rate, DCR) and safety (grade 3 or higher AEs) estimates of multiple comparisons in second- or later--line setting based on the network meta-analysis.

Figure S10. Pooled efficacy and safety estimates (objective response rate, disease control rate, grade-3 or higher AE) in single-arm meta-analysis after excluding conferences reports.

Figure S11. Forest plot of the pooled estimates using direct comparison, indirect comparison, and network meta-analysis via the node-splitting method.

Figure S12. Risk of bias assessment for the included single-arm trials and non-randomised controlled trial according to the MINORS Evaluation Criteria.

Figure S13. Risk of bias assessments for the included randomised controlled trials according to the ROB2 tool.

Figure S14. Risk of bias assessment for the included real-world study according to the ArRoWS Evaluation Criteria.

Figure S15. Risk of bias assessment for the included real-world study according to the ROBINS-I Evaluation Criteria.

Supplementary Note 1. Systematic Review Protocol

Table S1. Checklist of the PRISMA extension for network meta-analysis.

Section/Topic	Item #	Checklist Item	Reported on Page #
**TITLE**			
Title	1	Identify the report as a systematic review *incorporating a network meta-analysis (or related form of meta-analysis).*	Page 1
**ABSTRACT**			
Structured summary	2	Provide a structured summary including, as applicable:<br>**Background:** main objectives<br>**Methods:** data sources; study eligibility criteria, participants, and interventions; study appraisal; and *synthesis methods, such as network meta-analysis.*<br>**Results:** number of studies and participants identified; summary estimates with corresponding confidence/credible intervals; *treatment rankings may also be discussed. Authors may choose to summarize pairwise comparisons against a chosen treatment included in their analyses for brevity.*<br>**Discussion/Conclusions:** limitations; conclusions and implications of findings.<br>**Other:** primary source of funding; systematic review registration number with registry name.	Page 2
**INTRODUCTION**			
Rationale	3	Describe the rationale for the review in the context of what is already known*, including mention of why a network meta-analysis has been conducted.*_	Page 5
Objectives	4	Provide an explicit statement of questions being addressed, with reference to articipants, interventions, comparisons, outcomes, and study design (PICOS).	Page 5-6
**METHODS**			
Protocol and registration	5	Indicate whether a review protocol exists and if and where it can be accessed (e.g., Web address); and, if available, provide registration information, including registration number.	Page 6
Eligibility criteria	6	Specify study characteristics (e.g., PICOS, length of follow-up) and report characteristics (e.g., years considered, language, publication status) used as criteria for eligibility, giving rationale. *Clearly describe eligible treatments included in the treatment*	Page 6

		*network, and note whether any have been clustered or merged into the same node (with justification).*_	
Information sources	7	Describe all information sources (e.g., databases with dates of coverage, contact with study authors to identify additional studies) in the search and date last searched.	Page 6
Search	8	Present full electronic search strategy for at least one database, including any limits used, such that it could be repeated.	Page 6, Table S2
Study selection	9	State the process for selecting studies (i.e., screening, eligibility, included in systematic review, and, if applicable, included in the meta-analysis).	Page 6-7
Data collection process	10	Describe method of data extraction from reports (e.g., piloted forms, independently, in duplicate) and any processes for obtaining and confirming data from investigators.	Page 6-7
Data items	11	List and define all variables for which data were sought (e.g., PICOS, funding sources) and any assumptions and simplifications made.	Page 6-7
Geometry of the network	S1	Describe methods used to explore the geometry of the treatment network under study and potential biases related to it. This should include how the evidence base has been graphically summarized for presentation, and what characteristics were compiled and used to describe the evidence base to readers.	Page 6-7
Risk of bias within individual studies	12	Describe methods used for assessing risk of bias of individual studies (including specification of whether this was done at the study or outcome level), and how this information is to be used in any data synthesis.	Page 6-7
Summary measures	13	State the principal summary measures (e.g., risk ratio, difference in means). *Also describe the use of additional summary measures assessed, such as treatment rankings and surface under the cumulative ranking curve (SUCRA) values, as well as modified approaches used to present summary findings from meta-analyses.*	Page 7-8
Planned methods of analysis	14	Describe the methods of handling data and combining results of studies for each network meta-analysis. This should include, but not be limited to:<br>• *Handling of multi-arm trials;*<br>• *Selection of variance structure;*<br>• *Selection of prior distributions in Bayesian analyses; and*	Page 8

		• *Assessment of model fit.*	
Assessment of Inconsistency	S2	Describe the statistical methods used to evaluate the agreement of direct and indirect evidence in the treatment network(s) studied. Describe efforts taken to address its presence when found.	Page 8
Risk of bias across studies	15	Specify any assessment of risk of bias that may affect the cumulative evidence (e.g., publication bias, selective reporting within studies).	Page 8
Additional analyses	16	Describe methods of additional analyses if done, indicating which were pre-specified. This may include, but not be limited to, the following:<br>• Sensitivity or subgroup analyses;<br>• Meta-regression analyses;<br>• *Alternative formulations of the treatment network; and*<br>• *Use of alternative prior distributions for Bayesian analyses (if applicable).*_	Page 8
**RESULTS†**			
Study selection	17	Give numbers of studies screened, assessed for eligibility, and included in the review, with reasons for exclusions at each stage, ideally with a flow diagram.	Page 9<br>Figure 1
Presentation of network structure	S3	Provide a network graph of the included studies to enable visualization of the geometry of the treatment network.	Page 10<br>Page 12<br>Figure 2<br>Figure S5
Summary of network geometry	S4	Provide a brief overview of characteristics of the treatment network. This may include commentary on the abundance of trials and randomized patients for the different interventions and pairwise comparisons in the network, gaps of evidence in the treatment network, and potential biases reflected by the network structure.	Page 9<br>Page 12<br>Table S7
Study characteristics	18	For each study, present characteristics for which data were extracted (e.g., study size, PICOS, follow-up period) and provide the citations.	Page 9<br>Page 12<br>Table S7
Risk of bias within studies	19	Present data on risk of bias of each study and, if available, any outcome level assessment.	Page 13<br>Table S9-S10<br>Figure S12-S15

Results of individual studies	20	For all outcomes considered (benefits or harms), present, for each study: 1) simple summary data for each intervention group, and 2) effect estimates and confidence intervals. *Modified approaches may be needed to deal with information from larger networks.*	Page 10-13<br>Figure 3<br>Figure 6
Synthesis of results	21	Present results of each meta-analysis done, including confidence/credible intervals. *In larger networks, authors may focus on comparisons versus a particular comparator (e.g. placebo or standard care), with full findings presented in an appendix. League tables and forest plots may be considered to summarize pairwise comparisons.* If additional summary measures were explored (such as treatment rankings), these should also be presented.	Page 10-13<br>Figure 3-6<br>Figure S1-S4, S6-S9
Exploration for inconsistency	S5	Describe results from investigations of inconsistency. This may include such information as measures of model fit to compare consistency and inconsistency models, *P* values from statistical tests, or summary of inconsistency estimates from different parts of the treatment network.	Page 13<br>Table S10<br>Figure S11
Risk of bias across studies	22	Present results of any assessment of risk of bias across studies for the evidence base being studied.	Page 13<br>Table S11<br>Figure S12-S15
Results of additional analyses	23	Give results of additional analyses, if done (e.g., sensitivity or subgroup analyses, meta-regression analyses, *alternative network geometries studied, alternative choice of prior distributions for Bayesian analyses,* and so forth).	Page 13<br>Table S9<br>Figure S10<br>Table S10<br>Figure S11
**DISCUSSION**			
Summary of evidence	24	Summarize the main findings, including the strength of evidence for each main outcome; consider their relevance to key groups (e.g., healthcare providers, users, and policy-makers).	Page 14
Limitations	25	Discuss limitations at study and outcome level (e.g., risk of bias), and at review level (e.g., incomplete retrieval of identified research, reporting bias). *Comment on the validity of the assumptions, such as transitivity and consistency. Comment on any concerns regarding network geometry (e.g., avoidance of certain comparisons).*	Page 17-18
Conclusions	26	Provide a general interpretation of the results in the context of other evidence, and implications for future	Page 18

		research.	
**FUNDING**			
Funding	27	Describe sources of funding for the systematic review and other support (e.g., supply of data); role of funders for the systematic review. This should also include information regarding whether funding has been received from manufacturers of treatments in the network and/or whether some of the authors are content experts with professional conflicts of interest that could affect use of treatments in the network.	Page 18

PICOS = population, intervention, comparators, outcomes, study design.

* Text in italics indicate wording specific to reporting of network meta-analyses that has been added to guidance from the PRISMA statement.

† Authors may wish to plan for use of appendices to present all relevant information in full detail for items in this section.

Table S2. Literature search strategy.

ID	Search	His
**PubMed**		
#1	"Colorectal Neoplasms"[MeSH Terms] OR "colorectal cancer"[Title/Abstract]	107,573
#2	"intestinal neoplasms"[MeSH Terms] OR "colonic neoplasms"[MeSH Terms] OR "rectal neoplasms"[MeSH Terms]	286,632
#3	"bowel cancer"[Title/Abstract] OR "colon cancer"[Title/Abstract] OR "rectal cancer"[Title/Abstract]	96,194
#4	#1 OR #2 OR #3	316,211
#5	"Proto-Oncogene Proteins B-raf"[MeSH Terms] OR "braf"[All Fields] OR "braf"[Title/Abstract]	26,661
#6	"clinical trial"[Publication Type] OR "randomized controlled trial"[Publication Type] OR "trial"[Title/Abstract] OR "randomized"[Title/Abstract] OR "randomised"[Title/Abstract] OR "randomly"[Title/Abstract] OR (("real-world"[All Fields] AND ("studies"[All Fields] OR "study"[All Fields] OR "study s"[All Fields] OR "studying"[All Fields] OR "studys"[All Fields])) OR "real world study"[Title/Abstract] OR "real world cohort"[Title/Abstract] OR ("real"[All Fields] AND ("world"[All Fields] OR "worlds"[All Fields] OR "worlds"[All Fields]) AND ("cohort"[All Fields] OR "cohort s"[All Fields] OR "cohorte"[All Fields] OR "cohorts"[All Fields])))	2,174,784
#7	0001/01/01:2025/05/31[Date - Publication]	38,941,728
#8	#1 and #2 and #3 and #4	600
#9	#1 and #2 and #3 and #4 Filters: English	591
**Embase**		
#1	'colorectal cancer'/exp OR 'colorectal cancer':ab,ti OR 'intestine cancer'/exp OR 'intestine cancer':ab,ti OR 'colon cancer'/exp OR 'colon cancer':ab,ti OR 'rectal cancer'/exp OR 'rectal cancer':ab,ti	535,197
#2	'B Raf kinase'/exp OR 'BRAF':ab,ti	58,728
#3	'clinical trial'/exp OR 'clinical study'/exp OR 'controlled clinical trial'/exp OR 'randomized controlled trial'/exp OR 'real world study'/exp OR 'real world data'/exp OR 'real world evidence'/exp	14,587,821

	OR 'clinical trial' OR 'real world study' OR 'real world data' OR 'real world evidence' OR 'clinical study' OR 'clinical trial'	
#4	'gene mutation'/exp OR 'mutation':ab,ti	1,303,732
#5	'patient'/exp OR 'patient':ab,ti OR 'patients':ab,ti	14,099,235
#6	'therapy'/exp OR 'treatment'/exp	12,474,171
#7	#1 and #2 and #3 and #4 and #5 and #6 [english]/lim	3,085
#8	#1 and #2 and #3 and #4 and #5 and #6 [english]/lim AND [01-01-0001]/sd NOT [31-05-2025]/sd	2,985
**Cochrane Library**		
#1	'colorectal cancer [MeSH]' OR (colorectal cancer):ab,ti,kw OR (bowel cancer):ab,ti,kw OR (colon cancer):ab,ti,kw OR (rectal cancer):ab,ti,kw	32263
#2	'BRAF [MeSH]' OR (BRAF):ab,ti,kw	2033
#3	'mutation [MeSH]' OR (mutation):ab,ti,kw	15303
#4	#1 and #2 and #3	428
#5	#4 Cochrane Library publication date Between Jan 0001 and May 2025, in Trials	424
**ClinicalTrials.gov**		
#1	"colorectal cancer" OR "colorectal carcinoma" OR "colo-rectal cancer" OR "colorectal cancers" OR "bowel cancer" OR "colon cancer" OR "rectal cancer" | Other terms: BRAF Gene Mutation	134

Table S3. MINORS evaluation Criteria: Risk of bias assessments for the single-arm trials and non-randomised comparative studies.

Item		Score
1	**A clearly stated aim**: the question addressed should be precise and relevant in the light of available literature.	**Scores for each item:**<br>**• 0 (not reported)**<br>**• 1 (reported but inadequate)**<br>**• 2 (reported and adequate)**<br>↓<br>**Scores for overall items (overall risk of bias):**<br>The ideal overall scores are 16 for non-comparative studies and 24 for comparative studies.<br>**• ≥75% (low risk of bias)**<br>**• 50%−75% (medium risk of bias)**<br>**• ≤50% (high risk of bias)**
2	**Inclusion of consecutive patients**: all patients potentially fit for inclusion (satisfying the criteria for inclusion) have been included in the study during the study period (no exclusion or details about the reasons for exclusion).	
3	**Prospective collection of data**: data were collected according to a protocol established before the beginning of the study.	
4	**Endpoints appropriate to the aim of the study**: unambiguous explanation of the criteria used to evaluate the main outcome which should be in accordance with the question addressed by the study. Also, the endpoints should be assessed on an intention-to-treat basis.	
5	**Unbiased assessment of the study endpoint**: blind evaluation of objective endpoints and double-blind evaluation of subjective endpoints. Otherwise, the reasons for not blinding should be stated.	
6	**Follow-up period appropriate to the aim of the study**: the follow-up should be sufficiently long to allow the assessment of the main endpoint and possible adverse events.	
7	**Loss to follow up less than 5%**: all patients should be included in the follow up. Otherwise, the proportion lost to follow up should not exceed the proportion experiencing the major endpoint.	
8	**Prospective calculation of the study size**: information of the size of detectable difference of interest with a calculation of 95% confidence interval, according to the expected incidence of the outcome event, and information about the level for statistical significance and estimates of power when comparing the outcomes.	
9*	**An adequate control group**: having a gold standard diagnostic test or therapeutic intervention recognized as the optimal intervention	

	according to the available published data.	
10*	**Contemporary groups**: control and studied group should be managed during the same time period (no historical comparison).	
11*	**Baseline equivalence of groups**: the groups should be similar regarding the criteria other than the studied endpoints. Absence of confounding factors that could bias the interpretation of the results.	
12*	**Adequate statistical analyses**: whether the statistics were in accordance with the type of study with calculation of confidence intervals or relative risk.	

*For comparative studies only.

MINORS, Methodological index for non-randomised studies.

Table S4. Cochrane Risk of Bias tool version 2 (RoB 2.0) for randomised controlled trials.

Domain	Signalling	
Randomisation process	1.1 Was the allocation sequence random?	Y: Yes<br>PY: Possible Yes<br>N: No<br>PN: Possible No<br>NI: No information
	1.2 Was the allocation sequence concealed until participants were enrolled and assigned to interventions?	
	1.3 Did baseline differences between intervention groups suggest a problem with the randomization process?	
Deviations from intended interventions	2.1 Were participants aware of their assigned intervention during the trial?	
	2.2 Were carers and people delivering the interventions aware of participants' assigned intervention during the trial?	
	2.3 If Y/PY/NI to 2.1 or 2.2: Were there deviations from the intended intervention that arose because of the trial context?	
	2.4 If Y/PY to 2.3: Were these deviations likely to have affected the outcome?	
	2.5. If Y/PY/NI to 2.4: Were these deviations from intended intervention balanced between groups?	
	2.6 Was an appropriate analysis used to estimate the effect of assignment to intervention?	
	2.7 If N/PN/NI to 2.6: Was there potential for a substantial impact (on the result) of the failure to analyse participants in the group to which they were randomized?	
Missing outcome data	3.1 Were data for this outcome available for all, or nearly all, participants randomized?	
	3.2 If N/PN/NI to 3.1: Is there evidence that the result was not biased by missing outcome data?	
	3.3 If N/PN to 3.2: Could missingness in the outcome depend on its true value?	
	3.4 If Y/PY/NI to 3.3: Is it likely that missingness in the outcome depended on its true value?	
Measurement of the outcome	4.1 Was the method of measuring the outcome inappropriate?	
	4.2 Could measurement or ascertainment of	

	the outcome have differed between intervention groups?	
	4.3 If N/PN/NI to 4.1 and 4.2: Were outcome assessors aware of the intervention received by study participants?	
	4.4 If Y/PY/NI to 4.3: Could assessment of the outcome have been influenced by knowledge of intervention received?	
	4.5 If Y/PY/NI to 4.4: Is it likely that assessment of the outcome was influenced by knowledge of intervention received?	
Selection of the reported result	5.1 Were the data that produced this result analysed in accordance with a pre-specified analysis plan that was finalized before unblinded outcome data were available for analysis?	
	5.2 ... multiple eligible outcome measurements (e.g. scales, definitions, time points) within the outcome domain?	
	5.3 ... multiple eligible analyses of the data?	

Table S5. ArRoWS evaluation criteria: risk of bias assessments for the real-world study.

Items		Rating evaluation
**CORE ITEMS**		
1	Is the research question or objective clear?	Good/Moderate/Poor/Unclear
2	Is the study representative of its target population?	Good/Moderate/Poor/Unclear
3	Has a sample size, power calculation or measure of uncertainty (e.g. confidence intervals, standard errors) been provided?	Good/Moderate/Poor/Unclear
4	Are the exposure measures clearly defined and appropriate?	Good/Moderate/Poor/Unclear
5	Is/are the outcome(s) clearly defined and appropriate?	Good/Moderate/Poor/Unclear
6	Are confounders clearly defined and appropriate?	Good/Moderate/Poor/Unclear
7	Are the statistical analyses clearly defined and appropriate?	Good/Moderate/Poor/Unclear
8	Are the limitations of the study defined and appropriate?	Good/Moderate/Poor/Unclear
9	Have the authors drawn appropriate conclusions from their results?	Good/Moderate/Poor/Unclear
**COHORT STUDIES**		
10	Are the methods of follow up defined and appropriate?	Good/Moderate/Poor/Unclear
11	Is the length of follow up sufficient to ascertain outcomes?	Good/Moderate/Poor/Unclear
12	If the authors are measuring treatment effects, is the analysis appropriate (e.g. matching, propensity scoring, instrumental variables)	Good/Moderate/Poor/Unclear
**CASE-CONTROL STUDIES AND COMPARATIVE EFFECTIVENESS STUDIES**		
13	Have the authors explained their choice of cases and controls and matching criteria?	Good/Moderate/Poor/Unclear
14	If a matched case-control study, have the authors described their matching criteria?	Good/Moderate/Poor/Unclear
15	If a matched case-control study, was matching taken into account in the analysis?	Good/Moderate/Poor/Unclear
**ELECTRONIC DATABASE STUDIES**		
16	Have the authors listed/referenced (from previous literature) a code set for relevant tests, procedures, treatments and clinical events (e.g. ICD codes, Read codes)?	Good/Moderate/Poor/Unclear

Table S6: ROBINS-I evaluation criteria: risk of bias assessments for the real-world study.

Signalling questions	Response
**Domain 1: Risk of bias due to confounding Variant A**	NA / Y / PY / PN / N / NI
1.1 Did the authors control for all the important confounding factors for which this was necessary?	
1.2 If Y/PY/WN to 1.1: Were confounding factors that were controlled for (and for which control was necessary) measured validly and reliably by the variables available in this study?	
1.3 If Y/PY/WN to 1.1: Did the authors control for any post-intervention variables that could have been affected by the intervention?	
1.4. Did the use of negative controls, quantitative bias analysis, or other considerations, suggest serious unmeasured confounding?	
Risk of bias judgement	
Optional: What is the predicted direction of bias due to confounding?	
**Domain 1: Risk of bias due to confounding Variant B**	
1.1 Did the authors use an analysis method that was appropriate to control for time-varying as well as baseline confounding?	
1.2 If Y/PY to 1.1: Did the authors control for all the important baseline and time-varying confounding factors for which this was necessary?	
1.3 If Y/PY/WN to 1.2: Were confounding factors that were controlled for (and for which control was necessary) measured validly and reliably by the variables available in this study?	
1.4 If N/PN/NI to 1.1: Did the authors control for time-varying factors or other variables measured after the start of intervention?	
1.5 Did the use of negative controls, or other considerations, suggest serious unmeasured confounding?	
**Domain 2: Risk of bias in classification of interventions**	
2.1 Did assignment of participants to the intervention group or the comparator group rely on events or measurements that occurred after the start of follow up?	
2.2 If Y/PY to 2.1: Were participants included in the comparator group until they fulfilled the definition of the intervention (or vice versa)?	
2.3 If N/PN to 2.1: Was all information used to classify intervention and comparator groups recorded at or before the time the interventions started?	
2.4 Was classification of intervention status influenced by knowledge of the outcome or risk of the outcome?	
2.5 If N/PN to 2.1 and WY/N/PN/NI 2.4: Was intervention status classified correctly for all, or nearly all, participants?	
**Domain 3: Risk of bias in selection of participants into the study (or into the analysis)**	
3.1 (=2.1) Did assignment of participants to the intervention group or the comparator group rely on events or measurements that occurred after the start of follow up?	
3.2 If Y/PY to 3.1: Were participants excluded after the start of follow-up because they did not meet the definition of either the intervention or the comparator?	

3.3 Were start of follow up and start of intervention the same for most participants?	
3.4 If N/PN to 3.3: Is the effect of intervention expected to be constant over the time period studied?	
3.5 Was selection of participants into the study (or into the analysis) based on participant characteristics observed after the start of intervention (additional to the situations addressed in 3.1 and 3.3)?	
3.6 If Y/PY to 3.5: Were the post-intervention variables that influenced selection likely to be associated with intervention?	
3.7 If Y/PY to 3.6: Were the post-intervention variables that influenced selection likely to be influenced by the outcome or a cause of the outcome?	
3.8 If Y/PY to 3.2, N/PN 3.4 or Y/PY to 3.7: Is it likely that the analysis corrected for all of the potential selection biases identified in 3.1-3.2, 3.3-3.4 or 3.5-3.7 above?	
3.9 If N/PN to 3.8: Did sensitivity analyses demonstrate that the likely impact of the potential selection biases identified in 3.1-3.2, 3.3-3.4 or 3.5-3.7 above was minimal?	
3.10 If N/PN to 3.9: Were potential selection biases identified in 3.1-3.2, 3.3-3.4 or 3.5-3.7 above sufficiently severe that the result should not be included in a quantitative synthesis?	
**Domain 4: Risk of bias due to deviations from intended interventions Variant A**	
4.1 Was the study undertaken in an experimental context?	
4.2. If Y/PY to 4.1: Did participants deviate from the intended intervention as a result of the processes of recruiting and engaging them in the study?	
4.3. If Y/PY to 4.1: Did study personnel consciously or unconsciously undermine implementation of the intended interventions?	
4.4. If Y/PY/NI to 4.2 or 4.3: Were these deviations from intended intervention likely to have affected the outcome?	
4.5. Was an appropriate analysis used to estimate the effect of assignment to intervention?	
**Domain 4: Risk of bias due to deviations from intended interventions Variant B**	
4.1 Did all or nearly all participants adhere to their assigned intervention strategy?	
4.2. If N/PN/NI to 4.1: Were the protocol deviations likely to have affected the outcome?	
4.3 If Y/PY to 4.2: Was an appropriate analysis used to estimate the specified per-protocol effect, accounting for the specified protocol deviations?	
**Domain 5: Risk of bias due to missing data**	
5.1 Were complete data on intervention status available for all, or nearly all, participants?	
5.2 Were complete data on the outcome available for all, or nearly all, participants?	
5.3 Were complete data on important confounding variables available for all, or nearly all, participants?	
5.4 If N/PN/NI to 5.1, 5.2 or 5.3: Is the result based on a complete case analysis?	

5.5 If Y/PY/NI to 5.4: Was exclusion from the analysis because of missing data (in intervention, confounders or the outcome) likely to be related to the true value of the outcome?	
5.6 If Y/PY/NI to 5.5: Is the relationship between the outcome and missingness likely to be explained by the variables in the analysis model?	
5.7 If N/PN to 5.4: Was the analysis based on imputing missing values?	
5.8 If Y/PY to 5.7: Is it reasonable to assume that data were 'missing at random' (MAR) or 'missing completely at random' (MCAR)?	
5.9 If Y/PY to 5.8: Was imputation performed appropriately?	
5.10 If N/PN/NI to 5.7: Was an appropriate alternative method used to correct for bias due to missing data?	
5.11 If PN/N/NI to 5.1, 5.2 or 5.3 AND (Y/PY/NI to 5.5 OR (Y/PY to 5.8 AND WN/SN/NI to 5.9) OR WN/SN/NI to 5.10): Is there evidence that the result was not biased by missing data?	
**Domain 6: Risk of bias arising from measurement of the outcome**	
6.1 Could measurement or ascertainment of the outcome have differed between intervention groups?	
6.2 Were outcome assessors aware of the intervention received by study participants?	
6.3 If Y/PY/NI to 6.2: Could assessment of the outcome have been influenced by knowledge of the intervention received?	
**Domain 7: Risk of bias in selection of the reported result**	
7.1 Was the result reported in accordance with an available, pre-determined analysis plan?	
Is the numerical result being assessed likely to have been selected, on the basis of the results, from...	
7.2 ... multiple outcome measurements (e.g. scales, definitions, time points) within the outcome domain?	
7.3 ... multiple analyses of the data?	
7.4 ... multiple subgroups?	

Table S7. Baseline characteristics of the included studies.

First Author, Year	Trial Name/Registered Number	Country	Study Design	No of participants /Mutation type	Treatment Strategy	Treatment Line	Data Source	Reported outcome				
								HR for OS	HR for PFS	ORR	DCR	Grade 3 or higher AEs
**Single-Arm Trial**												
Loupakis F, et al 2014	NCT01437618	Italy	Phase II trial	15 BRAF V600E	TCT-anti-VEGF	First-line setting	Trial-level data			✓	✓	
Wang Z, et al 2022	IMPROVEMENT/ NCT03727763	China	Phase II trial	12 BRAF V600E	DCT-anti-EGFR/BRAF	First-line setting	Trial-level data			✓	✓	
Masi G, et al 2010	NCT01163396	Italy	Phase II trial	10 BRAF V600E	TCT-anti-VEGF	First-line setting	Subgroup-level data			✓		
Van Cutsem E, et al 2023	ANCHOR CRC/ NCT03693170	Multinational	Phase II trial	95 BRAF V600E	Anti-EGFR/BRAF /MEK	First-line setting	Trial-level data			✓	✓	
Tabernero J, et al 2024	BREAKWATER safety lead-in/ NCT04607421	Multinational	Phase I trial	12 BRAF V600E	DCT-anti-EGFR/BRAF	First-line setting	Trial-level data			✓		
Kasper S, et al 2020	CEBIFOX/ NCT01051167	Germany	Phase II trial	5 BRAF V600E	DCT-anti-EGFR	First-line setting	Subgroup-level data			✓	✓	
Modest DP, et al 2012	AIO KRK-0104-trial/ NCT00254137)	Germany	Phase II trial	17 BRAF V600E	DCT-anti-EGFR	First-line setting	Subgroup-level data			✓	✓	

Kaczirek K, et al 2015	CECOG/CORE-1.2.002/ NCT00479752	Germany	Phase II trial	14 BRAF V600E	DCT-anti-EGFR	First-line setting	Subgroup-level data			✓	✓	
Tian J, et al 2023	NCT03668431	USA	Phase II trial	37 BRAF V600E	Anti-PD-1/BRAF/MEK	First-/Second-/later-line setting*	Trial-level data			✓	✓	
Qiu M, et al 2023	NCT05019534	China	Phase I	12 BRAF V600E	Anti-PD-1/EGFR/BRAF	Second- or later-line setting	Trial-level data			✓	✓	✓
Shi Y, et al 2024	NCT03781219	China	Phase I trial	25 BRAF V600E	Anti-BRAF/MEK	Later-line setting	Subgroup-level data			✓	✓	
Klute KA, et al 2022	TAPUR/ NCT02693535	USA	Phase II	30 BRAF V660E (N=29) BRAF K601E (N=1)	Anti-BRAF/MEK	Second- or later-line setting	Trial-level data			✓	✓	✓
Corcoran RB, et al 2015	NCT01072175	USA	Phase I/II	43 BRAF V600E	Anti-BRAF/MEK	First-/Second-/later-line setting*	Trial-level data			✓	✓	✓
Morris VK, et al 2022	NCT04017650	USA	Phase I/II	26 BRAF V600E	Anti-PD-1/EGFR/BRAF	Second- or later-line setting	Trial-level data			✓	✓	✓

Yaeger R, et al 2015	/	USA	/	15<br>BRAF V600E	Anti-EGFR/BRAF	Second-/later-line setting	Trial-level data			✓	✓	
Hyman DM, et al 2015	NCT01524978	Multinational	Phase II	27<br>BRAF V600E/X	Anti-EGFR/BRAF	Second- or later-line setting	Trial-level data			✓	✓	
Tan L, et al 2023	EViCT/<br>ACTRN12614000486628	Australia	Phase I/II	32<br>BRAF V600E	Anti-EGFR/BRAF	Second- or later-line setting	Trial-level data			✓	✓	
Parikh AR, et al 2024	HERKULES-3/<br>NCT05039177	USA	Phase Ib/II	29<br>BRAF V600E	Anti-EGFR/BRAF	Second- or later-line setting	Trial-level data			✓	✓	✓
Chen Y, et al 2024	ChiCTR2200063316	China	Phase Ib/II	10<br>BRAF V600E	SCT-anti-EGFR/BRAF/MEK	Later-line setting	Trial-level data			✓	✓	
Kopetz S, et al 2015	NCT00405587	USA	Phase II	21<br>BRAF V600E	Anti-BRAF	First-/Second-/later-line setting*	Trial-level data			✓	✓	✓
Falchook GS, et al 2012	NCT00880321	Multinational	Phase I	11<br>9-BRAF V600E (n=9)<br>Other (n=2)	Anti-BRAF	First-/Second-/later-line setting*	Subgroup-level data			✓	✓	
Kotani D, et al 2024	EAP/<br>Japic- CTI number, 205146	Japan	/	81<br>BRAF V600E	Anti-EGFR/BRAF/MEK	Second-/later-line setting	Trial-level data			✓	✓	✓

Van Cutsem E, et al 2019	BEACON SLI/ NCT02928224	Multinational	/	30 BRAF V600E	Anti-EGFR/BRAF/MEK	Second-/later-line setting	Trial-level data			✓	✓	✓
Tabernero J, et al 2023	NCT02278133	Multinational	Phase Ib/II	20 BRAF V600E	Anti-EGFR/BRAF/WNT	Second-/later-line setting	Trial-level data			✓	✓	✓
Hong DS, et al 2016	NCT01787500	USA	Phase Ib	19 BRAF V600E	SCT-anti-EGFR/BRAF	Second-/later-line setting	Trial-level data			✓	✓	
Wang Z, et al 2022	IMPROVEMENT/ NCT03727763	China	Phase II trial	9 BRAF V600E	DCT-anti-EGFR/BRAF	Second-/later-line setting	Trial-level data			✓	✓	
Tabernero J, et al 2024	BREAKWATER safety lead-in/ NCT04607421	Multinational	Phase I trial	18 BRAF V600E	DCT-anti-EGFR/BRAF	Second-/later-line setting	Trial-level data			✓		
**Controlled Clinical Trial**													
Schmoll HJ, et al 2024	CHARTA/ NCT01321957	Germany	Phase II RCT	61 vs 54 BRAF V600E	TCT-anti-VEGF vs DCT-anti-VEGF	First-line setting	Subgroup-level data	✓	✓	✓		
Gruenberger T, et al 2015	OLIVIA/ NCT00778102	Multinational	Phase II RCT									
Hurwitz HI, et al 2019	STEAM/ NCT01765582	USA	Phase II RCT									
Cremolini C, et al 2015	TRIBE/ NCT00719797	Italy	Phase III RCT									

Cremolini C, et al 2020	TRIBE2/ NCT02339116	Italy	Phase III RCT									
Bond MJG, et al 2023	CAIRO5/ NCT02162563	Multinational	Phase III RCT	12 vs 10 BRAF V600E	TCT-anti-VEGF vs DCT-anti-VEGF	First-line setting	Subgroup-level data	✓	✓			
Maughan TS, et al 2011	COIN/ ISRCTN27286448	Multinational	Phase III RCT	45 vs 57 BRAF V600E (N=90) BRAF D549G (N=12)	DCT-anti-EGFR vs DCT	First-line setting	Subgroup-level data	✓	✓			
Innocenti F, et al 2019	CALGB80405/ NCT00265850	Multinational	Phase III RCT	26 vs 41 vs 33 BRAF V600E (N=98) Other (N=2)	DCT-anti-EGFR/VEGF vs DCT-anti-VEGF vs DCT-EGFR	First-line setting	Subgroup-level data	✓	✓			
Van Cutsem E, et al 2011	CRYSTAL/ NCT00154102	Multinational	Phase III RCT	32 vs 38 BRAF V600E	DCT-anti-EGFE vs DCT	First-line setting	Subgroup-level data	✓	✓	✓		
Bokemeyer C, et al 2011	OPUS/ NCT00125034	Multinational	Phase II RCT									
Douillard JY, et al 2013	PRIME/ NCT00364013	Multinational	Phase III RCT	24 vs 29 BRAF V600E	DCT-anti-EGFR vs DCT	First-line setting	Subgroup-level data	✓	✓			
Rivera F, et al 2017	PEAK/ NCT00819780	Multinational	Phase II RCT	11 vs 3 BRAF V600E	DCT-anti-EGFR vs DCT-anti-VEGF	First-line setting	Subgroup-level data	✓	✓	✓		

Stintzing S, et al 2023	FIRE-4.5/ NCT04034459	Germany	Phase II RCT	72 vs 35 BRAF V600E	TCT-anti-EGFR vs TCT-anti-VEGF	First-line setting	Trial-level data	✓	✓	✓	✓	
Ten Hoorn S, et al 2023	CAIRO2/ NCT00208546	Netherlands	Phase III RCT	30 vs 27 BRAF V600E	DCT-anti-EGFR/VEGF vs DCT-anti-VEGF	First-line setting	Subgroup-level data	✓				
Elez E, et al 2025	BREAKWATER/ NCT04607421	Multinational	Phase III RCT	158 vs 236 vs 243 BRAF V600E	Anti-EGFR/BRAF vs DCT-anti-EGFR/BRAF vs DCT-anti-VEGF§	First-line setting	Trial-level data	✓	✓	✓	✓	✓
Stintzing S, et al 2017	FIRE-3/ NCT00433927	Germany	Phase III RCT	23 vs 25 BRAF V600E BRAF G466V	DCT-anti-EGFR vs DCT-anti-VEGF	First-line setting	Subgroup-level data	✓	✓	✓	✓	
Tol J, et al 2009	NCT00208546	Netherlands	Phase III RCT	28 vs 17 BRAF V600E	DCT-anti-EGFR/VEGF vs DCT-anti-VEGF	First-line setting	Subgroup-level data			✓		
Tveit KM, et al 2012	NORDIC-VII/ NCT00145314	Norway	Phase III RCT	36 vs 19 BRAF V600E	DCT-anti-EGFR vs DCT	First-line setting	Subgroup-level data			✓		
Aranda E, et al 2020	VISNÚ-1/ NCT01640405	Spain	Phase III RCT	16 vs 17 BRAF V600E	TCT-anti-VEGF vs DCT-anti-VEGF	First-line setting	Subgroup-level data		✓			
Lopez-Crapez E, et al 2018	METHEP2/ NCT01442935	France	Phase II RCT	3 vs 5 vs 1 BRAF V600E	DCT-anti-EGFR vs TCT-anti-EGFR vs DCT-anti-VEGF	First-line setting	Subgroup-level data			✓		

Chen Y, et al 2024	NCT05540951	China	Phase III NRCT	38 vs 16 vs 24 BRAF V600E	SCT-anti-EGFR/BRAF vs TCT-anti-VEGF vs DCT-anti-VEGF	First-line setting	Trial-level data	✓	✓	✓		✓
Tabernero J, et al 2021	BEACON/ NCT02928224	Multinational	Phase III RCT	224 vs 220 vs 221 BRAF V600E	Anti-EGFR/BRAF/MEK vs Anti-EGFR/BRAF vs Chemo-anti-EGFR	Second- and later-line setting	Trial-level data	✓	✓	✓	✓	✓
Tabernero J, et al 2016	NCT01719380	Multinational	Phase Ib NRCT	52 vs 50 BRAF V660E	Anti-EGFR/BRAF/PI3K vs Anti-EGFR/BRAF	Second- and later-line setting	Trial-level data	✓	✓	✓		✓
Corcoran RB, et al 2018	NCT01750918	Multinational	Phase I NRCT	91 vs 31 vs 20 BRAF V600E	Anti-EGFR/BRAF/MEK vs Anti-EGFR/MEK vs Anti-EGFR/BRAF	First-/Second-/later-line setting*	Trial-level data	✓	✓	✓	✓	✓
Seymour MT, et al 2013	PICCOLO/ ISRCTN93248876	UK	Phase III RCT	37 vs 31 BRAF V600E	Chemo-anti-EGFR vs Chemo	Second-/later-line setting	Subgroup-level data	✓	✓	✓		
Peeters M, et al 2015	20050181/ NCT0039183	Multinational	Phase III RCT	22 vs 23 BRAF V600E	Chemo-anti-EGFR vs Chemo	Second-/later-line setting	Subgroup-level data	✓	✓			

Kopetz S, et al 2021	SWOG S1406/ NCT02164916	USA	Phase II RCT	50 vs 50 BRAF V600E	Chem-anti-EGFR/BRAF vs Chem-EGFR	Second-/later-line setting	Trial-level data	✓	✓	✓	✓	✓
Wang X, et al 2024	NAUTICAL CRC/ NCT05004350	China	Phase II RCT	65 vs 32 BRAF V600E	Anti-EGFR/BRAF vs Chemo-anti-EGFR	Second-/later-line setting	Trial-level data	✓	✓	✓		✓
**Real-World Study**												
Trunk A, et al 2022	/	USA	RWS	302 vs 121 BRAF V600E (N=311) Other (N=112)	DCT-anti-VEGF vs DCT	First-line setting	Trial-level data	✓				
Shimozaki K, et al 2022	BRACELET	Japan	RWAS	79 vs 91 BRAF V600E	TCT-anti-VEGF vs DCT-anti-VEGF	First-line setting	Trial-level data	✓	✓	✓	✓	✓
Xu Y, et al 2023	/	China	RWS	33 vs 55 vs 54 BRAF V600E	SCT-anti-EGFR/BRAF vs DCT-anti-VEGF vs DCT	First-line setting	Trial-level data	✓	✓	✓	✓	
Stintzing S, et al 2024	BERING-CRC/ NCT04673955	Germany	/	95 BRAF V600E	Anti-EGFR/BRAF	Second-/later-line setting	Trial-level data			✓		✓
Fedyanin M, et al 2021	/	Russia	RWS	30 BRAF V600E	Anti-EGFR/BRAF	Second- and later-line setting	Trial-level data			✓	✓	
Gallois C, et al 2024	AGEO	Multinational	RWS	21 vs 180[†] BRAF V600E	Anti-EGFR/BRAF/MEK	First-/Second-	Trial-level data	✓	✓	✓	✓	✓

					vs Anti-EGFR/BRA	/later-line setting*						
Boccaccino A, et al 2022	/	Italy	RWS	36 vs 97 BRAF V600E	Anti-EGFR/BRAF/MEK vs Anti-EGFR/BRAF	First-/Second-/later-line setting*	Trial-level data	✓	✓	✓	✓	✓

* In a few second- or later-line studies, treatment-naïve patients were enrolled, but the enrollment proportion of such patients was less than 5% in all cases; § Control group receiving multiple therapeutic regimens were classified according to the most frequently administered strategy; † ORR and DCR data were unavailable for a subset of patients.

First-line regimen: Doublet chemotherapy plus EGFR inhibitor, DCT-anti-EGFR; Triplet chemotherapy plus EGFR inhibitor, TCT-anti-EGFR; Doublet chemotherapy plus VEGF inhibitor, DCT-anti-VEGF; Triplet chemotherapy plus VEGF inhibitor, TCT-anti-VEGF; Doublet chemotherapy plus EGFR inhibitor and VEGF inhibitor, DCT-anti-EGFR/VEGF; EGFR/BRAF inhibitors, anti-EGFR/BRAF; Single-agent chemotherapy plus EGFR inhibitor and BRAF inhibitor, SCT-anti-EGFR/BRAF; Doublet chemotherapy plus EGFR inhibitor and BRAF inhibitor, DCT-anti-EGFR/BRAF; EGFR inhibitor combined with BRAF and MEK inhibitors, anti-EGFR/BRAF/MEK;

Second- or later-line regimen: Chemotherapy plus EGFR inhibitor, chemo-anti-EGFR; Chemotherapy plus EGFR/BRAF inhibitor, chemo-anti-EGFR/BRAF; Chemotherapy plus EGFR/BRAF/MEK inhibitors, chemo-anti-EGFR/BRAF/MEK; BRAF inhibitor, anti-BRAF; EGFR/MEK inhibitors, anti-EGFR/MEK; EGFR/BRAF inhibitors, anti-EGFR/BRAF; EGFR/BRAF/MEK inhibitors, anti-EGFR/BRAF/MEK; EGFR/BRAF/PI3K inhibitors, anti-EGFR/BRAF/PI3K; EGFR/BRAF/ERK inhibitors, anti-EGFR/BRAF/ERK; EGFR/BRAF/WNT inhibitor, anti-EGFR/BRAF/WNT; EGFR/BRAF/PD-1 inhibitors, anti-EGFR/BRAF/PD-1; BRAF/MEK/PD-1 inhibitors, anti-BRAF/MEK/PD-1.

Table S8: Categorization of treatment regimens for first-line, second-line, and later-line therapy.

Chemotherapy category	1. Single-agent chemotherapy (SCT);<br>2. Doublet chemotherapy (DCT);<br>3. Triplet chemotherapy (TCT);
Targeted therapy category	1. EGFR inhibitor (anti-EGFR);<br>2. VEGF inhibitor (anti-VEGF);<br>3. BRAF inhibitor (anti-BRAF);<br>4. MEK inhibitor (anti-MEK);<br>5. PI3K inhibitor (anti-PI3K);<br>6. ERK inhibitor (anti-ERK);<br>7. WNT inhibitor (anti-WNT)<br>8. PD-1 inhibitor (anti-PD-1)
Combination strategies in first-line setting	1. EGFR inhibitor-based combinations including:<br>Doublet chemotherapy-anti-EGFR (DCT-anti-EGFR);<br>Triplet chemotherapy-anti-EGFR (TCT-anti-EGFR);<br>2. VEGF inhibitor-based combinations including:<br>Doublet chemotherapy-anti-VEGF (DCT-anti-VEGF;<br>Triplet chemotherapy-anti-VEGF (TCT-anti-VEGF);<br>3. EGFR/VEGF inhibitors-based combinations including:<br>Doublet chemotherapy-anti-EGFR/VEGF(DCT-anti-EGFR/VEGF)<br>4. EGFR/BRAF inhibitor-based combinations including:<br>Single-agent chemotherapy-anti-EGFR/BRAF (SCT-anti-EGFR/BRAF)<br>Doublet chemotherapy-anti-EGFR/BRAF (DCT-anti-EGFR/BRAF);<br>5.Targeted therapy alone regimens including:<br>Anti-EGFR/BRAF<br>Anti-EGFR/BRAF/MEK
Combination strategies in second- or later-line setting	1. Chemotherapy plus EGFR inhibitor (Chemotherapy-anti-EGFR);<br>2. Chemotherapy plus EGFR/BRAF inhibitor (chemotherapy-anti-EGFR/BRAF)<br>3. Chemotherapy plus EGFR/BRAF/MEK inhibitors (chemotherapy-anti-EGFR/BRAF/MEK),<br>4. EGFR/MEK inhibitors (anti-EGFR/MEK),<br>5. EGFR/BRAF inhibitors (anti-EGFR/BRAF),<br>6. BRAF/MEK inhibitors (anti-BRAF/MEK),<br>7. EGFR/BRAF/MEK inhibitors (anti-EGFR/BRAF/MEK),<br>8. EGFR/BRAF/PI3K inhibitors (anti-EGFR/BRAF/PI3K),<br>9. EGFR/BRAF/ERK inhibitors (anti-EGFR/BRAF/ERK),<br>10. EGFR/BRAF/WNT inhibitor (anti-EGFR/BRAF/WNT),<br>11. EGFR/BRAF/PD-1 inhibitors (anti-EGFR/BRAF/PD-1),<br>12.BRAF/MEK/PD-1 inhibitors (anti-BRAF/MEK/PD-1).

Table S9. Results of network meta-regression analysis assessing potential effect modifiers.

Outcome	Number of included studies	**Sample size** (Number of patients)	**Study design** (Clinical trial vs RWS)	**Data source** (Trial-level data vs Subgroup-level data)	**BRAF mutation type** (BRAF V600E vs Mixed type)	**Prior treatment history** (Uniform-exposure group vs Mixed-exposure group)	**Publication type (**Published literature vs Conference report**)**
		Beta-coefficient with 95%CI	Beta-coefficient with 95%CI	Beta-coefficient with 95%CI	Beta-coefficient with 95%CI	Beta-coefficient with 95%CI	Beta-coefficient with 95%CI
First-line setting							
Overall survival	20	(N=2138) 0.06 (-0.55 to 0.66)	(17 vs 3) 0.06 (-0.49 to 0.62)	(6 vs 14) 0.05 (-0.51 to 0.62)	(16 vs 4)) -0.24 (-0.69 to 0.20)	(20 vs 0) /	(20 vs 0) /
Progression-free survival	19	(N=1691) 1.02 (-1.32 to 4.52)	(17 vs 2) 0.04 (-0.70 to 0.75)	(5 vs 14) 0.05 (-0.68 to 0.74)	(16 vs 3) -0.44 (-1.04 to 0.18)	(19 vs 0) /	(19 vs 0) /
Objective response rate	17	(N=1490) 2.38 (-4.42 to 10.9)	(15 vs 2) 10.90 (-1.64-3.28)	(5 vs 12) 0.97 (-1.44-3.34)	(16 vs 1) -1.98 (-21.4 to 456)	(17 vs 0) /	(16 vs 1) -1.60 (-43.2 to 21.4)
Disease control rate	5	(N=1104) -0.21(-18.3 to 20.3)	(3 vs 2) -1.51 (-26.5 to 13.0)	(4 vs 1) -2.75 (-56.4-35.4)	(4 vs 1) 0.06 (-50.7 to 21.6)	(5 vs 0) /	(5 vs 0) /
Adverse events	3	(N=862) 0.48 (-6.12 to 9.02)	(2 vs 1) -5.46 (-32.2 to 8.00)	(3 vs 0) /	(3 vs 0) /	(3 vs 0) /	(3 vs 0) /
Second- or later-line setting							
Overall survival	9	(N=1553) 1.90 (-1.90 to 14.6)	(7 vs 2) -0.38 (-8.86 to 5.77)	(2 vs 7) 0.35 (-4.11 to 8.01)	(9 vs 0) /	(3 vs 6) 0.13 (-4.56 to 5.71)	(7 vs 2) -0.01 (-8.03 to 7.03)

Progression-free survival	9	(N=1553) 0.91 (-3.59-9.16)	(7 vs 2) -0.12 (-14.3-9.49)	(7 vs 2) -0.17 (-7.45 to 5.29)	(9 vs 0) /	(3 vs 6) 0.17 (-5.84 to 7.88)	(7 vs 2) 0.03 (-11.3 to 10.6)
Objective response rate	8	(N=1453) 1.11 (-10.8 to 17.0)	(6 vs 2) 0.84 (-17.5 to 29.0)	(7 vs 1) -0.07 (-11.2 to 8.92)	(8 vs 0) /	(5 vs 3) 4. 78 (-41.6 to 76.8)	(6 vs 2) -0.91 (-38.7 to 24.1)
Disease control rate	4	(N=1075) 0.44 (-4.40 to 6.37)	(2 vs 2) -0.89 (-10.5 to 6.02)	(4 vs 0) /	(4 vs 0) /	(2 vs 2) 0.84 (-12.9 to 14.5)	(4 vs 0) /
Adverse events	7	(N=1448) 0.40 (-1.07 to 1.51)	(5 vs 2) -0.11 (-5.47 to 6.35)	(7 vs 0) /	(7 vs 0) /	(4 vs 3) 0.10 (-6.29 to 7.72)	(5 vs 2) -0.61 (-2.1 to 0.88)

The results were reported as beta coefficients with associated 95% credible intervals (in parentheses), obtained through network meta-regression analysis. Nonsignificant effects were identified when the 95% credible intervals encompassed zero[1].

Definition: Number of included studies: defined as the number of studies included in each network meta-analysis. **Sample size** (Number of patients): defined as the number of patients with advanced BRAFmut colorectal cancer in each network meta-analysis. **Study design** (Clinical trial vs RWS): categorized by study design types (clinical trials versus real-world studies) for each network meta-analysis. **Data source** (Trial-level data vs Subgroup-level data): categorized by data source (trial-level data were from studies prospectively enrolling patients with BRAFmut CRC, subgroup-level data were from studies with post hoc analyses of BRAFmut CRC subsets). **BRAF mutation type** (BRAF V600E vs Mixed type): categorized by BRAF mutation type (BRAF V600E denoted eligible studies including patients harboring exclusively BRAF V600E variant, while mixed type denoted studies including patients with minimal non-V600E mutations). **Prior treatment history** (Uniform-exposure group vs Mixed-exposure group): categorized by prior treatment history (Uniform-exposure group comprised patients with identical prior therapy exposure, contrasting with mixed-exposure group where heterogeneous pretreatment histories existed, e.g. predominantly pretreated population with minimal treatment-naïve individuals). **Publication type** (Published literature vs Conference report): categorized by publication types (Published literature indicated peer-reviewed full-text articles in indexed journals, as opposed to conference reports containing available data from conference abstract or presentations).

Table S10. Comparison of the fit goodness between consistency and inconsistency models based on DIC values in network meta-analysis.

Treatment setting	Outcome	Model	Dbar	pD	DIC
First-line setting	Overall survival	Consistency	16.5	10.1	26.5
		Inconsistency	18.2	14.5	32.7
	Progression-free survival	Consistency	15.4	10.0	25.4
		Inconsistency	16.9	13.5	30.4
	Objective response rate	Consistency	29.9	22.9	52.8
		Inconsistency	24.6	23.8	48.4
	Disease control rate	Consistency	12.2	12.2	24.4
		Inconsistency	12.2	12.2	24.4
	Adverse events	Consistency	7.08	7.08	14.2
		Inconsistency	7.09	7.09	14.2
Second- or later-line setting	Overall survival	Consistency	12.0	8.66	20.7
		Inconsistency	11.7	9.52	21.2
	Progression-free survival	Consistency	12.3	9.22	21.5
		Inconsistency	11.8	9.98	21.8
	Objective response rate	Consistency	16.0	14.8	31.8
		Inconsistency	16.7	16.0	32.7
	Disease control rate	Consistency	9.03	8.52	17.5
		Inconsistency	8.66	8.60	17.3
	Adverse events	Consistency	16.7	14.6	31.3
		Inconsistency	16.5	15.6	32.1

The Deviance information criterion (DIC) quantifies model goodness-of-fit while adjusting for model complexity, with lower values correspond to preferable models[2].

Table 11. CINeMA Evidence Grading Results[3].

Comparison	Number of studies	Within-study bias	Reporting bias	Indirectness	Imprecision	Heterogeneity	Incoherence	Confidence rating
NMA for OS in first-line setting (1=DCT, 2=DCT-anti-EGFR, 3=DCT-anti-VEGF, 4=DCT-anti-EGFR/VEGF, 5=TCT-anti-EGFR, 6=TCT-anti-VEGF, 7=Anti-EGFR/BRAF, 8=SCT-anti-EGFR/BRAF, 9=DCT-anti-EGFR/BRAF)								
Mixed evidence								
1 vs 2	3	No concerns	Low risk	No concerns	Some concerns	No concerns	No concerns	Moderate
1 vs 3	3	No concerns	Low risk	No concerns	Some concerns	No concerns	No concerns	Moderate
1 vs 8	1	No concerns	Low risk	No concerns	No concerns	No concerns	No concerns	High
2 vs 3	3	No concerns	Low risk	No concerns	Major concerns	No concerns	No concerns	Low
2 vs 4	1	No concerns	Low risk	No concerns	Major concerns	No concerns	No concerns	Low
3 vs 4	1	No concerns	Low risk	No concerns	Major concerns	No concerns	No concerns	Low
3 vs 6	3	No concerns	Low risk	No concerns	Some concerns	Some concerns	No concerns	Low
3 vs 7	1	No concerns	Low risk	No concerns	No concerns	No concerns	No concerns	High
3 vs 8	1	No concerns	Low risk	No concerns	No concerns	No concerns	No concerns	High
3 vs 9	1	No concerns	Low risk	No concerns	No concerns	No concerns	No concerns	High
5 vs 6	1	No concerns	Low risk	No concerns	Some concerns	Some concerns	No concerns	Low
7 vs 9	1	No concerns	Low risk	No concerns	No concerns	No concerns	No concerns	High
Indirect evidence								
1 vs 4	0	No concerns	Low risk	No concerns	Major concerns	No concerns	No concerns	Low
1 vs 5	0	No concerns	Low risk	No concerns	Major concerns	No concerns	No concerns	Low
1 vs 6	0	No concerns	Low risk	No concerns	Some concerns	No concerns	No concerns	Moderate
1 vs 7	0	No concerns	Low risk	No concerns	No concerns	No concerns	No concerns	High
1 vs 9	0	No concerns	Low risk	No concerns	No concerns	No concerns	No concerns	High

2 vs 5	0	No concerns	Low risk	No concerns	Major concerns	No concerns	No concerns	Low
2 vs 6	0	No concerns	Low risk	No concerns	Major concerns	No concerns	No concerns	Low
2 vs 7	0	No concerns	Low risk	No concerns	No concerns	Some concerns	No concerns	Moderate
2 vs 8	0	No concerns	Low risk	No concerns	No concerns	No concerns	No concerns	High
2 vs 9	0	No concerns	Low risk	No concerns	No concerns	No concerns	No concerns	High
3 vs 5	0	No concerns	Low risk	No concerns	Major concerns	No concerns	No concerns	Low
4 vs 5	0	No concerns	Low risk	No concerns	Major concerns	No concerns	No concerns	Low
4 vs 6	0	No concerns	Low risk	No concerns	Major concerns	No concerns	No concerns	Low
4 vs 7	0	No concerns	Low risk	No concerns	No concerns	Some concerns	No concerns	Moderate
4 vs 8	0	No concerns	Low risk	No concerns	No concerns	No concerns	No concerns	High
4 vs 9	0	No concerns	Low risk	No concerns	No concerns	No concerns	No concerns	High
5 vs 7	0	No concerns	Low risk	No concerns	No concerns	Some concerns	No concerns	Moderate
5 vs 8	0	No concerns	Low risk	No concerns	No concerns	No concerns	No concerns	High
5 vs 9	0	No concerns	Low risk	No concerns	No concerns	No concerns	No concerns	High
6 vs 7	0	No concerns	Low risk	No concerns	Some concerns	No concerns	No concerns	Moderate
6 vs 8	0	No concerns	Low risk	No concerns	No concerns	Some concerns	No concerns	Moderate
6 vs 9	0	No concerns	Low risk	No concerns	No concerns	No concerns	No concerns	High
7 vs 8	0	No concerns	Low risk	No concerns	Major concerns	No concerns	No concerns	Low
8 vs 9	0	No concerns	Low risk	No concerns	Major concerns	No concerns	No concerns	Low
NMA for PFS in first-line setting **(**1=DCT, 2=DCT-anti-EGFR, 3=DCT-anti-VEGF, 4=DCT-anti-EGFR/VEGF, 5=TCT-anti-EGFR, 6=TCT-anti-VEGF, 7=Anti-EGFR/BRAF, 8=SCT-anti-EGFR/BRAF, 9=DCT-anti-EGFR/BRAF)								
Mixed evidence								
1 vs 2	3	No concerns	Low risk	No concerns	Some concerns	No concerns	No concerns	Moderate
1 vs 3	1	No concerns	Low risk	No concerns	No concerns	No concerns	No concerns	High
1 vs 8	1	No concerns	Low risk	No concerns	No concerns	No concerns	No concerns	High

2 vs 3	3	No concerns	Low risk	No concerns	Some concerns	No concerns	No concerns	Moderate
2 vs 4	1	No concerns	Low risk	No concerns	Major concerns	No concerns	No concerns	Low
3 vs 6	4	No concerns	Low risk	No concerns	Some concerns	No concerns	No concerns	Moderate
3 vs 7	1	No concerns	Low risk	No concerns	Some concerns	No concerns	No concerns	Moderate
3 vs 8	1	No concerns	Low risk	No concerns	No concerns	No concerns	No concerns	High
3 vs 9	1	No concerns	Low risk	No concerns	No concerns	No concerns	No concerns	High
5 vs 6	1	No concerns	Low risk	No concerns	No concerns	No concerns	No concerns	High
7 vs 9	1	No concerns	Low risk	No concerns	No concerns	No concerns	No concerns	High
Indirect evidence								
1 vs 4	0	No concerns	Low risk	No concerns	Major concerns	No concerns	No concerns	Low
1 vs 5	0	No concerns	Low risk	No concerns	Major concerns	No concerns	No concerns	Low
1 vs 6	0	No concerns	Low risk	No concerns	No concerns	No concerns	No concerns	High
1 vs 7	0	No concerns	Low risk	No concerns	Some concerns	No concerns	No concerns	Moderate
1 vs 9	0	No concerns	Low risk	No concerns	No concerns	No concerns	No concerns	High
2 vs 5	0	No concerns	Low risk	No concerns	Major concerns	No concerns	No concerns	Low
2 vs 6	0	No concerns	Low risk	No concerns	No concerns	No concerns	No concerns	High
2 vs 7	0	No concerns	Low risk	No concerns	Some concerns	Some concerns	No concerns	Low
2 vs 8	0	No concerns	Low risk	No concerns	No concerns	No concerns	No concerns	High
2 vs 9	0	No concerns	Low risk	No concerns	No concerns	No concerns	No concerns	High
3 vs 4	0	No concerns	Low risk	No concerns	Major concerns	No concerns	No concerns	Low
3 vs 5	0	No concerns	Low risk	No concerns	Some concerns	No concerns	No concerns	Moderate
4 vs 5	0	No concerns	Low risk	No concerns	Major concerns	No concerns	No concerns	Low
4 vs 6	0	No concerns	Low risk	No concerns	Some concerns	Some concerns	No concerns	Low
4 vs 7	0	No concerns	Low risk	No concerns	Major concerns	No concerns	No concerns	Low
4 vs 8	0	No concerns	Low risk	No concerns	No concerns	No concerns	No concerns	High

4 vs 9	0	No concerns	Low risk	No concerns	No concerns	No concerns	No concerns	High
5 vs 7	0	No concerns	Low risk	No concerns	Some concerns	Some concerns	No concerns	Low
5 vs 8	0	No concerns	Low risk	No concerns	No concerns	No concerns	No concerns	High
5 vs 9	0	No concerns	Low risk	No concerns	No concerns	No concerns	No concerns	High
6 vs 7	0	No concerns	Low risk	No concerns	Some concerns	No concerns	No concerns	Moderate
6 vs 8	0	No concerns	Low risk	No concerns	Some concerns	No concerns	No concerns	Moderate
6 vs 9	0	No concerns	Low risk	No concerns	No concerns	No concerns	No concerns	High
7 vs 8	0	No concerns	Low risk	No concerns	No concerns	No concerns	No concerns	High
8 vs 9	0	No concerns	Low risk	No concerns	Major concerns	No concerns	No concerns	Low
NMA for ORR in first-line setting (1=DCT, 2=DCT-anti-EGFR, 3=DCT-anti-VEGF, 4=DCT-anti-EGFR/VEGF, 5=TCT-anti-EGFR, 6=TCT-anti-VEGF, 7=Anti-EGFR/BRAF, 8=SCT-anti-EGFR/BRAF, 9=DCT-anti-EGFR/BRAF)								
Mixed evidence								
1 vs 2	2	No concerns	Low risk	No concerns	Some concerns	No concerns	No concerns	Moderate
1 vs 3	1	No concerns	Low risk	No concerns	Some concerns	No concerns	No concerns	Moderate
1 vs 8	1	No concerns	Low risk	No concerns	No concerns	No concerns	No concerns	High
2 vs 3	3	No concerns	Low risk	No concerns	Major concerns	No concerns	Major concerns	Very low
2 vs 5	1	No concerns	Low risk	No concerns	Major concerns	No concerns	Some concerns	Very low
3 vs 4	1	Some concerns	Low risk	No concerns	Major concerns	No concerns	No concerns	Very low
3 vs 5	1	No concerns	Low risk	No concerns	Major concerns	No concerns	No concerns	Low
3 vs 6	3	No concerns	Low risk	No concerns	No concerns	No concerns	No concerns	High
3 vs 7	1	No concerns	Low risk	No concerns	Some concerns	No concerns	No concerns	Moderate
3 vs 8	2	No concerns	Low risk	No concerns	No concerns	No concerns	No concerns	High
3 vs 9	1	No concerns	Low risk	No concerns	No concerns	No concerns	No concerns	High
5 vs 6	1	No concerns	Low risk	No concerns	Some concerns	No concerns	Some concerns	Low
6 vs 8	1	No concerns	Low risk	No concerns	No concerns	No concerns	No concerns	High

7 vs 9	1	No concerns	Low risk	No concerns	No concerns	No concerns	No concerns	High
Indirect evidence								
1 vs 4	0	No concerns	Low risk	No concerns	Some concerns	Some concerns	No concerns	Low
1 vs 5	0	No concerns	Low risk	No concerns	Some concerns	No concerns	No concerns	Moderate
1 vs 6	0	No concerns	Low risk	No concerns	No concerns	No concerns	No concerns	High
1 vs 7	0	No concerns	Low risk	No concerns	No concerns	Some concerns	No concerns	Moderate
1 vs 9	0	No concerns	Low risk	No concerns	No concerns	No concerns	No concerns	High
2 vs 4	0	No concerns	Low risk	No concerns	Major concerns	No concerns	No concerns	Low
2 vs 6	0	No concerns	Low risk	No concerns	Some concerns	No concerns	No concerns	Moderate
2 vs 7	0	No concerns	Low risk	No concerns	Some concerns	Some concerns	No concerns	Low
2 vs 8	0	No concerns	Low risk	No concerns	No concerns	No concerns	No concerns	High
2 vs 9	0	No concerns	Low risk	No concerns	No concerns	No concerns	No concerns	High
4 vs 5	0	No concerns	Low risk	No concerns	Major concerns	No concerns	No concerns	Low
4 vs 6	0	Some concerns	Low risk	No concerns	Major concerns	No concerns	No concerns	Very low
4 vs 7	0	No concerns	Low risk	No concerns	Major concerns	No concerns	No concerns	High
4 vs 8	0	No concerns	Low risk	No concerns	No concerns	Some concerns	No concerns	Moderate
4 vs 9	0	No concerns	Low risk	No concerns	Some concerns	No concerns	No concerns	Moderate
5 vs 7	0	No concerns	Low risk	No concerns	Major concerns	No concerns	No concerns	Low
5 vs 8	0	No concerns	Low risk	No concerns	No concerns	No concerns	No concerns	High
5 vs 9	0	No concerns	Low risk	No concerns	Some concerns	No concerns	No concerns	Moderate
6 vs 7	0	No concerns	Low risk	No concerns	Some concerns	No concerns	No concerns	Moderate
6 vs 9	0	No concerns	Low risk	No concerns	Some concerns	No concerns	No concerns	Moderate
7 vs 8	0	No concerns	Low risk	No concerns	No concerns	No concerns	No concerns	High
8 vs 9	0	No concerns	Low risk	No concerns	Some concerns	No concerns	No concerns	Moderate

NMA for OS in second- or later-line setting (1=Chemo, 2=Chemo-anti-EGFR, 3=Chemo-anti-EGFR/BRAF, 4=Anti-EGFR/BRAF, 5=Anti-EGFR/MEK, 6=Anti-EGFR/BRAF/PI3K, 7=Anti-EGFR/BRAF/MEK)								
Mixed evidence								
1 vs 2	2	No concerns	Low risk	No concerns	Some concerns	Some concerns	No concerns	Low
2 vs 3	1	No concerns	Low risk	No concerns	Some concerns	Some concerns	No concerns	Low
2 vs 4	2	No concerns	Low risk	No concerns	No concerns	Some concerns	No concerns	Moderate
2 vs 7	1	No concerns	Low risk	No concerns	No concerns	Some concerns	No concerns	Moderate
4 vs 5	1	No concerns	Low risk	No concerns	Major concerns	No concerns	No concerns	Low
4 vs 6	1	No concerns	Low risk	No concerns	Major concerns	No concerns	No concerns	Low
4 vs 7	3	No concerns	Low risk	No concerns	No concerns	Major concerns	No concerns	Low
1 vs 3	0	No concerns	Low risk	No concerns	Major concerns	No concerns	No concerns	Low
1 vs 4	0	No concerns	Low risk	No concerns	Some concerns	Some concerns	No concerns	Low
1 vs 5	0	No concerns	Low risk	No concerns	Major concerns	No concerns	No concerns	Low
1 vs 6	0	No concerns	Low risk	No concerns	Major concerns	No concerns	No concerns	Low
1 vs 7	0	No concerns	Low risk	No concerns	Some concerns	Some concerns	No concerns	Low
2 vs 5	0	No concerns	Low risk	No concerns	Major concerns	No concerns	No concerns	Low
2 vs 6	0	No concerns	Low risk	No concerns	Major concerns	No concerns	No concerns	Low
3 vs 4	0	No concerns	Low risk	No concerns	Major concerns	No concerns	No concerns	Low
3 vs 5	0	No concerns	Low risk	No concerns	Major concerns	No concerns	No concerns	Low
3 vs 6	0	No concerns	Low risk	No concerns	Major concerns	No concerns	No concerns	Low
3 vs 7	0	No concerns	Low risk	No concerns	Some concerns	Some concerns	No concerns	Low
5 vs 6	0	No concerns	Low risk	No concerns	Major concerns	No concerns	No concerns	Low
5 vs 7	0	No concerns	Low risk	No concerns	Some concerns	Some concerns	No concerns	Low
6 vs 7	0	No concerns	Low risk	No concerns	Major concerns	No concerns	No concerns	Low

NMA for PFS in second- or later-line setting (1=Chemo, 2=Chemo-anti-EGFR, 3=Chemo-anti-EGFR/BRAF, 4=Anti-EGFR/BRAF, 5=Anti- EGFR/MEK, 6=Anti-EGFR/BRAF/PI3K, 7=Anti-EGFR/BRAF/MEK)								
Mixed evidence								
1 vs 2	2	No concerns	Low risk	No concerns	Major concerns	No concerns	No concerns	Low
2 vs 3	1	No concerns	Low risk	No concerns	No concerns	Some concerns	No concerns	Moderate
2 vs 4	2	No concerns	Low risk	No concerns	No concerns	No concerns	No concerns	High
2 vs 7	1	No concerns	Low risk	No concerns	No concerns	No concerns	No concerns	High
4 vs 5	1	No concerns	Low risk	No concerns	Some concerns	Some concerns	No concerns	Low
4 vs 6	1	No concerns	Low risk	No concerns	Some concerns	Some concerns	No concerns	Low
4 vs 7	3	No concerns	Low risk	No concerns	Some concerns	Some concerns	No concerns	Low
Indirect evidence								
1 vs 3	0	No concerns	Low risk	No concerns	Some concerns	Some concerns	No concerns	Low
1 vs 4	0	No concerns	Low risk	No concerns	No concerns	Some concerns	No concerns	Moderate
1 vs 5	0	No concerns	Low risk	No concerns	Major concerns	No concerns	No concerns	Low
1 vs 6	0	No concerns	Low risk	No concerns	No concerns	Some concerns	No concerns	Moderate
1 vs 7	0	No concerns	Low risk	No concerns	No concerns	Some concerns	No concerns	Moderate
2 vs 5	0	No concerns	Low risk	No concerns	Major concerns	No concerns	No concerns	Low
2 vs 6	0	No concerns	Low risk	No concerns	No concerns	No concerns	No concerns	High
3 vs 4	0	No concerns	Low risk	No concerns	Major concerns	No concerns	No concerns	Low
3 vs 5	0	No concerns	Low risk	No concerns	Major concerns	No concerns	No concerns	Low
3 vs 6	0	No concerns	Low risk	No concerns	Major concerns	No concerns	No concerns	Low
3 vs 7	0	No concerns	Low risk	No concerns	Some concerns	Some concerns	No concerns	Low
5 vs 6	0	No concerns	Low risk	No concerns	No concerns	Major concerns	No concerns	Low
5 vs 7	0	No concerns	Low risk	No concerns	No concerns	Major concerns	No concerns	Low
6 vs 7	0	No concerns	Low risk	No concerns	Major concerns	No concerns	No concerns	Low

NMA for ORR in second- or later-line setting (1=Chemo, 2=Chemo-anti-EGFR, 3=Chemo-anti-EGFR/BRAF, 4=Anti-EGFR/BRAF, 5=Anti-EGFR/BRAF/PI3K, 6=Anti-EGFR/BRAF/MEK)								
Mixed evidence								
1 vs 2	1	No concerns	Low risk	No concerns	Major concerns	No concerns	No concerns	Low
2 vs 3	1	No concerns	Low risk	No concerns	Some concerns	Some concerns	No concerns	Low
2 vs 4	2	No concerns	Low risk	No concerns	No concerns	No concerns	No concerns	High
2 vs 6	1	No concerns	Low risk	No concerns	No concerns	No concerns	No concerns	High
4 vs 5	1	No concerns	Low risk	No concerns	Some concerns	Some concerns	No concerns	Low
4 vs 6	4	No concerns	Low risk	No concerns	No concerns	Some concerns	No concerns	Moderate
Indirect evidence								
1 vs 3	0	No concerns	Low risk	No concerns	Some concerns	Some concerns	No concerns	Low
1 vs 4	0	No concerns	Low risk	No concerns	No concerns	Some concerns	No concerns	Moderate
1 vs 5	0	No concerns	Low risk	No concerns	No concerns	Some concerns	No concerns	Moderate
1 vs 6	0	No concerns	Low risk	No concerns	No concerns	No concerns	No concerns	High
2 vs 5	0	No concerns	Low risk	No concerns	No concerns	No concerns	No concerns	High
3 vs 4	0	No concerns	Low risk	No concerns	Major concerns	No concerns	No concerns	Low
3 vs 5	0	No concerns	Low risk	No concerns	Major concerns	No concerns	No concerns	Low
3 vs 6	0	No concerns	Low risk	No concerns	Some concerns	Some concerns	No concerns	Low
5 vs 6	0	No concerns	Low risk	No concerns	Major concerns	No concerns	No concerns	Low
NMA for DCR in second- or later-line setting (1=Chemo-anti-EGFR, 2=Chemo-anti-EGFR/BRAF, 3=Anti-EGFR/BRAF, 4=Anti-EGFR/BRAF/MEK)								
Mixed evidence								
1 vs 2	1	No concerns	Low risk	No concerns	No concerns	Major concerns	Some concerns	Very low
1 vs 3	1	No concerns	Low risk	No concerns	No concerns	Major concerns	No concerns	Low
1 vs 4	1	No concerns	Low risk	No concerns	No concerns	Major concerns	No concerns	Low
3 vs 4	3	No concerns	Low risk	No concerns	Some concerns	Some concerns	Some concerns	Very low

Indirect evidence								
2 vs 3	0	No concerns	Low risk	No concerns	Major concerns	No concerns	Some concerns	Very low
2 vs 4	0	No concerns	Low risk	No concerns	Major concerns	No concerns	Some concerns	Very low
NMA for (Grade 3 or higher AEs) in second- or later-line setting (1=Chemo-anti-EGFR, 2=Chemo-anti-EGFR/BRAF, 3=Anti- EGFR/MEK, 4=Anti-EGFR/BRAF, 5=Anti-EGFR/BRAF/PI3K, 6=Anti-EGFR/BRAF/MEK								
Mixed evidence								
1 vs 2	1	No concerns	Low risk	No concerns	No concerns	Major concerns	No concerns	Low
1 vs 4	2	No concerns	Low risk	No concerns	Some concerns	Some concerns	No concerns	Low
1 vs 6	1	No concerns	Low risk	No concerns	Some concerns	Some concerns	No concerns	Low
3 vs 4	1	No concerns	Low risk	No concerns	Some concerns	Some concerns	No concerns	Low
3 vs 6	1	No concerns	Low risk	No concerns	Major concerns	No concerns	No concerns	Low
4 vs 5	1	No concerns	Low risk	No concerns	Some concerns	Some concerns	No concerns	Low
4 vs 6	4	No concerns	Low risk	No concerns	No concerns	Major concerns	No concerns	Low
Indirect evidence								
1 vs 3	0	No concerns	Low risk	No concerns	Major concerns	No concerns	No concerns	Low
1 vs 5	0	No concerns	Low risk	No concerns	Some concerns	Some concerns	No concerns	Low
2 vs 3	0	No concerns	Low risk	No concerns	Some concerns	Some concerns	No concerns	Low
2 vs 4	0	No concerns	Low risk	No concerns	No concerns	Major concerns	No concerns	Low
2 vs 5	0	No concerns	Low risk	No concerns	Major concerns	No concerns	No concerns	Low
2 vs 6	0	No concerns	Low risk	No concerns	Some concerns	Some concerns	No concerns	Low
3 vs 5	0	No concerns	Low risk	No concerns	Major concerns	No concerns	No concerns	Low
5 vs 6	0	No concerns	Low risk	No concerns	Major concerns	No concerns	No concerns	Low

Table 12. Abbreviation list.

95%CI	95% credible intervals
AE	Adverse event
anti-BRAF	BRAF inhibitor
anti-EGFR	EGFR inhibitor
anti-ERK	ERK inhibitor
anti-MEK	MEK inhibitor
anti-PD-1	PD-1 inhibitor
anti-PI3K	PI3K inhibitor
anti-VEGF	VEGF inhibitor
BRAF	B-Raf proto-oncogene, serine/threonine kinase
BRAFmut	BRAF mutation
CRC	Colorectal cancer
DCR	Disease control rate
DCT	Single-agent chemotherapy
EGFR	Epidermal growth factor receptor
HR	Hazard ratios
NMA	Network meta-analysis
OR	Odds ratio
ORR	objective response rate
OS	Overall survival
PD-1	Programmed death receptor 1
PFS	progression-free survival
PRISMA	Preferred Reporting Items for Systematic Reviews and Meta-Analyses
SCT	Single-agent chemotherapy
SUCRA	Surface under the cumulative ranking curve
TCT	Triplet chemotherapy
VEGF	Vascular endothelial growth factor

Figure S1. Assessment of convergence for OS, PFS, ORR, DCR and grade 3 or higher AE in first-line setting using trace plots, density plots, and the Brooks–Gelman–Rubin method[4].

A: Trace plots and density plots for OS (1=DCT, 2=DCT-anti-EGFR, 3=DCT-anti-VEGF, 4=DCT-anti-EGFR/VEGF, 5=TCT-anti-EGFR, 6=TCT-anti-VEGF, 7=Anti-EGFR/BRAF, 8=SCT-anti-EGFR/BRAF, 9=DCT-anti-EGFR/BRAF)

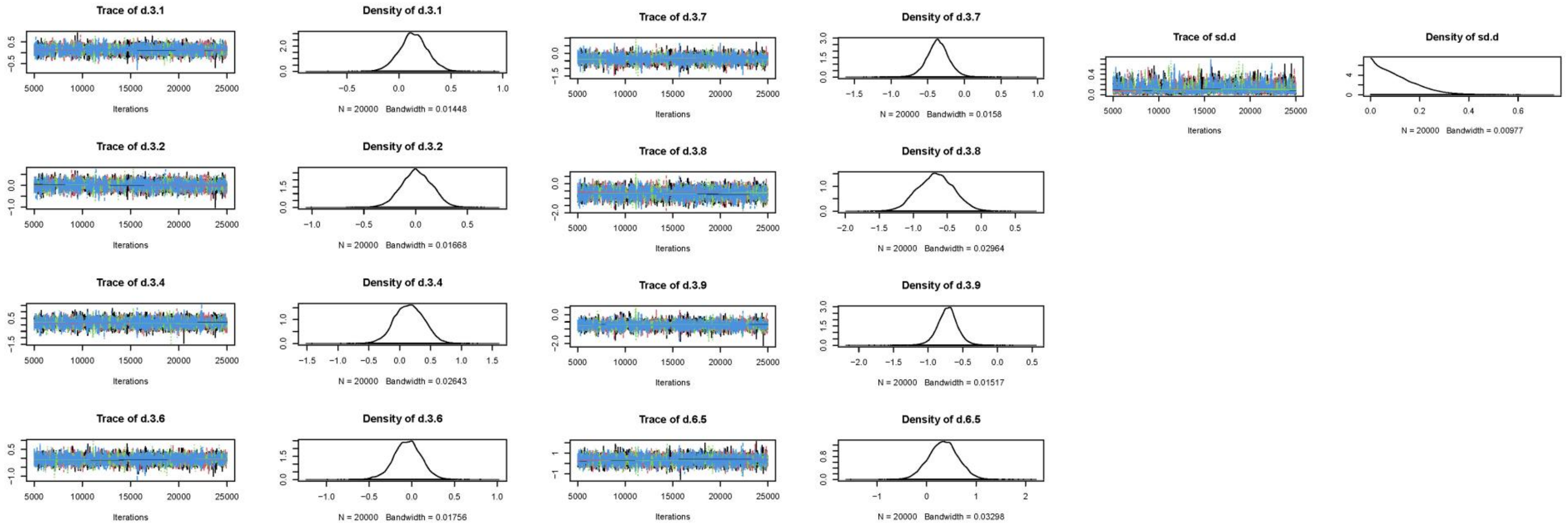

B: Brooks-Gelman-Rubin diagnostic for OS (1=DCT, 2=DCT-anti-EGFR, 3=DCT-anti-VEGF, 4=DCT-anti-EGFR/VEGF, 5=TCT-anti-EGFR, 6=TCT-anti-VEGF, 7=Anti-EGFR/BRAF, 8=SCT-anti-EGFR/BRAF, 9=DCT-anti-EGFR/BRAF)

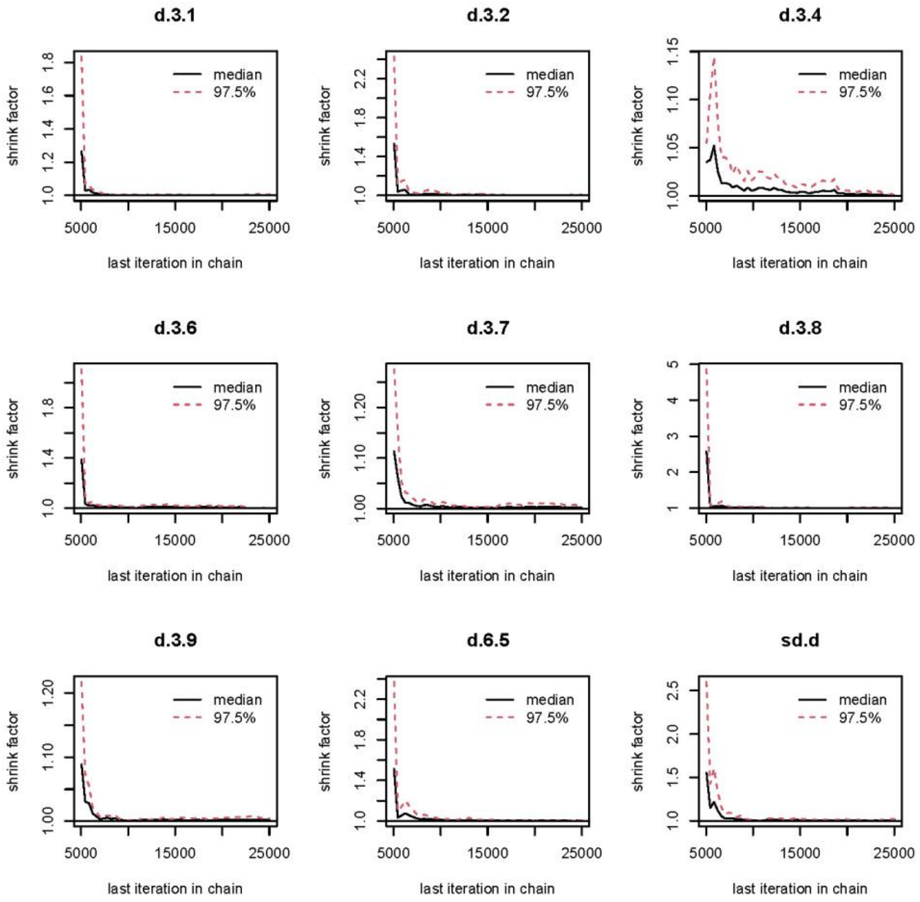

C: Trace plots and density plots for PFS (1=DCT, 2=DCT-anti-EGFR, 3=DCT-anti-VEGF, 4=DCT-anti-EGFR/VEGF, 5=TCT-anti-EGFR, 6=TCT-anti-VEGF, 7=Anti-EGFR/BRAF, 8=SCT-anti-EGFR/BRAF, 9=DCT-anti-EGFR/BRAF)

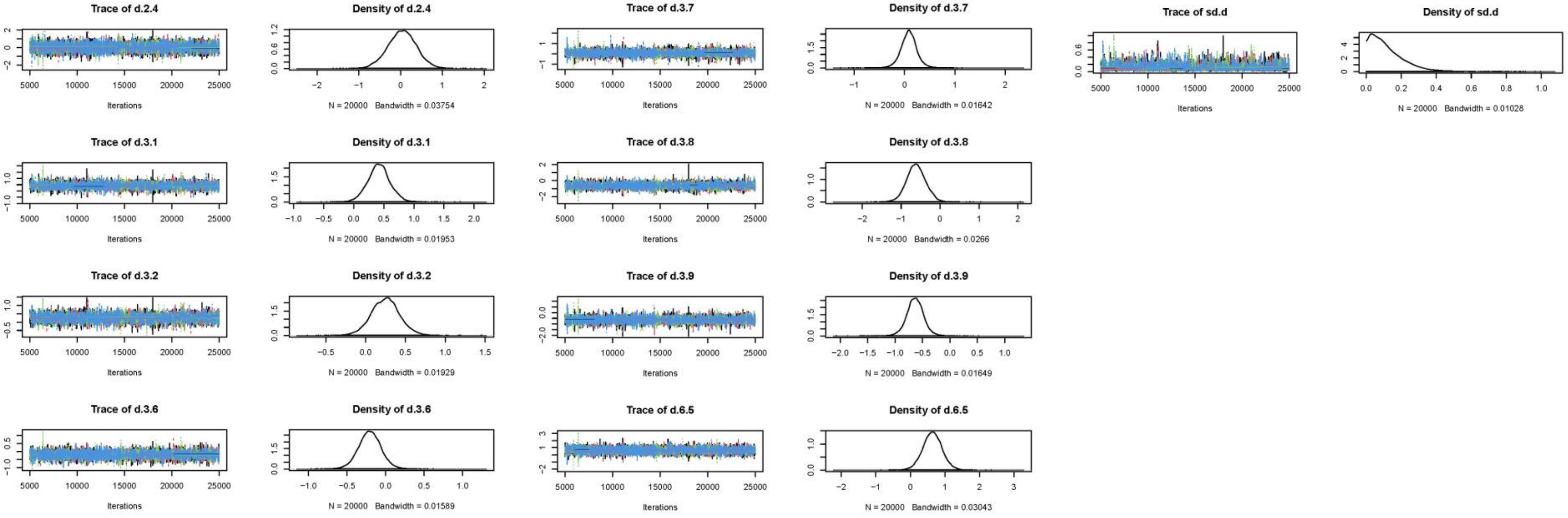

D: Brooks-Gelman-Rubin diagnostic for PFS (1=DCT, 2=DCT-anti-EGFR, 3=DCT-anti-VEGF, 4=DCT-anti-EGFR/VEGF, 5=TCT-anti-EGFR, 6=TCT-anti-VEGF, 7=Anti-EGFR/BRAF, 8=SCT-anti-EGFR/BRAF, 9=DCT-anti-EGFR/BRAF)

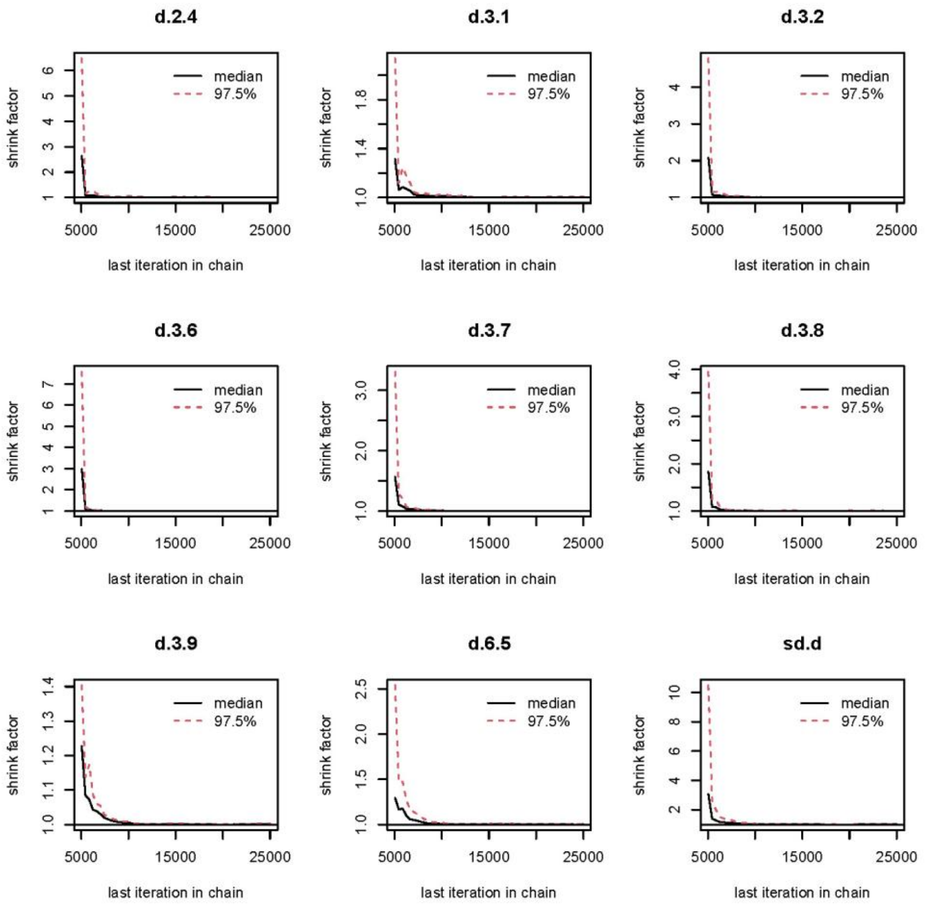

E: Trace plots and density plots for ORR (1=DCT, 2=DCT-anti-EGFR, 3=DCT-anti-VEGF, 4=DCT-anti-EGFR/VEGF, 5=TCT-anti-EGFR, 6=TCT-anti-VEGF, 7=Anti-EGFR/BRAF, 8=SCT-anti-EGFR/BRAF, 9=DCT-anti-EGFR/BRAF)

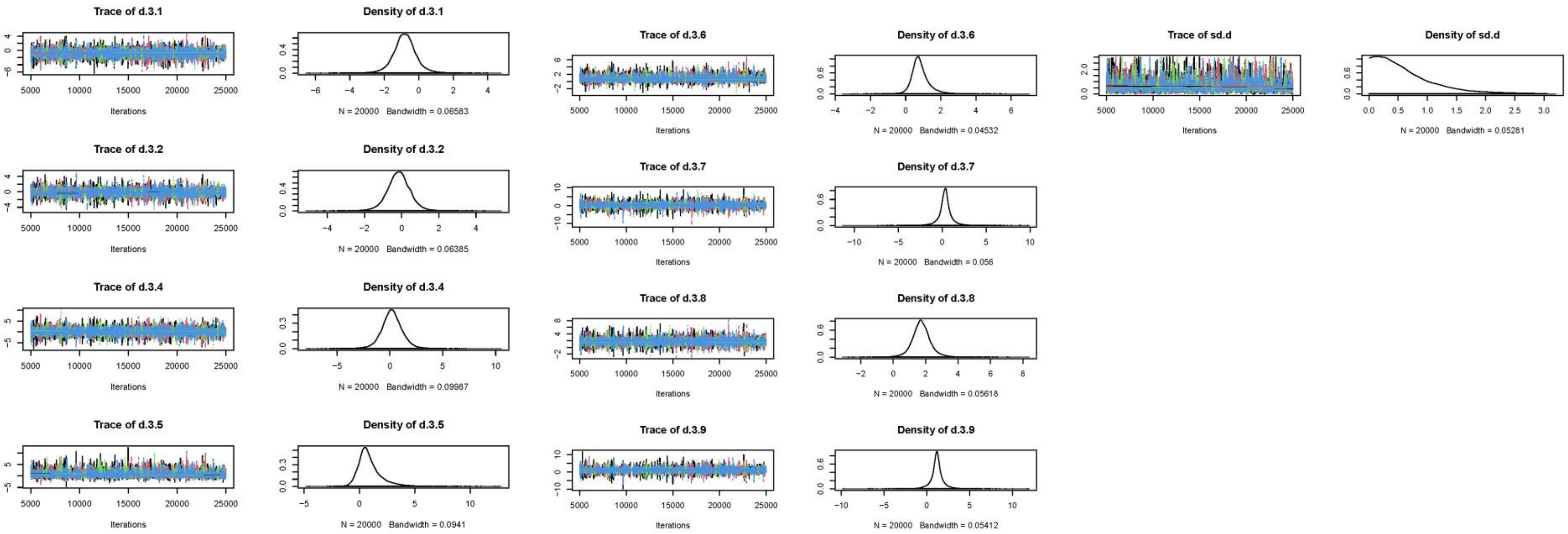

F: Brooks-Gelman-Rubin diagnostic for ORR (1=DCT, 2=DCT-anti-EGFR, 3=DCT-anti-VEGF, 4=DCT-anti-EGFR/VEGF, 5=TCT-anti-EGFR, 6=TCT-anti-VEGF, 7=Anti-EGFR/BRAF, 8=SCT-anti-EGFR/BRAF, 9=DCT-anti-EGFR/BRAF)

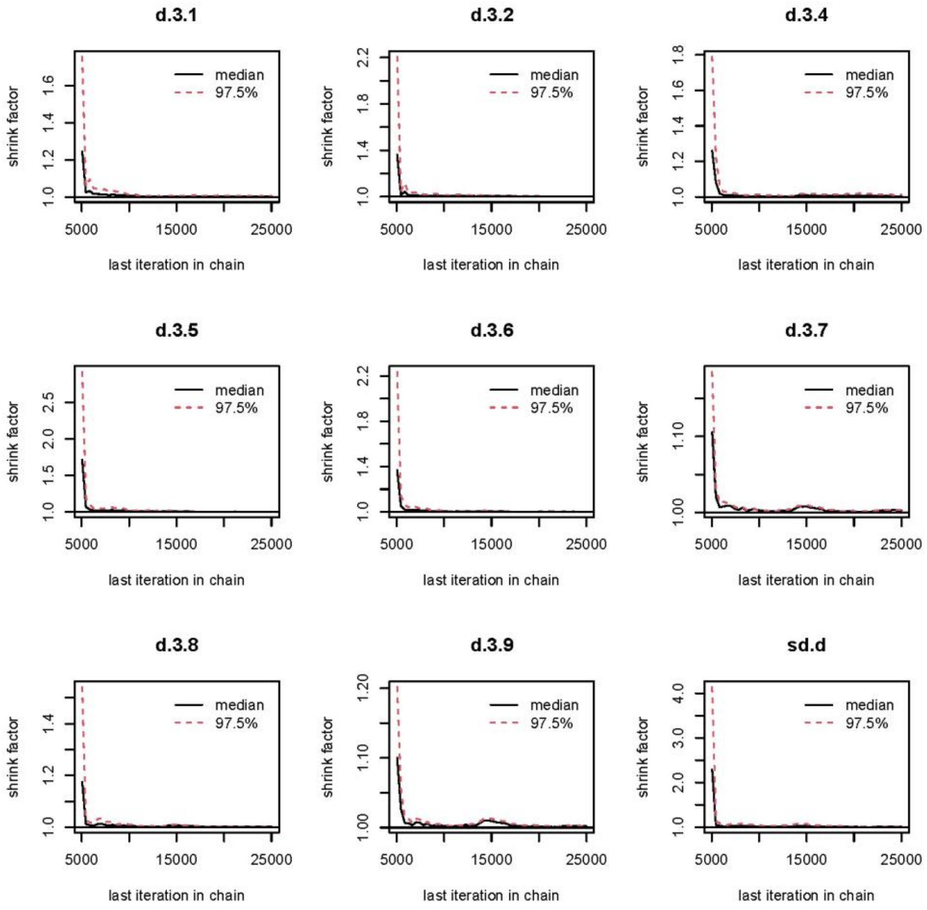

G: Trace plots and density plots for DCR (1=DCT, 2=DCT-anti-EGFR, 3=DCT-anti-VEGF, 4=TCT-anti-EGFR, 5=TCT-anti-VEGF, 6=anti-EGFR/BRAF, 7=SCT-anti-EGFR/BRAF, 8=DCT-anti-EGFR/BRAF)

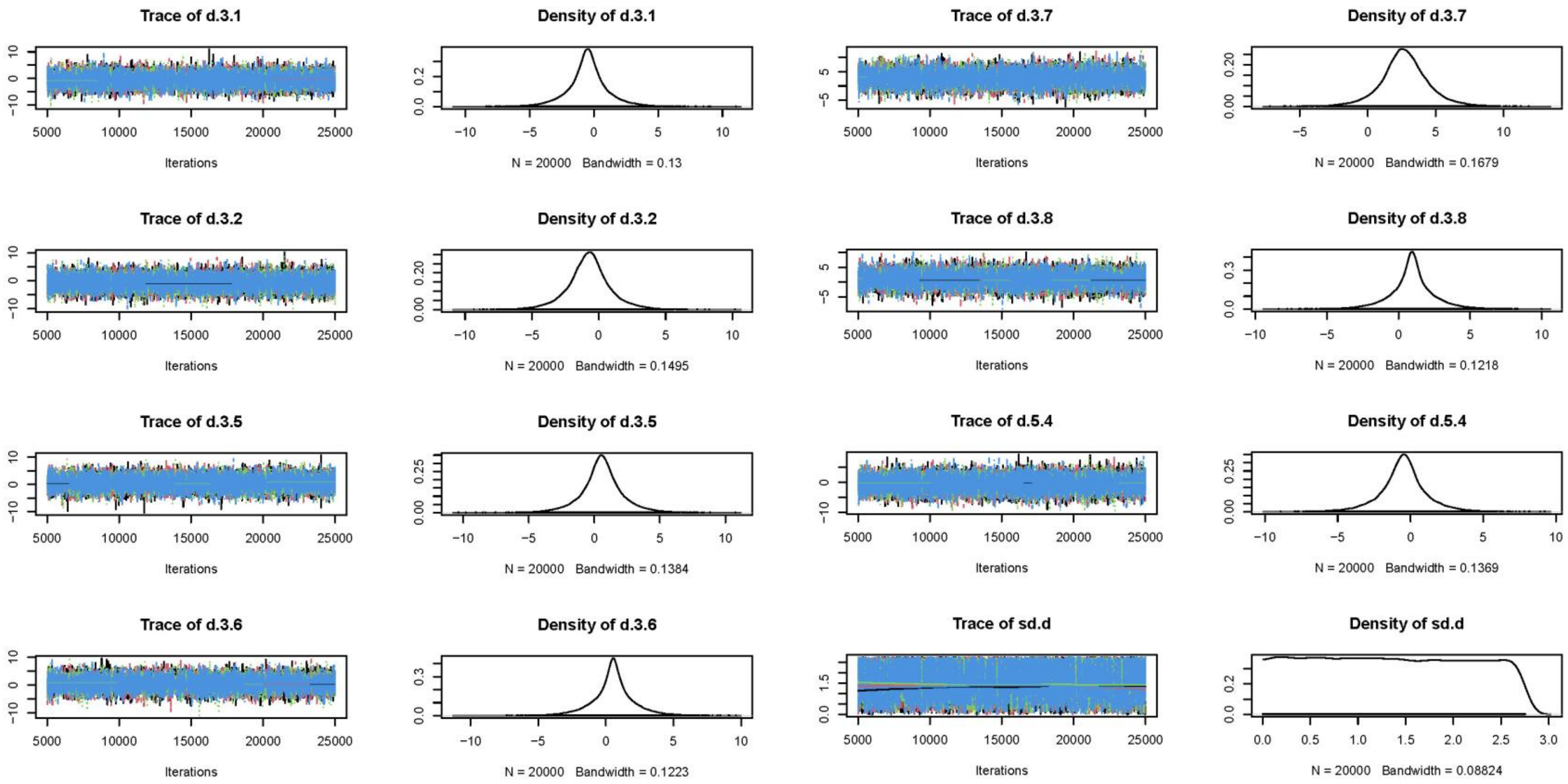

H: Brooks-Gelman-Rubin diagnostic for DCR (1=DCT, 2=DCT-anti-EGFR, 3=DCT-anti-VEGF, 4=TCT-anti-EGFR, 5=TCT-anti-VEGF, 6=anti-EGFR/BRAF, 7=SCT-anti-EGFR/BRAF, 8=DCT-anti-EGFR/BRAF)

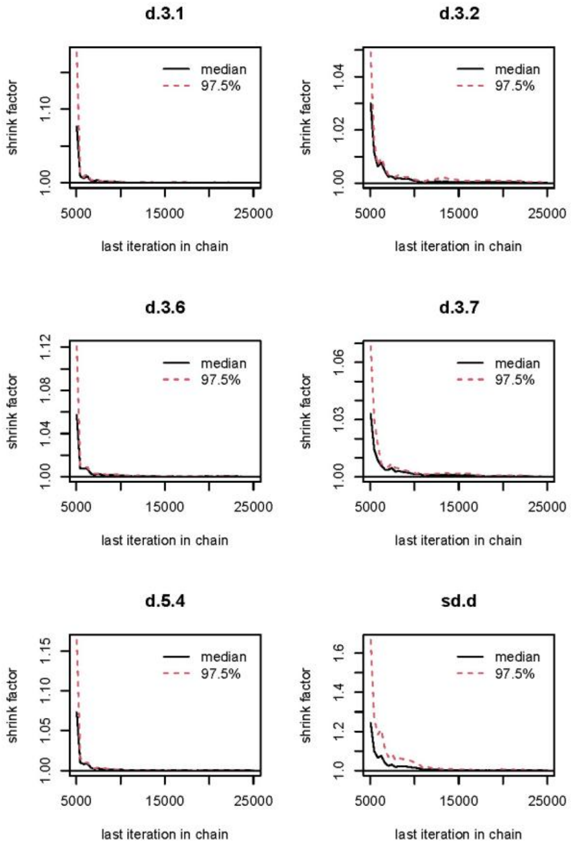

I: Trace plots and density plots for grade 3 or higher AEs (1=DCT-anti-VEGF, 2=TCT-anti-EGFR, 3=anti-EGFR/BRAF, 4=SCT-anti-EGFR/BRAF, 5=DCT-anti-EGFR/BRAF)

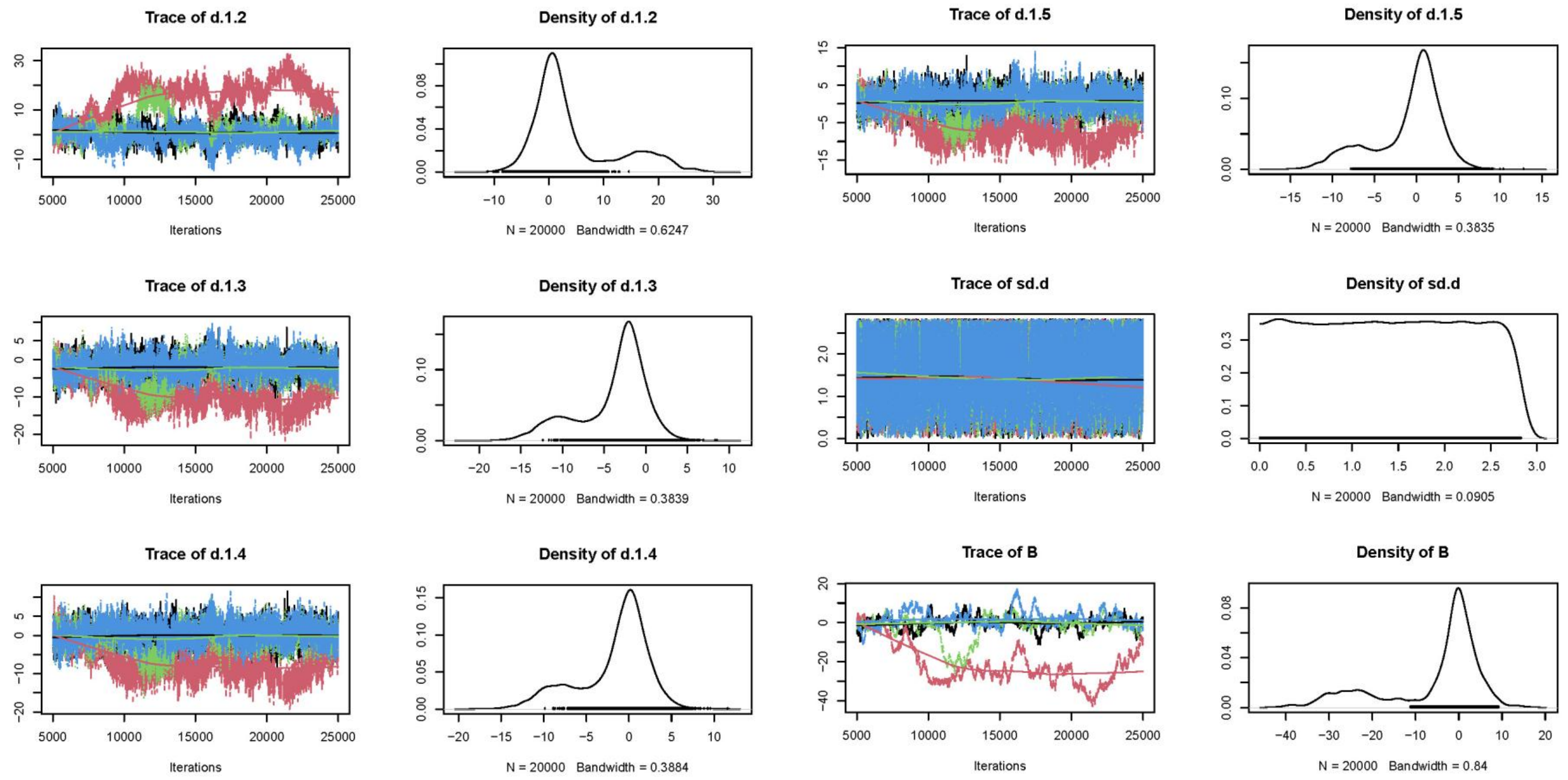

J: Brooks-Gelman-Rubin diagnostic for grade 3 or higher AEs (1=DCT-anti-VEGF, 2=TCT-anti-EGFR, 3=anti-EGFR/BRAF, 4=SCT-anti-EGFR/BRAF, 5=DCT-anti-EGFR/BRAF)

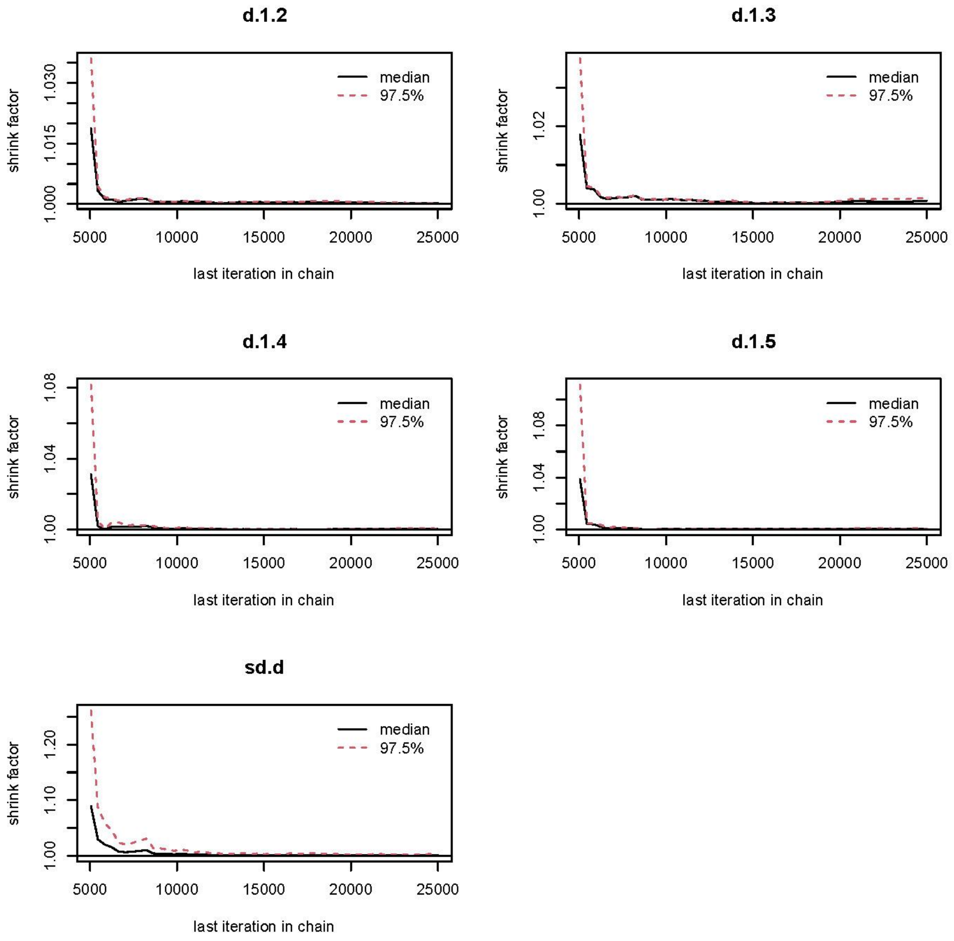

Figure S2. Assessment of convergence for OS, PFS, ORR, DCR and grade 3 or higher AE in second- or later-line setting using trace plots, density plots, and the Brooks–Gelman–Rubin method.

A: Trace plots and density plots for OS (1=Chemo, 2=Chemo-anti-EGFR, 3=Chemo-anti-EGFR/BRAF, 4=Anti-EGFR/BRAF, 5=Anti-EGFR/MEK, 6=Anti-EGFR/BRAF/PI3K, 7=Anti-EGFR/BRAF/MEK)

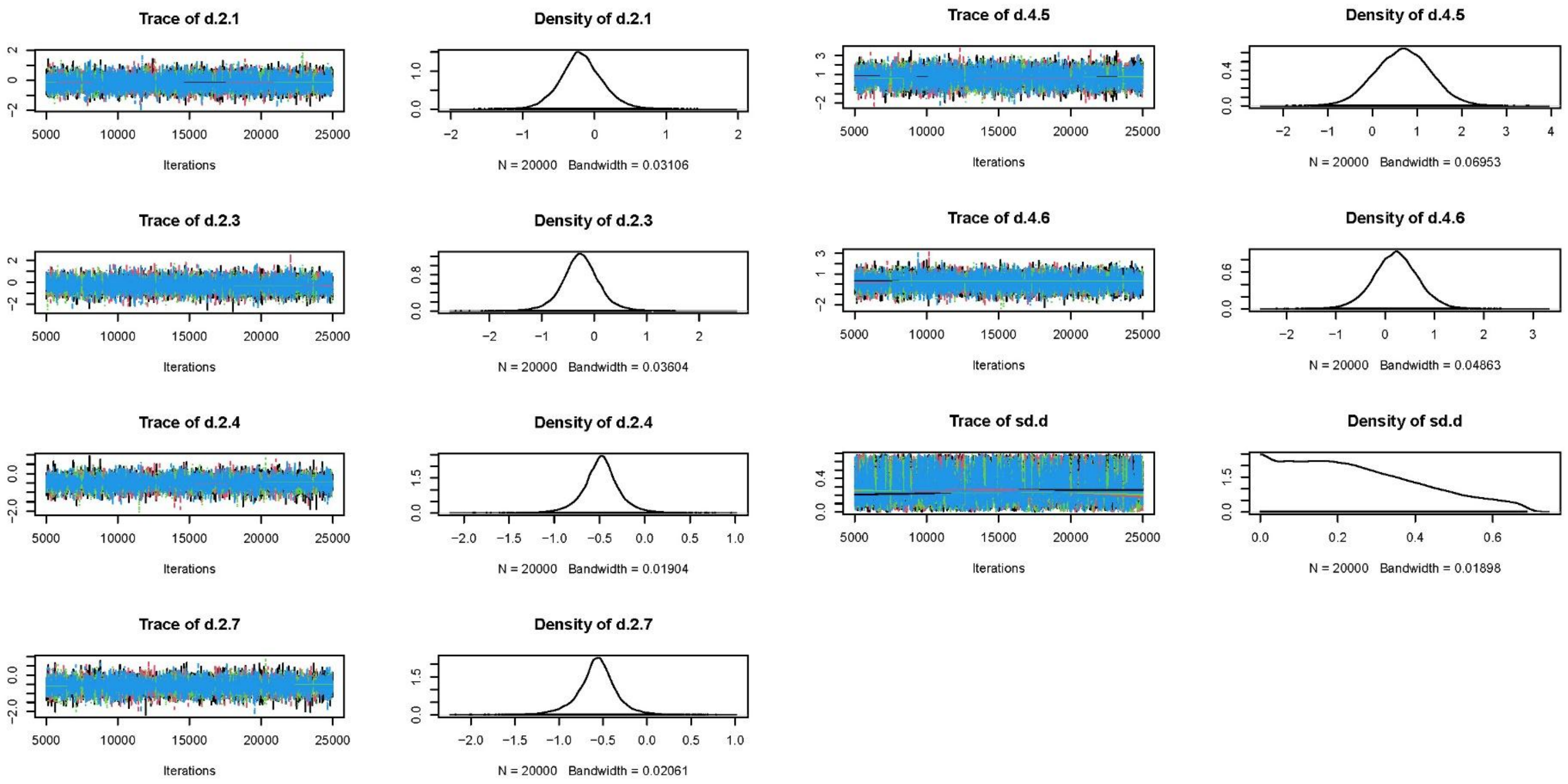

B: Brooks-Gelman-Rubin diagnostic for OS (1=Chemo, 2=Chemo-anti-EGFR, 3=Chemo-anti-EGFR/BRAF, 4=Anti-EGFR/BRAF, 5=Anti- EGFR/MEK, 6=Anti-EGFR/BRAF/PI3K, 7=Anti-EGFR/BRAF/MEK)

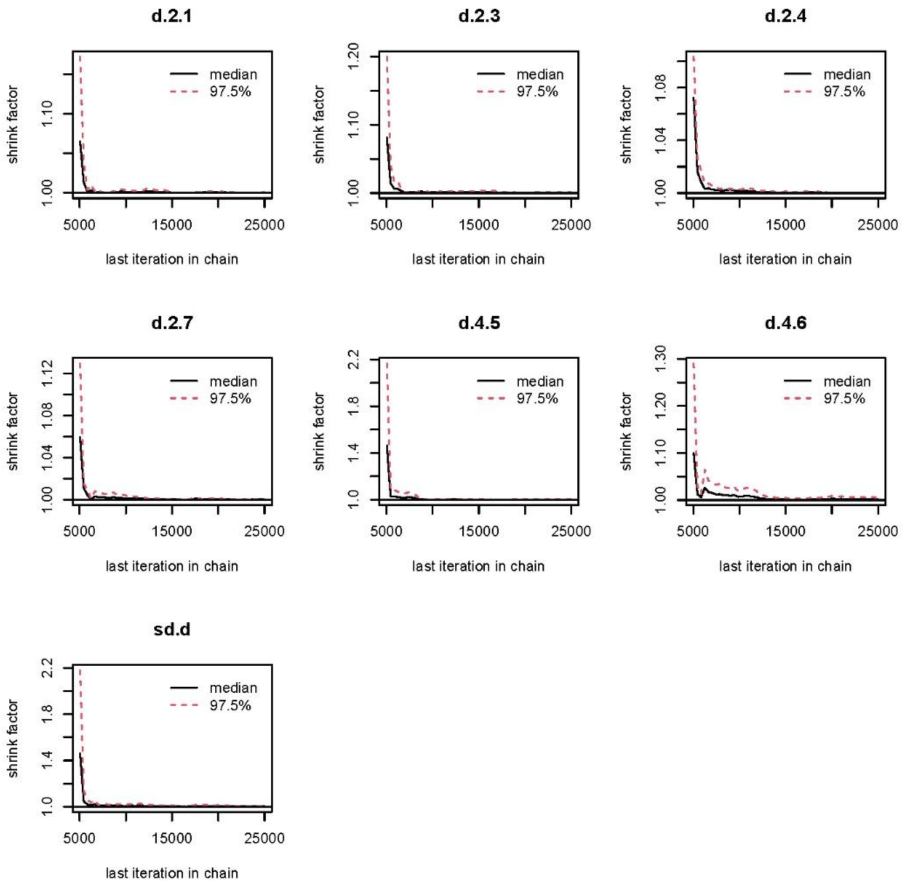

C: Trace plots and density plots for PFS (1=Chemo, 2=Chemo-anti-EGFR, 3=Chemo-anti-EGFR/BRAF, 4=Anti-EGFR/BRAF, 5=Anti- EGFR/MEK, 6=Anti-EGFR/BRAF/PI3K, 7=Anti-EGFR/BRAF/MEK)

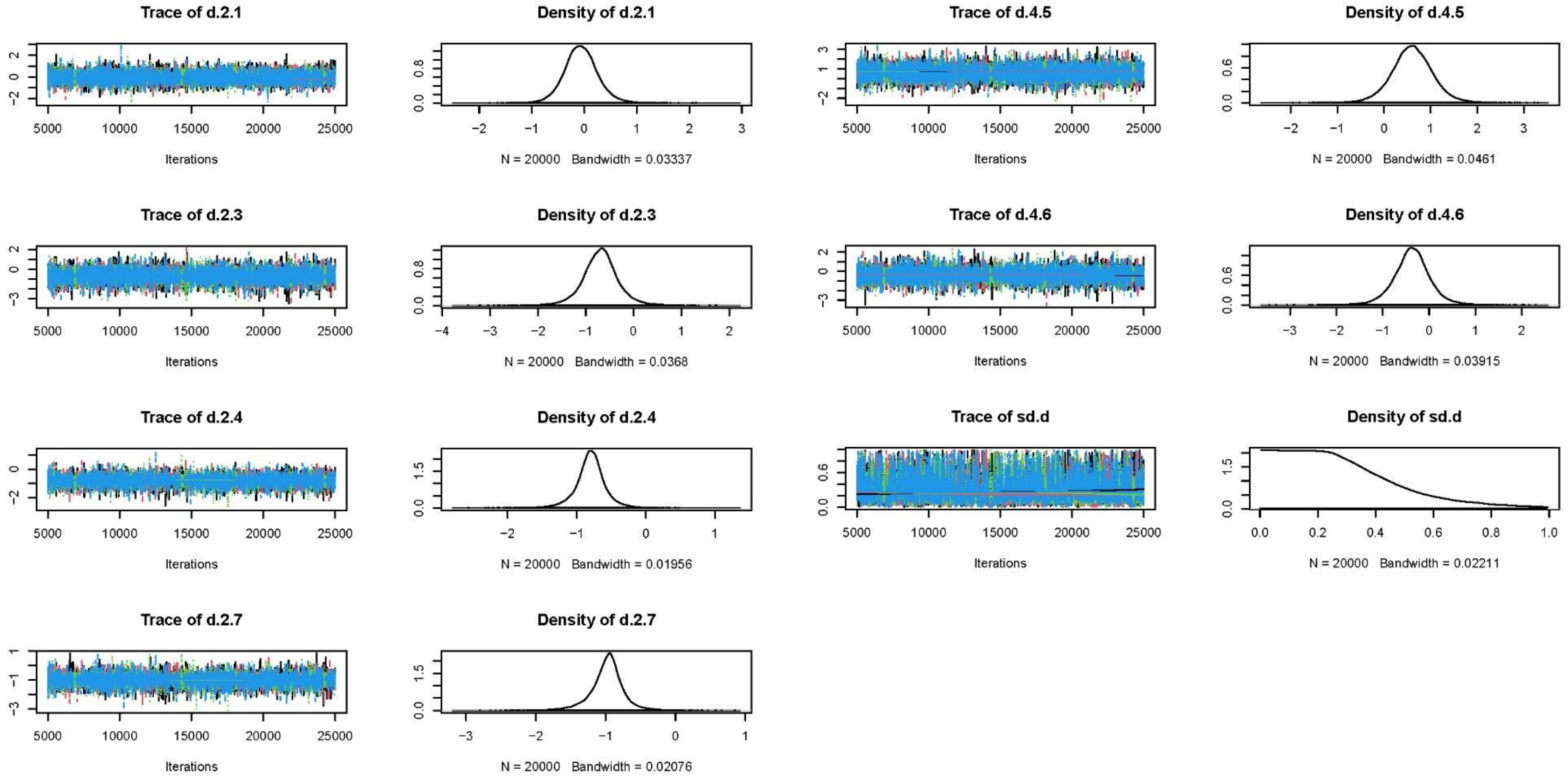

D: Brooks-Gelman-Rubin diagnostic for PFS (1=Chemo, 2=Chemo-anti-EGFR, 3=Chemo-anti-EGFR/BRAF, 4=Anti-EGFR/BRAF, 5=Anti- EGFR/MEK, 6=Anti-EGFR/BRAF/PI3K, 7=Anti-EGFR/BRAF/MEK)

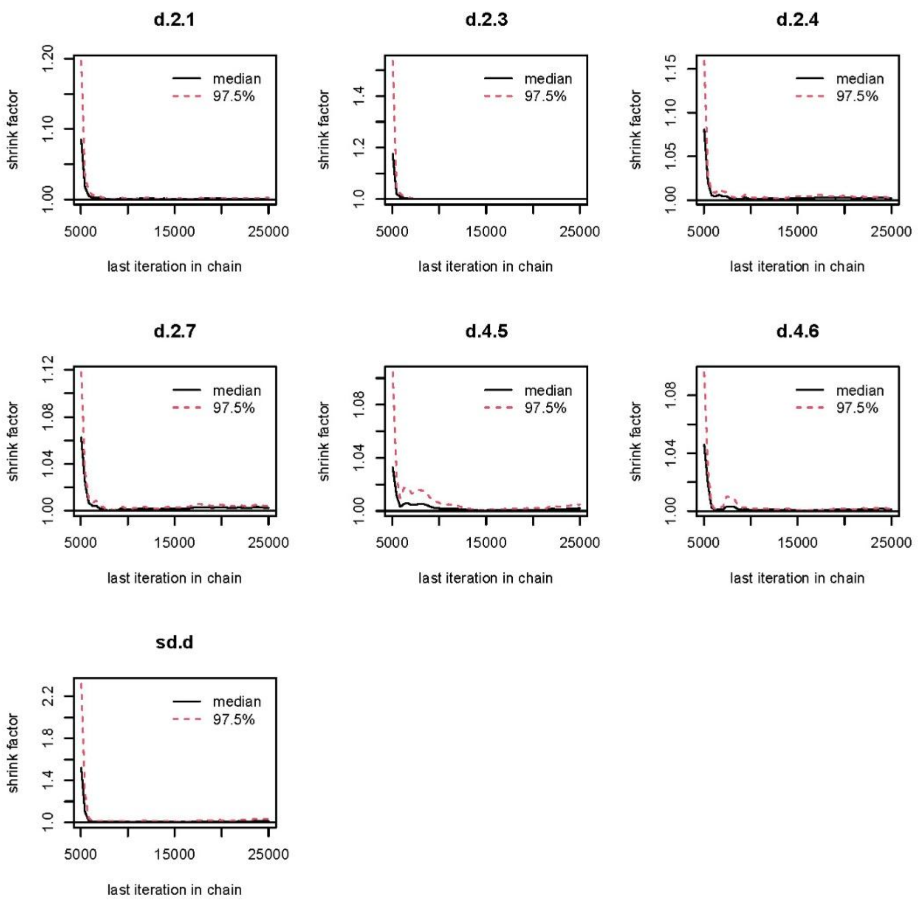

E: Trace plots and density plots for ORR (1=Chemo, 2=Chemo-anti-EGFR, 3=Chemo-anti-EGFR/BRAF, 4=Anti-EGFR/BRAF, 5=Anti-EGFR/BRAF/PI3K, 6=Anti-EGFR/BRAF/MEK)

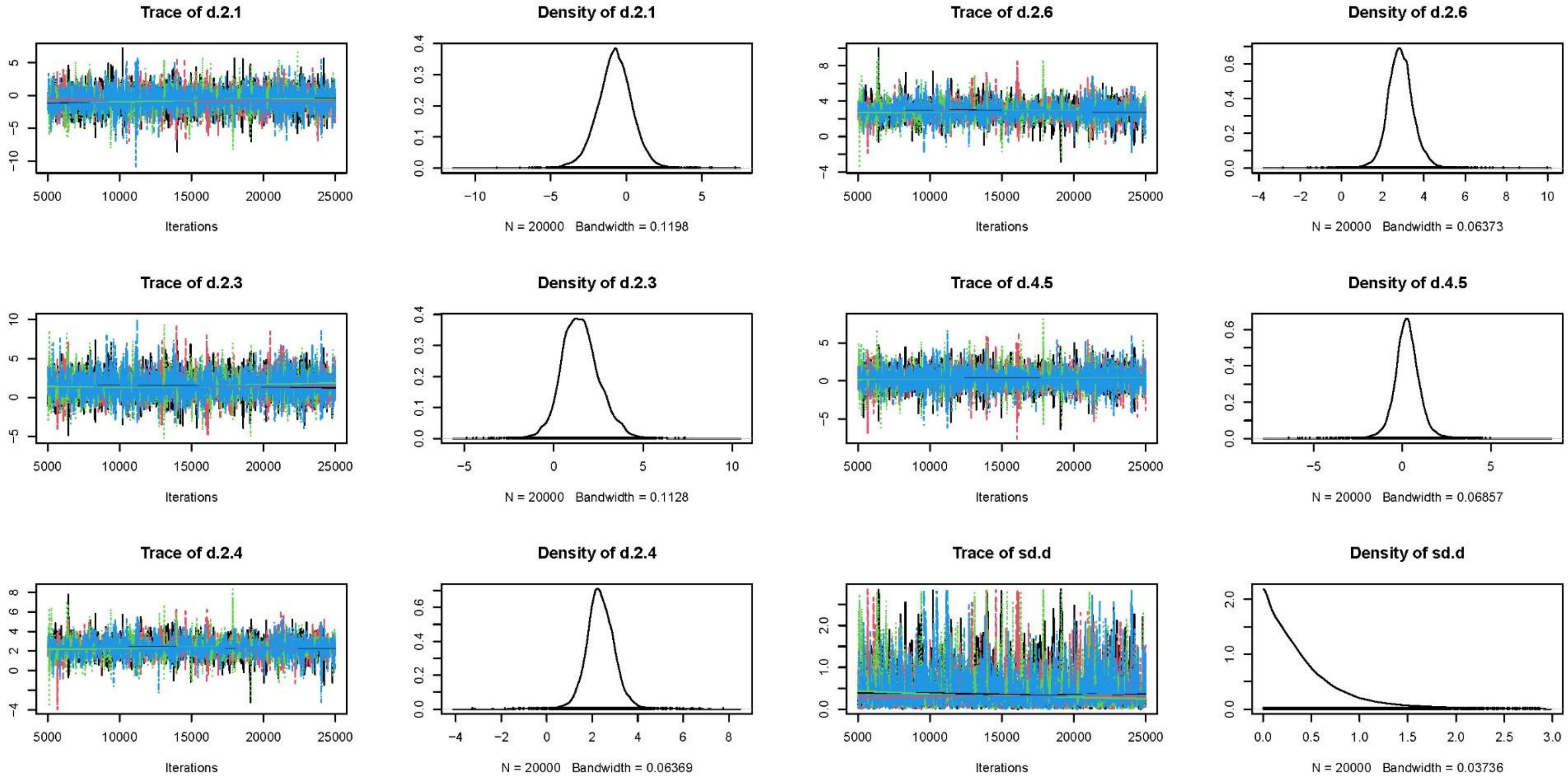

F: Brooks-Gelman-Rubin diagnostic for ORR (1=Chemo, 2=Chemo-anti-EGFR, 3=Chemo-anti-EGFR/BRAF, 4=Anti-EGFR/BRAF, 5=Anti-EGFR/BRAF/PI3K, 6=Anti-EGFR/BRAF/MEK)

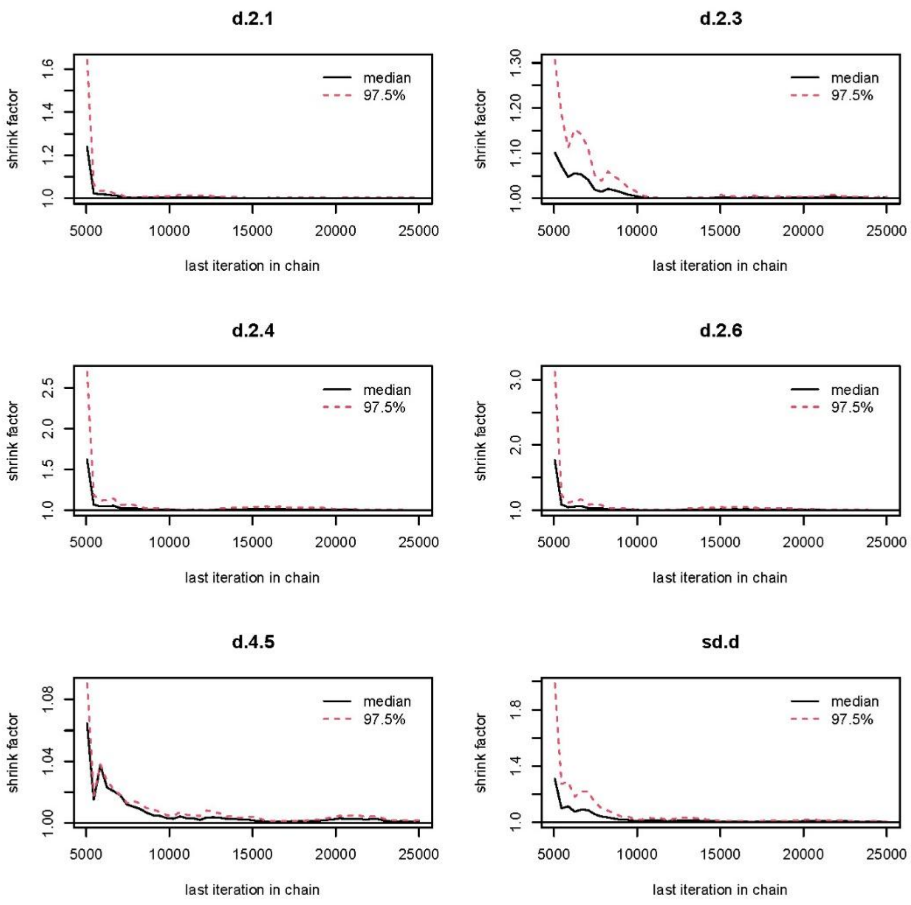

G: Trace plots and density plots for DCR (1=Chemo-anti-EGFR, 2=Chemo-anti-EGFR/BRAF, 3=Anti-EGFR/BRAF, 4=Anti-EGFR/BRAF/MEK)

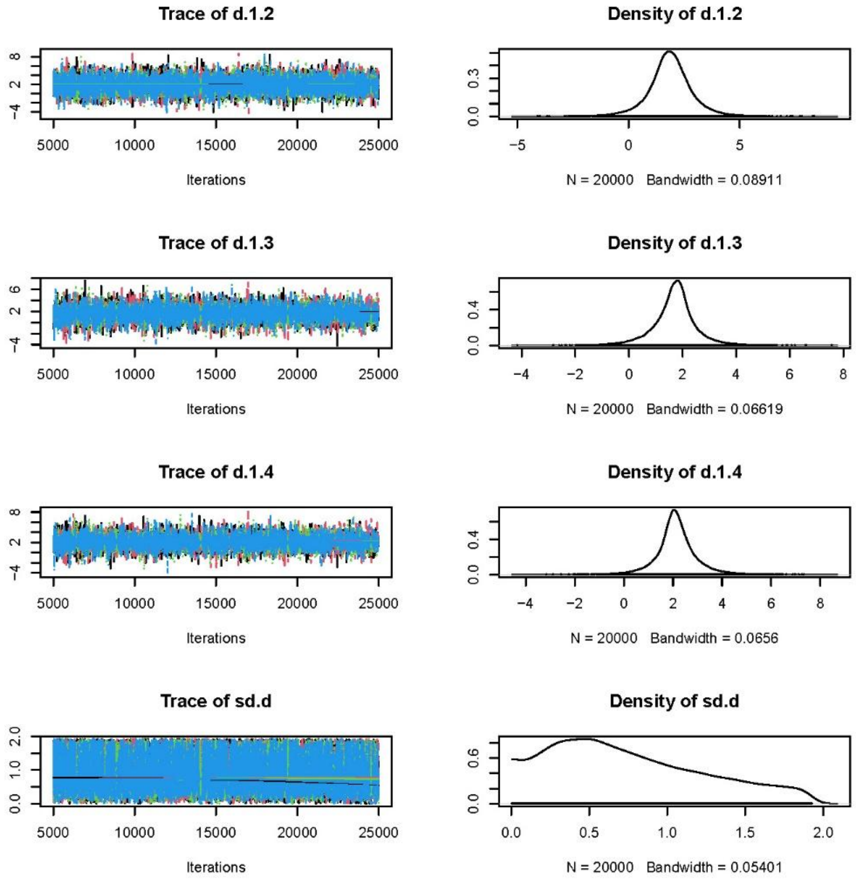

H: Brooks-Gelman-Rubin diagnostic for DCR (1=Chemo-anti-EGFR, 2=Chemo-anti-EGFR/BRAF, 3=Anti-EGFR/BRAF, 4=Anti-EGFR/BRAF/MEK)

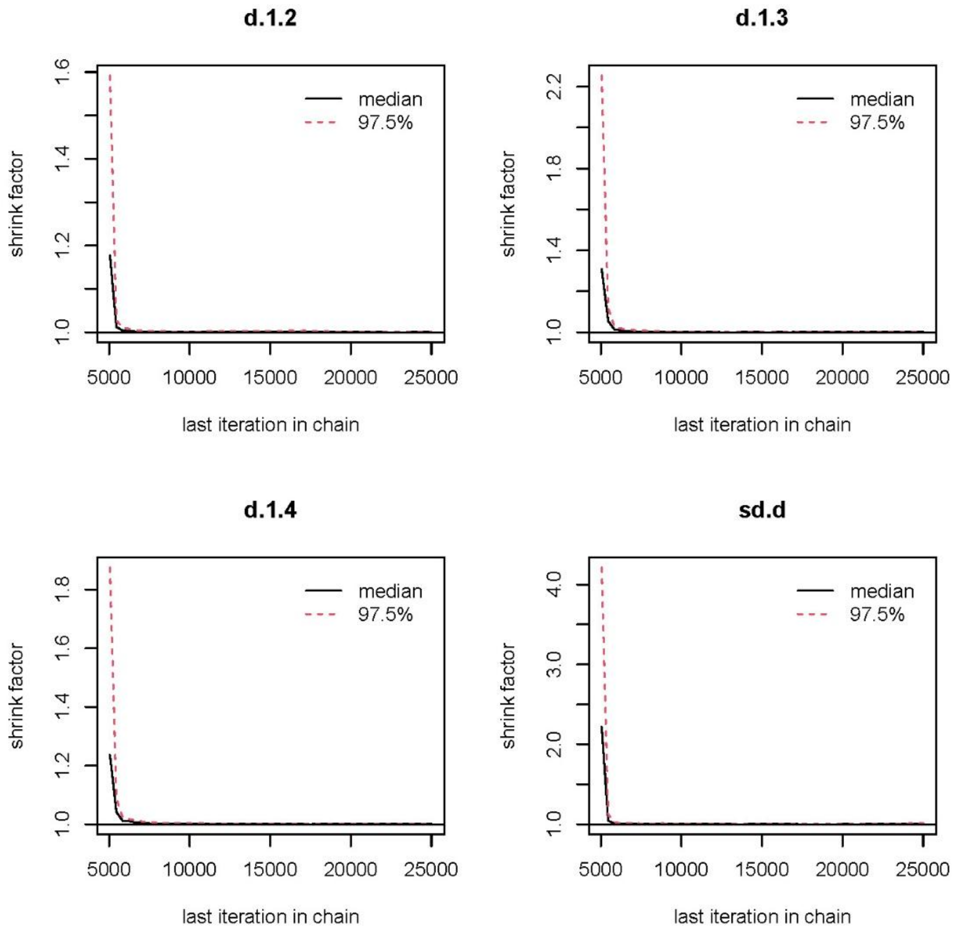

I: Trace plots and density plots for Grade 3 or higher AEs (1=Chemo-anti-EGFR, 2=Chemo-anti-EGFR/BRAF, 3=Anti- EGFR/MEK, 4=Anti-EGFR/BRAF, 5=Anti-EGFR/BRAF/PI3K, 6=Anti-EGFR/BRAF/MEK)

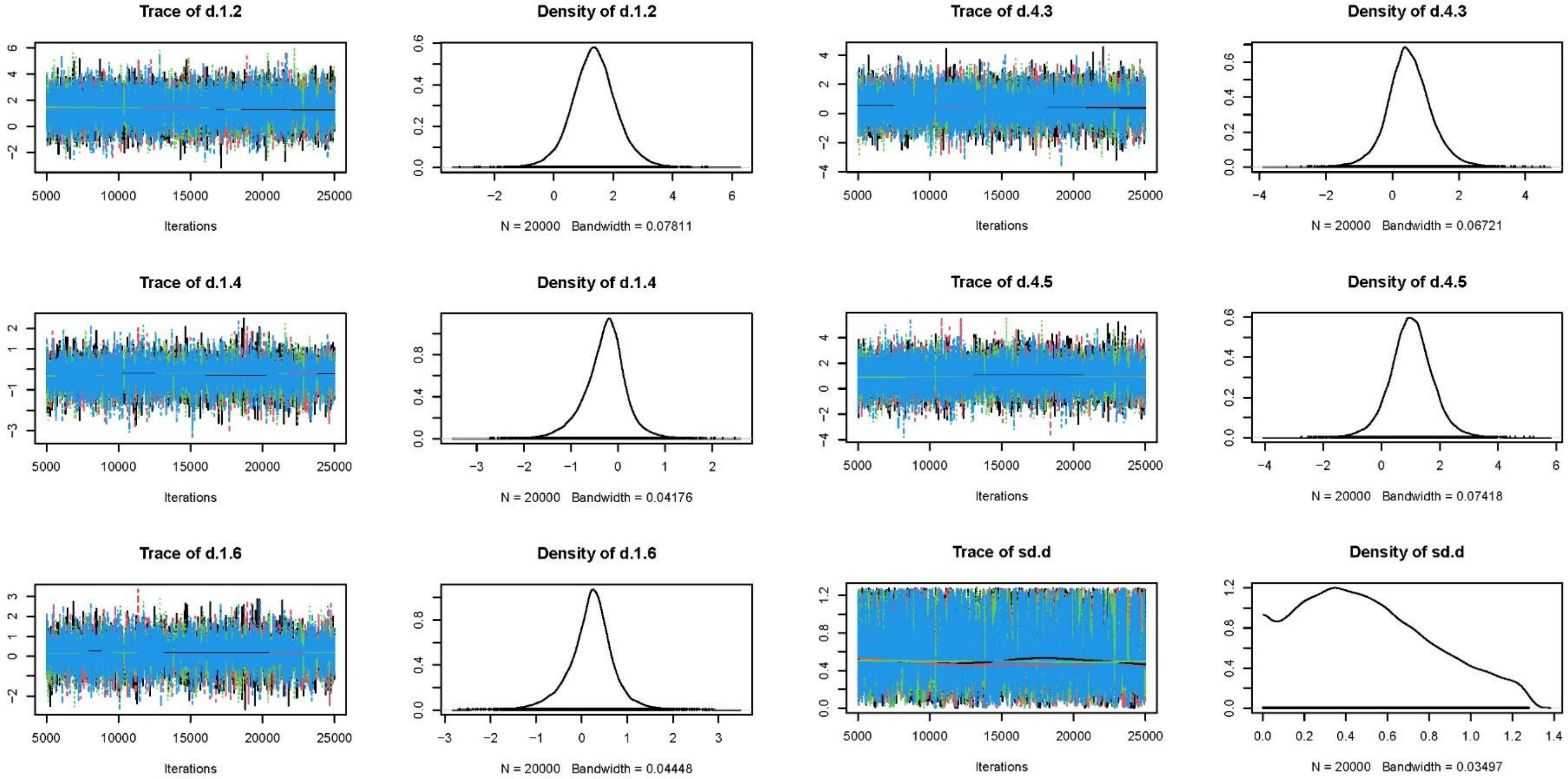

J: Brooks-Gelman-Rubin diagnostic for Grade 3 or higher AEs (1=Chemo-anti-EGFR, 2=Chemo-anti-EGFR/BRAF, 3=Anti- EGFR/MEK, 4=Anti-EGFR/BRAF, 5=Anti-EGFR/BRAF/PI3K, 6=Anti-EGFR/BRAF/MEK)

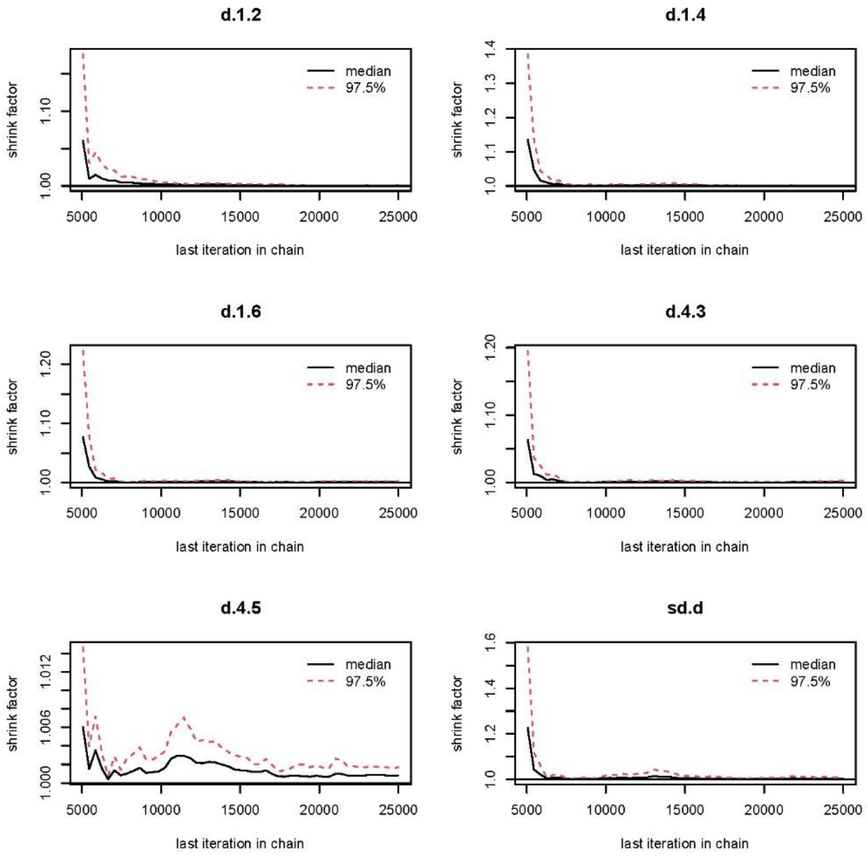

Figure S3. Pooled efficacy and safety estimates (objective response rate, ORR; disease control rate, DCR; grade-3 or higher AE) of the first-line regimen in single-arm meta-analysis.

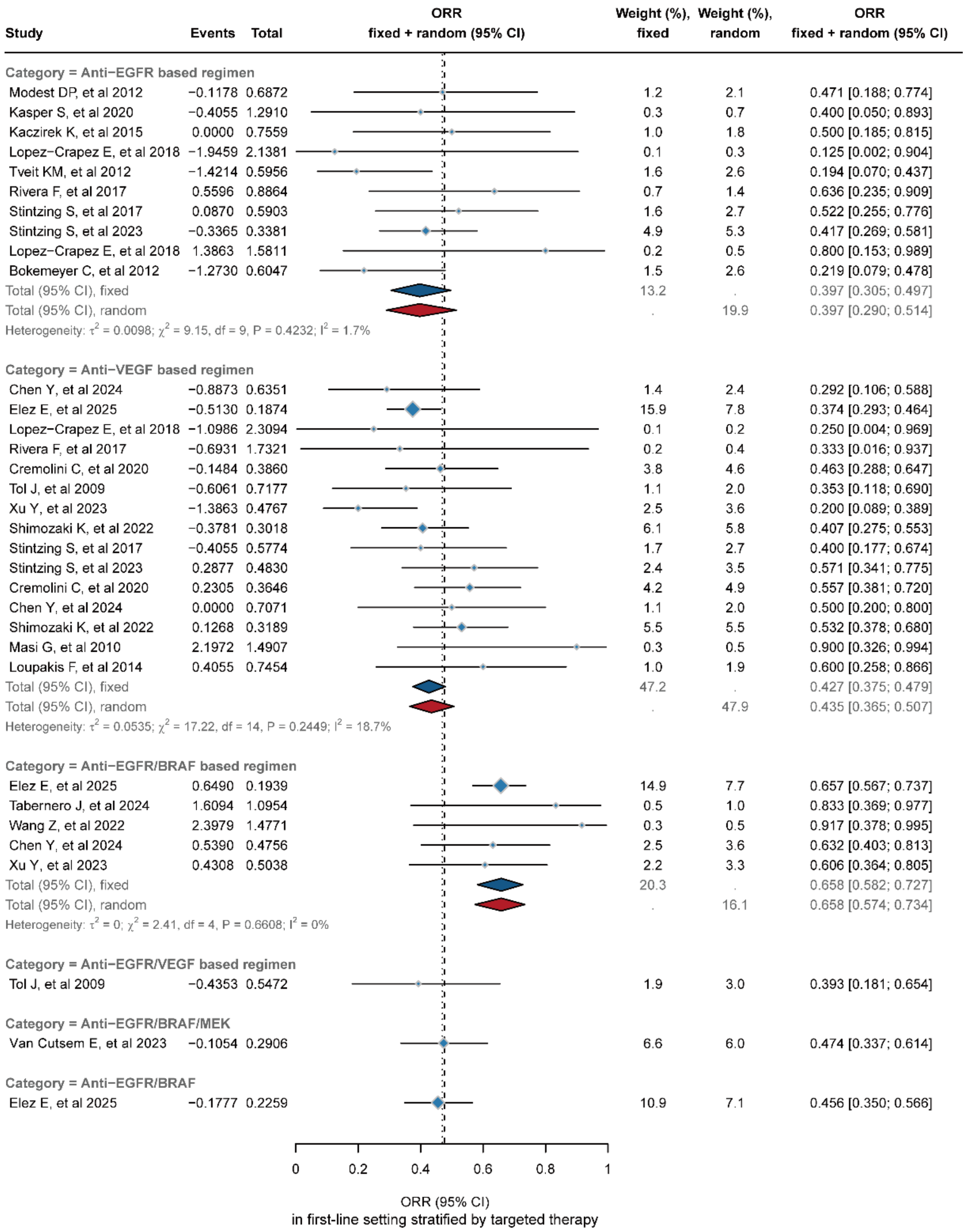

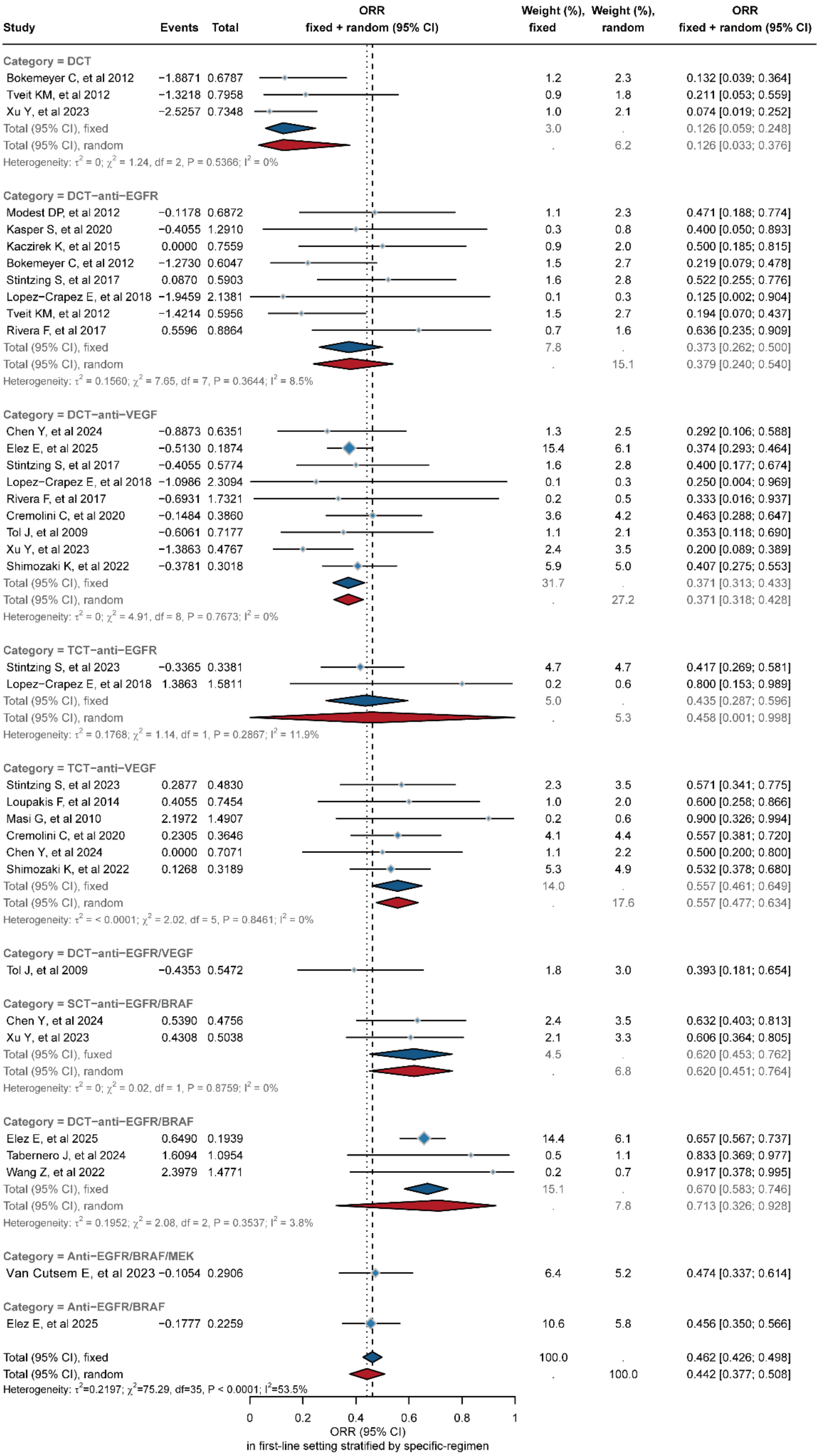
Study Events Total
ORR fixed + random (95% CI)
Weight (%), fixed
Weight (%), random
ORR fixed + random (95% CI)
Category = DCT
Bokemeyer C, et al 2012 −1.8871 0.6787 1.2 2.3 0.132 [0.039; 0.364]
Tveit KM, et al 2012 −1.3218 0.7958 0.9 1.8 0.211 [0.053; 0.559]
Xu Y, et al 2023 −2.5257 0.7348 1.0 2.1 0.074 [0.019; 0.252]
Total (95% CI), fixed 3.0 . 0.126 [0.059; 0.248]
Total (95% CI), random . 6.2 0.126 [0.033; 0.376]
Heterogeneity: τ^2 = 0; χ^2 = 1.24, df = 2, P = 0.5366; I^2 = 0%
Category = DCT−anti−EGFR
Modest DP, et al 2012 −0.1178 0.6872 1.1 2.3 0.471 [0.188; 0.774]
Kasper S, et al 2020 −0.4055 1.2910 0.3 0.8 0.400 [0.050; 0.893]
Kaczirek K, et al 2015 0.0000 0.7559 0.9 2.0 0.500 [0.185; 0.815]
Bokemeyer C, et al 2012 −1.2730 0.6047 1.5 2.7 0.219 [0.079; 0.478]
Stintzing S, et al 2017 0.0870 0.5903 1.6 2.8 0.522 [0.255; 0.776]
Lopez−Crapez E, et al 2018 −1.9459 2.1381 0.1 0.3 0.125 [0.002; 0.904]
Tveit KM, et al 2012 −1.4214 0.5956 1.5 2.7 0.194 [0.070; 0.437]
Rivera F, et al 2017 0.5596 0.8864 0.7 1.6 0.636 [0.235; 0.909]
Total (95% CI), fixed 7.8 . 0.373 [0.262; 0.500]
Total (95% CI), random . 15.1 0.379 [0.240; 0.540]
Heterogeneity: τ^2 = 0.1560; χ^2 = 7.65, df = 7, P = 0.3644; I^2 = 8.5%
Category = DCT−anti−VEGF
Chen Y, et al 2024 −0.8873 0.6351 1.3 2.5 0.292 [0.106; 0.588]
Elez E, et al 2025 −0.5130 0.1874 15.4 6.1 0.374 [0.293; 0.464]
Stintzing S, et al 2017 −0.4055 0.5774 1.6 2.8 0.400 [0.177; 0.674]
Lopez−Crapez E, et al 2018 −1.0986 2.3094 0.1 0.3 0.250 [0.004; 0.969]
Rivera F, et al 2017 −0.6931 1.7321 0.2 0.5 0.333 [0.016; 0.937]
Cremolini C, et al 2020 −0.1484 0.3860 3.6 4.2 0.463 [0.288; 0.647]
Tol J, et al 2009 −0.6061 0.7177 1.1 2.1 0.353 [0.118; 0.690]
Xu Y, et al 2023 −1.3863 0.4767 2.4 3.5 0.200 [0.089; 0.389]
Shimozaki K, et al 2022 −0.3781 0.3018 5.9 5.0 0.407 [0.275; 0.553]
Total (95% CI), fixed 31.7 . 0.371 [0.313; 0.433]
Total (95% CI), random . 27.2 0.371 [0.318; 0.428]
Heterogeneity: τ^2 = 0; χ^2 = 4.91, df = 8, P = 0.7673; I^2 = 0%
Category = TCT−anti−EGFR
Stintzing S, et al 2023 −0.3365 0.3381 4.7 4.7 0.417 [0.269; 0.581]
Lopez−Crapez E, et al 2018 1.3863 1.5811 0.2 0.6 0.800 [0.153; 0.989]
Total (95% CI), fixed 5.0 . 0.435 [0.287; 0.596]
Total (95% CI), random . 5.3 0.458 [0.001; 0.998]
Heterogeneity: τ^2 = 0.1768; χ^2 = 1.14, df = 1, P = 0.2867; I^2 = 11.9%
Category = TCT−anti−VEGF
Stintzing S, et al 2023 0.2877 0.4830 2.3 3.5 0.571 [0.341; 0.775]
Loupakis F, et al 2014 0.4055 0.7454 1.0 2.0 0.600 [0.258; 0.866]
Masi G, et al 2010 2.1972 1.4907 0.2 0.6 0.900 [0.326; 0.994]
Cremolini C, et al 2020 0.2305 0.3646 4.1 4.4 0.557 [0.381; 0.720]
Chen Y, et al 2024 0.0000 0.7071 1.1 2.2 0.500 [0.200; 0.800]
Shimozaki K, et al 2022 0.1268 0.3189 5.3 4.9 0.532 [0.378; 0.680]
Total (95% CI), fixed 14.0 . 0.557 [0.461; 0.649]
Total (95% CI), random . 17.6 0.557 [0.477; 0.634]
Heterogeneity: τ^2 = < 0.0001; χ^2 = 2.02, df = 5, P = 0.8461; I^2 = 0%
Category = DCT−anti−EGFR/VEGF
Tol J, et al 2009 −0.4353 0.5472 1.8 3.0 0.393 [0.181; 0.654]
Category = SCT−anti−EGFR/BRAF
Chen Y, et al 2024 0.5390 0.4756 2.4 3.5 0.632 [0.403; 0.813]
Xu Y, et al 2023 0.4308 0.5038 2.1 3.3 0.606 [0.364; 0.805]
Total (95% CI), fuxed 4.5 . 0.620 [0.453; 0.762]
Total (95% CI), random . 6.8 0.620 [0.451; 0.764]
Heterogeneity: τ^2 = 0; χ^2 = 0.02, df = 1, P = 0.8759; I^2 = 0%
Category = DCT−anti−EGFR/BRAF
Elez E, et al 2025 0.6490 0.1939 14.4 6.1 0.657 [0.567; 0.737]
Tabernero J, et al 2024 1.6094 1.0954 0.5 1.1 0.833 [0.369; 0.977]
Wang Z, et al 2022 2.3979 1.4771 0.2 0.7 0.917 [0.378; 0.995]
Total (95% CI), fixed 15.1 . 0.670 [0.583; 0.746]
Total (95% CI), random . 7.8 0.713 [0.326; 0.928]
Heterogeneity: τ^2 = 0.1952; χ^2 = 2.08, df = 2, P = 0.3537; I^2 = 3.8%
Category = Anti−EGFR/BRAF/MEK
Van Cutsem E, et al 2023 −0.1054 0.2906 6.4 5.2 0.474 [0.337; 0.614]
Category = Anti−EGFR/BRAF
Elez E, et al 2025 −0.1777 0.2259 10.6 5.8 0.456 [0.350; 0.566]
Total (95% CI), fixed 100.0 . 0.462 [0.426; 0.498]
Total (95% CI), random . 100.0 0.442 [0.377; 0.508]
Heterogeneity: τ^2=0.2197; χ^2=75.29, df=35, P < 0.0001; I^2=53.5%
0 0.2 0.4 0.6 0.8 1
ORR (95% CI)
in first-line setting stratified by specific-regimen

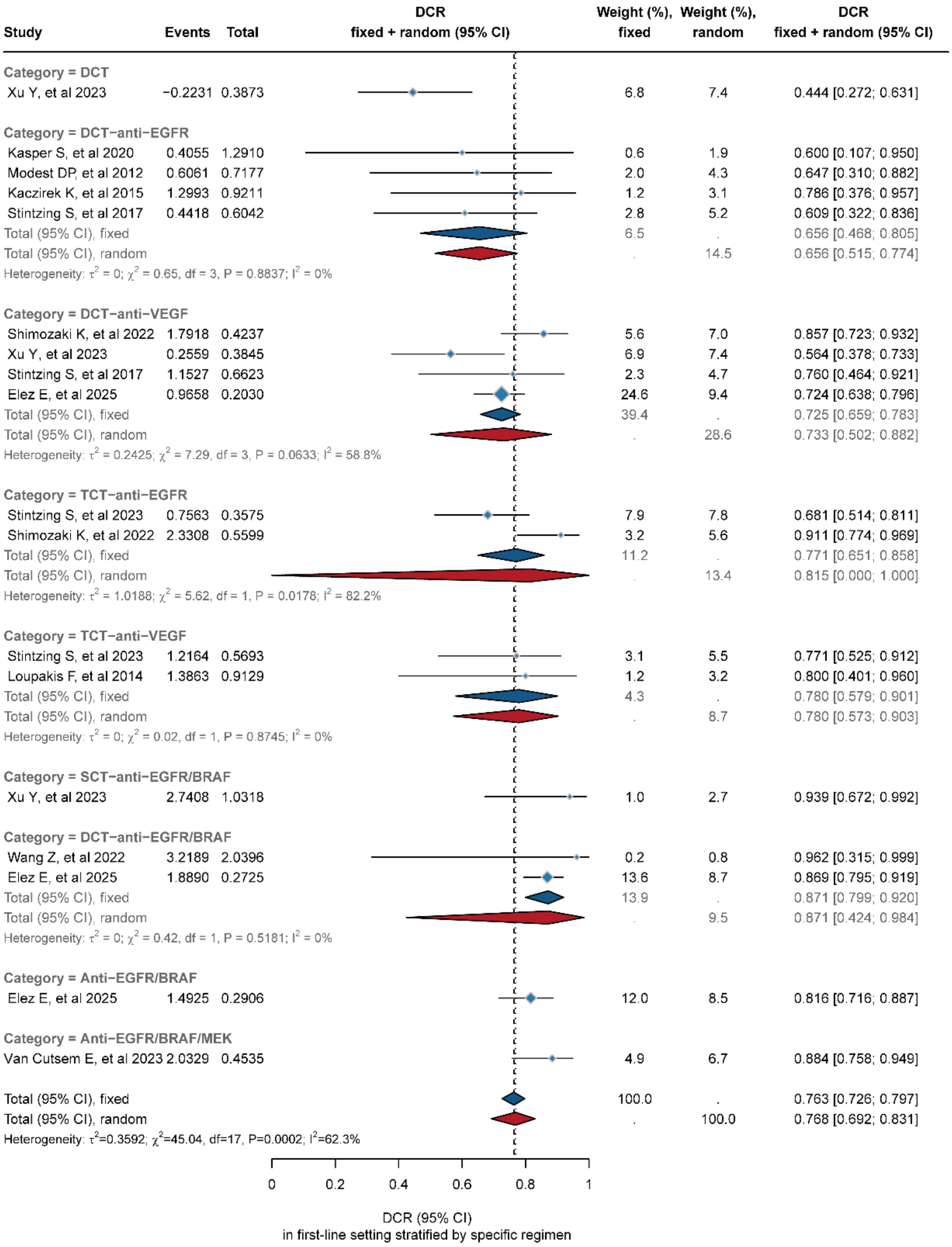
Study
Events
Total
DCR fixed + random (95% CI)
Weight (%), fixed
Weight (%), random
DCR fixed + random (95% CI)
Category = DCT
Xu Y, et al 2023 −0.2231 0.3873 6.8 7.4 0.444 [0.272; 0.631]
Category = DCT−anti−EGFR
Kasper S, et al 2020 0.4055 1.2910 0.6 1.9 0.600 [0.107; 0.950]
Modest DP, et al 2012 0.6061 0.7177 2.0 4.3 0.647 [0.310; 0.882]
Kaczirek K, et al 2015 1.2993 0.9211 1.2 3.1 0.786 [0.376; 0.957]
Stintzing S, et al 2017 0.4418 0.6042 2.8 5.2 0.609 [0.322; 0.836]
Total (95% CI), fixed 6.5 . 0.656 [0.468; 0.805]
Total (95% CI), random . 14.5 0.656 [0.515; 0.774]
Heterogeneity: τ2 = 0; χ2 = 0.65, df = 3, P = 0.8837; I2 = 0%
Category = DCT−anti−VEGF
Shimozaki K, et al 2022 1.7918 0.4237 5.6 7.0 0.857 [0.723; 0.932]
Xu Y, et al 2023 0.2559 0.3845 6.9 7.4 0.564 [0.378; 0.733]
Stintzing S, et al 2017 1.1527 0.6623 2.3 4.7 0.760 [0.464; 0.921]
Elez E, et al 2025 0.9658 0.2030 24.6 9.4 0.724 [0.638; 0.796]
Total (95% CI), fixed 39.4 . 0.725 [0.659; 0.783]
Total (95% CI), random . 28.6 0.733 [0.502; 0.882]
Heterogeneity: τ2 = 0.2425; χ2 = 7.29, df = 3, P = 0.0633; I2 = 58.8%
Category = TCT−anti−EGFR
Stintzing S, et al 2023 0.7563 0.3575 7.9 7.8 0.681 [0.514; 0.811]
Shimozaki K, et al 2022 2.3308 0.5599 3.2 5.6 0.911 [0.774; 0.969]
Total (95% CI), fixed 11.2 . 0.771 [0.651; 0.858]
Total (95% CI), random . 13.4 0.815 [0.000; 1.000]
Heterogeneity: τ2 = 1.0188; χ2 = 5.62, df = 1, P = 0.0178; I2 = 82.2%
Category = TCT−anti−VEGF
Stintzing S, et al 2023 1.2164 0.5693 3.1 5.5 0.771 [0.525; 0.912]
Loupakis F, et al 2014 1.3863 0.9129 1.2 3.2 0.800 [0.401; 0.960]
Total (95% CI), fixed 4.3 . 0.780 [0.579; 0.901]
Total (95% CI), random . 8.7 0.780 [0.573; 0.903]
Heterogeneity: τ2 = 0; χ2 = 0.02, df = 1, P = 0.8745; I2 = 0%
Category = SCT−anti−EGFR/BRAF
Xu Y, et al 2023 2.7408 1.0318 1.0 2.7 0.939 [0.672; 0.992]
Category = DCT−anti−EGFR/BRAF
Wang Z, et al 2022 3.2189 2.0396 0.2 0.8 0.962 [0.315; 0.999]
Elez E, et al 2025 1.8890 0.2725 13.6 8.7 0.869 [0.795; 0.919]
Total (95% CI), fixed 13.9 . 0.871 [0.799; 0.920]
Total (95% CI), random . 9.5 0.871 [0.424; 0.984]
Heterogeneity: τ2 = 0; χ2 = 0.42, df = 1, P = 0.5181; I2 = 0%
Category = Anti−EGFR/BRAF
Elez E, et al 2025 1.4925 0.2906 12.0 8.5 0.816 [0.716; 0.887]
Category = Anti−EGFR/BRAF/MEK
Van Cutsem E, et al 2023 2.0329 0.4535 4.9 6.7 0.884 [0.758; 0.949]
Total (95% CI), fixed 100.0 . 0.763 [0.726; 0.797]
Total (95% CI), random . 100.0 0.768 [0.692; 0.831]
Heterogeneity: τ2=0.3592; χ2=45.04, df=17, P=0.0002; I2=62.3%
0 0.2 0.4 0.6 0.8 1
DCR (95% CI)
in first-line setting stratified by specific regimen

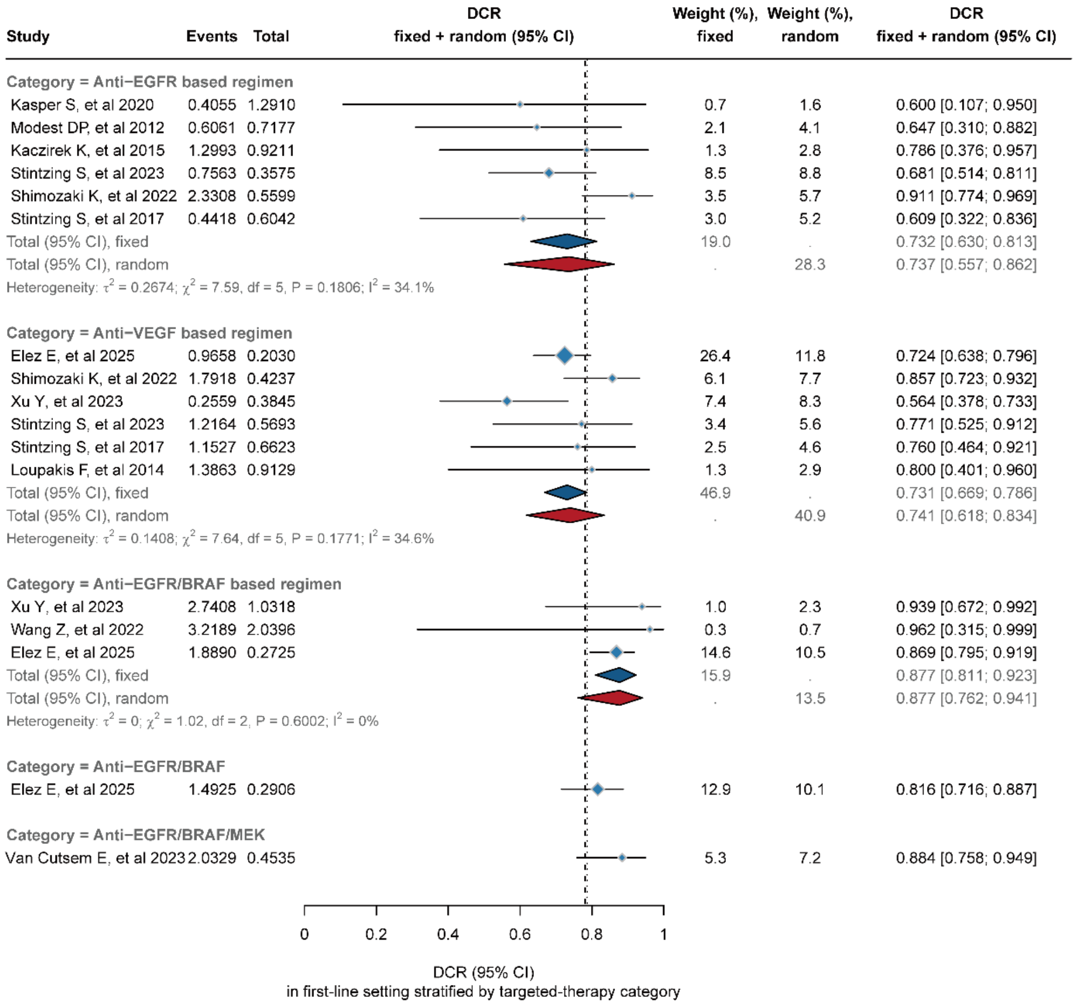
Study Events Total
DCR fixed + random (95% CI)
Weight (%), fixed
Weight (%), random
DCR fixed + random (95% CI)
Category = Anti−EGFR based regimen
Kasper S, et al 2020 0.4055 1.2910 0.7 1.6 0.600 [0.107; 0.950]
Modest DP, et al 2012 0.6061 0.7177 2.1 4.1 0.647 [0.310; 0.882]
Kaczirek K, et al 2015 1.2993 0.9211 1.3 2.8 0.786 [0.376; 0.957]
Stintzing S, et al 2023 0.7563 0.3575 8.5 8.8 0.681 [0.514; 0.811]
Shimozaki K, et al 2022 2.3308 0.5599 3.5 5.7 0.911 [0.774; 0.969]
Stintzing S, et al 2017 0.4418 0.6042 3.0 5.2 0.609 [0.322; 0.836]
Total (95% CI), fixed 19.0 . 0.732 [0.630; 0.813]
Total (95% CI), random . 28.3 0.737 [0.557; 0.862]
Heterogeneity: τ^2 = 0.2674; χ^2 = 7.59, df = 5, P = 0.1806; I^2 = 34.1%
Category = Anti−VEGF based regimen
Elez E, et al 2025 0.9658 0.2030 26.4 11.8 0.724 [0.638; 0.796]
Shimozaki K, et al 2022 1.7918 0.4237 6.1 7.7 0.857 [0.723; 0.932]
Xu Y, et al 2023 0.2559 0.3845 7.4 8.3 0.564 [0.378; 0.733]
Stintzing S, et al 2023 1.2164 0.5693 3.4 5.6 0.771 [0.525; 0.912]
Stintzing S, et al 2017 1.1527 0.6623 2.5 4.6 0.760 [0.464; 0.921]
Loupakis F, et al 2014 1.3863 0.9129 1.3 2.9 0.800 [0.401; 0.960]
Total (95% CI), fixed 46.9 . 0.731 [0.669; 0.786]
Total (95% CI), random . 40.9 0.741 [0.618; 0.834]
Heterogeneity: τ^2 = 0.1408; χ^2 = 7.64, df = 5, P = 0.1771; I^2 = 34.6%
Category = Anti−EGFR/BRAF based regimen
Xu Y, et al 2023 2.7408 1.0318 1.0 2.3 0.939 [0.672; 0.992]
Wang Z, et al 2022 3.2189 2.0396 0.3 0.7 0.962 [0.315; 0.999]
Elez E, et al 2025 1.8890 0.2725 14.6 10.5 0.869 [0.795; 0.919]
Total (95% CI), fixed 15.9 . 0.877 [0.811; 0.923]
Total (95% CI), random . 13.5 0.877 [0.762; 0.941]
Heterogeneity: τ^2 = 0; χ^2 = 1.02, df = 2, P = 0.6002; I^2 = 0%
Category = Anti−EGFR/BRAF
Elez E, et al 2025 1.4925 0.2906 12.9 10.1 0.816 [0.716; 0.887]
Category = Anti−EGFR/BRAF/MEK
Van Cutsem E, et al 2023 2.0329 0.4535 5.3 7.2 0.884 [0.758; 0.949]
0 0.2 0.4 0.6 0.8 1
DCR (95% CI)
in first-line setting stratified by targeted-therapy category

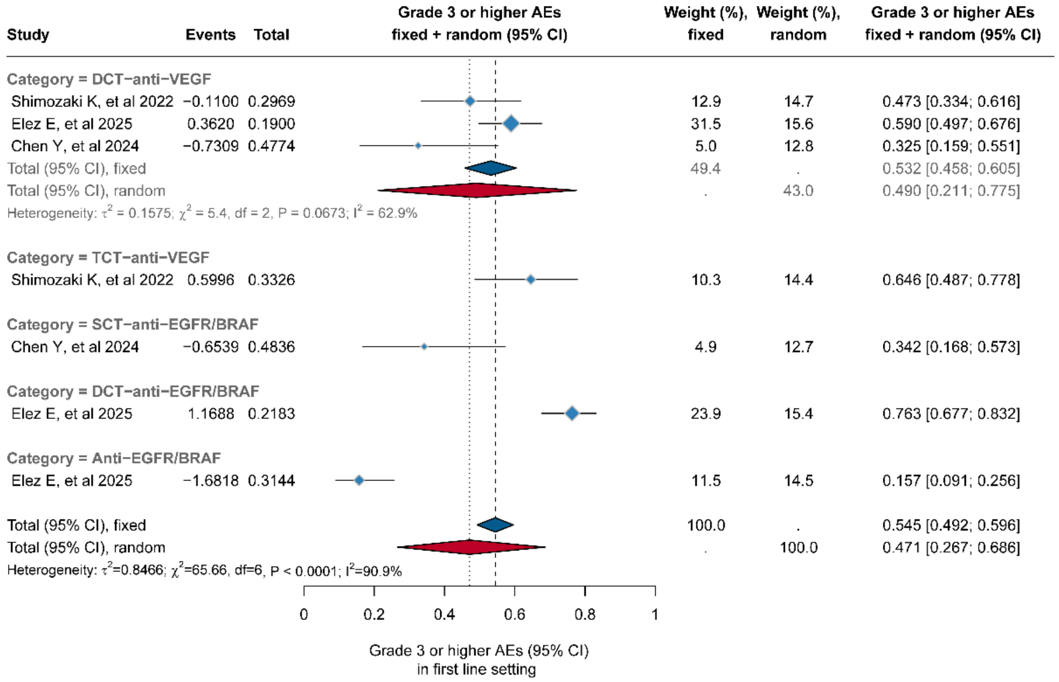
Study
Events
Total
Grade 3 or higher AEs fixed + random (95% CI)
Weight (%), fixed
Weight (%), random
Grade 3 or higher AEs fixed + random (95% CI)
Category = DCT−anti−VEGF
Shimozaki K, et al 2022 −0.1100 0.2969 12.9 14.7 0.473 [0.334; 0.616]
Elez E, et al 2025 0.3620 0.1900 31.5 15.6 0.590 [0.497; 0.676]
Chen Y, et al 2024 −0.7309 0.4774 5.0 12.8 0.325 [0.159; 0.551]
Total (95% CI), fixed 49.4 . 0.532 [0.458; 0.605]
Total (95% CI), random . 43.0 0.490 [0.211; 0.775]
Heterogeneity: τ^2 = 0.1575; χ^2 = 5.4, df = 2, P = 0.0673; I^2 = 62.9%
Category = TCT−anti−VEGF
Shimozaki K, et al 2022 0.5996 0.3326 10.3 14.4 0.646 [0.487; 0.778]
Category = SCT−anti−EGFR/BRAF
Chen Y, et al 2024 −0.6539 0.4836 4.9 12.7 0.342 [0.168; 0.573]
Category = DCT−anti−EGFR/BRAF
Elez E, et al 2025 1.1688 0.2183 23.9 15.4 0.763 [0.677; 0.832]
Category = Anti−EGFR/BRAF
Elez E, et al 2025 −1.6818 0.3144 11.5 14.5 0.157 [0.091; 0.256]
Total (95% CI), fixed 100.0 . 0.545 [0.492; 0.596]
Total (95% CI), random . 100.0 0.471 [0.267; 0.686]
Heterogeneity: τ^2=0.8466; χ^2=65.66, df=6, P < 0.0001; I^2=90.9%
0 0.2 0.4 0.6 0.8 1
Grade 3 or higher AEs (95% CI)
in first line setting

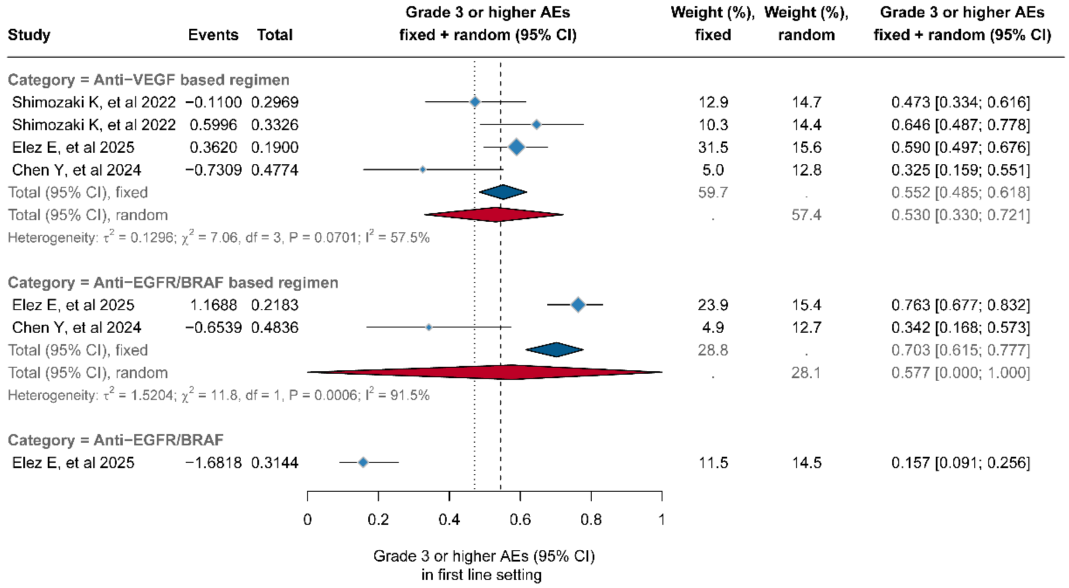
Study
Events
Total
Grade 3 or higher AEs fixed + random (95% CI)
Weight (%), fixed
Weight (%), random
Grade 3 or higher AEs fixed + random (95% CI)
Category = Anti−VEGF based regimen
Shimozaki K, et al 2022 −0.1100 0.2969 12.9 14.7 0.473 [0.334; 0.616]
Shimozaki K, et al 2022 0.5996 0.3326 10.3 14.4 0.646 [0.487; 0.778]
Elez E, et al 2025 0.3620 0.1900 31.5 15.6 0.590 [0.497; 0.676]
Chen Y, et al 2024 −0.7309 0.4774 5.0 12.8 0.325 [0.159; 0.551]
Total (95% CI), fixed 59.7 . 0.552 [0.485; 0.618]
Total (95% CI), random . 57.4 0.530 [0.330; 0.721]
Heterogeneity: τ^2 = 0.1296; χ^2 = 7.06, df = 3, P = 0.0701; I^2 = 57.5%
Category = Anti−EGFR/BRAF based regimen
Elez E, et al 2025 1.1688 0.2183 23.9 15.4 0.763 [0.677; 0.832]
Chen Y, et al 2024 −0.6539 0.4836 4.9 12.7 0.342 [0.168; 0.573]
Total (95% CI), fixed 28.8 . 0.703 [0.615; 0.777]
Total (95% CI), random . 28.1 0.577 [0.000; 1.000]
Heterogeneity: τ^2 = 1.5204; χ^2 = 11.8, df = 1, P = 0.0006; I^2 = 91.5%
Category = Anti−EGFR/BRAF
Elez E, et al 2025 −1.6818 0.3144 11.5 14.5 0.157 [0.091; 0.256]
0 0.2 0.4 0.6 0.8 1
Grade 3 or higher AEs (95% CI)
in first line setting

Figure S4. Pooled efficacy estimates (overall survival, OS; progression-free survival, PFS; objective response rate, ORR) of the first-line regimen in head-to-head comparisons in pairwise meta-analysis.

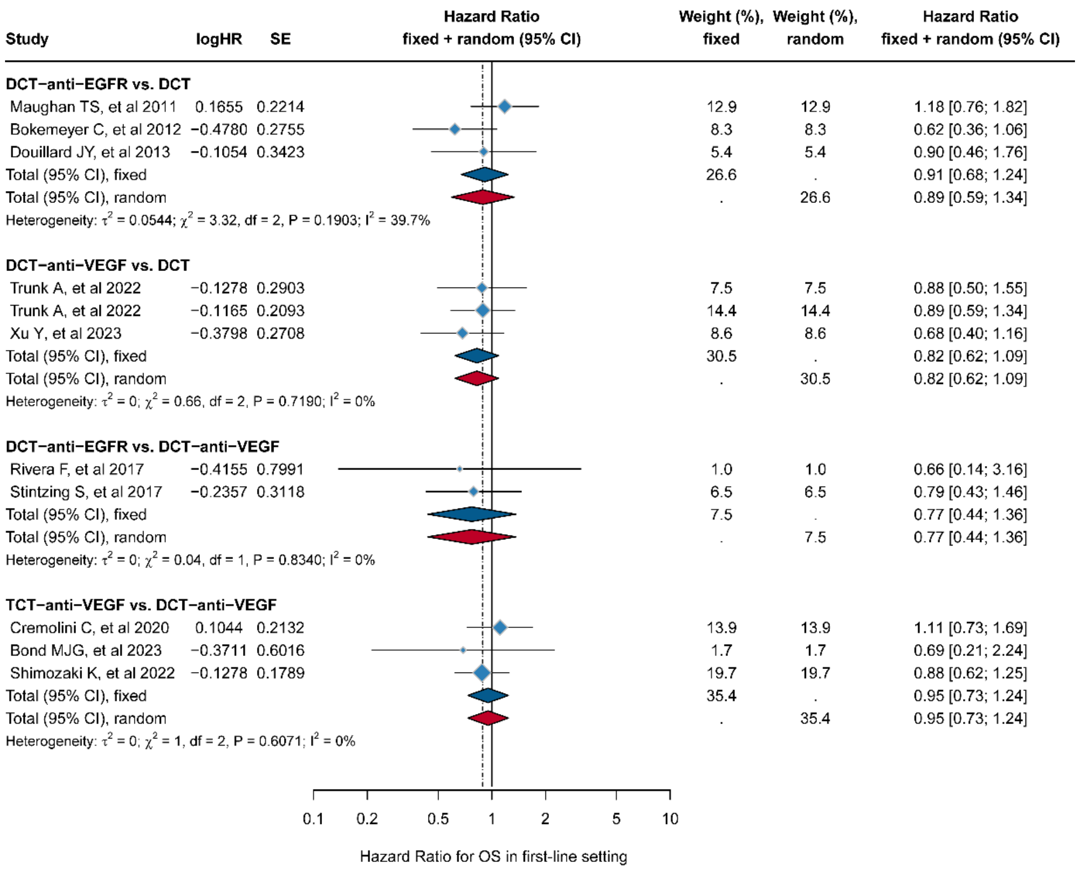

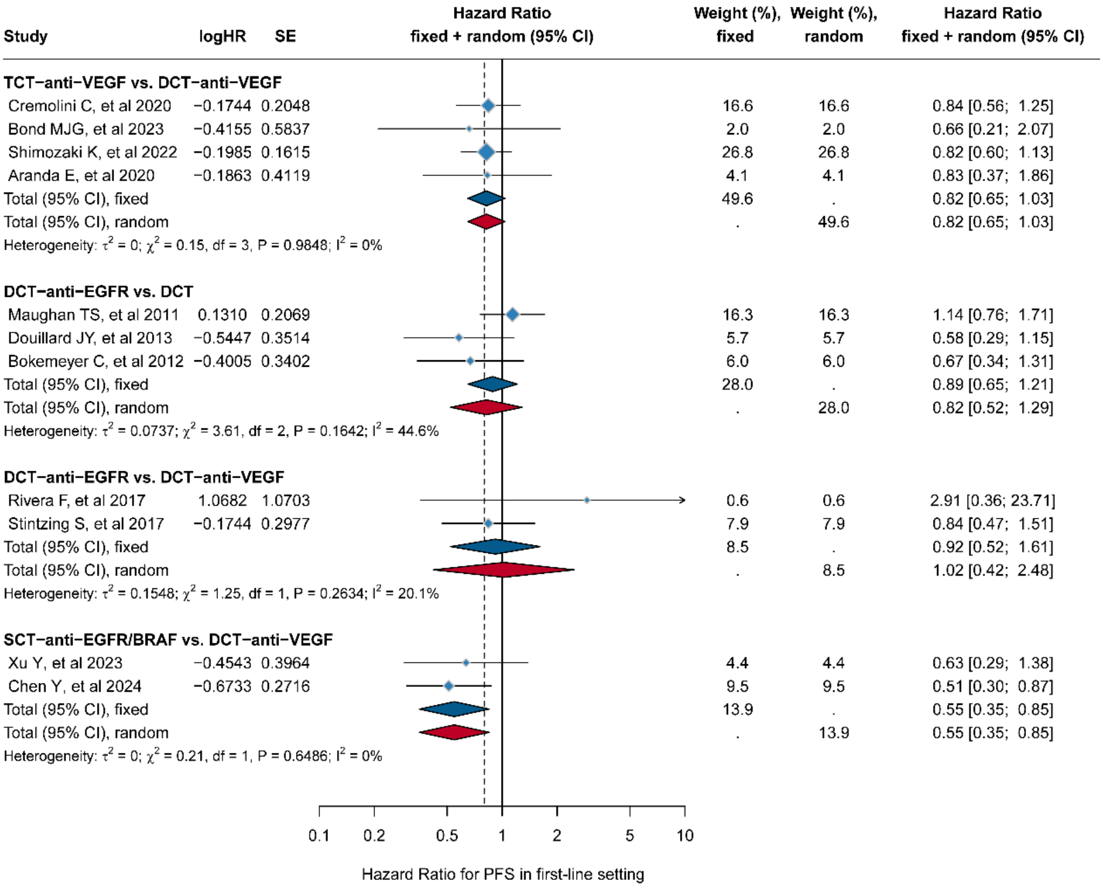
Study
logHR
SE
Hazard Ratio fixed + random (95% CI)
Weight (%), fixed
Weight (%), random
Hazard Ratio fixed + random (95% CI)
TCT−anti−VEGF vs. DCT−anti−VEGF
Cremolini C, et al 2020 −0.1744 0.2048 16.6 16.6 0.84 [0.56; 1.25]
Bond MJG, et al 2023 −0.4155 0.5837 2.0 2.0 0.66 [0.21; 2.07]
Shimozaki K, et al 2022 −0.1985 0.1615 26.8 26.8 0.82 [0.60; 1.13]
Aranda E, et al 2020 −0.1863 0.4119 4.1 4.1 0.83 [0.37; 1.86]
Total (95% CI), fixed 49.6 . 0.82 [0.65; 1.03]
Total (95% CI), random . 49.6 0.82 [0.65; 1.03]
Heterogeneity: τ^2 = 0; χ^2 = 0.15, df = 3, P = 0.9848; I^2 = 0%
DCT−anti−EGFR vs. DCT
Maughan TS, et al 2011 0.1310 0.2069 16.3 16.3 1.14 [0.76; 1.71]
Douillard JY, et al 2013 −0.5447 0.3514 5.7 5.7 0.58 [0.29; 1.15]
Bokemeyer C, et al 2012 −0.4005 0.3402 6.0 6.0 0.67 [0.34; 1.31]
Total (95% CI), fixed 28.0 . 0.89 [0.65; 1.21]
Total (95% CI), random . 28.0 0.82 [0.52; 1.29]
Heterogeneity: τ^2 = 0.0737; χ^2 = 3.61, df = 2, P = 0.1642; I^2 = 44.6%
DCT−anti−EGFR vs. DCT−anti−VEGF
Rivera F, et al 2017 1.0682 1.0703 0.6 0.6 2.91 [0.36; 23.71]
Stintzing S, et al 2017 −0.1744 0.2977 7.9 7.9 0.84 [0.47; 1.51]
Total (95% CI), fixed 8.5 . 0.92 [0.52; 1.61]
Total (95% CI), random . 8.5 1.02 [0.42; 2.48]
Heterogeneity: τ^2 = 0.1548; χ^2 = 1.25, df = 1, P = 0.2634; I^2 = 20.1%
SCT−anti−EGFR/BRAF vs. DCT−anti−VEGF
Xu Y, et al 2023 −0.4543 0.3964 4.4 4.4 0.63 [0.29; 1.38]
Chen Y, et al 2024 −0.6733 0.2716 9.5 9.5 0.51 [0.30; 0.87]
Total (95% CI), fixed 13.9 . 0.55 [0.35; 0.85]
Total (95% CI), random . 13.9 0.55 [0.35; 0.85]
Heterogeneity: τ^2 = 0; χ^2 = 0.21, df = 1, P = 0.6486; I^2 = 0%
0.1 0.2 0.5 1 2 5 10
Hazard Ratio for PFS in first-line setting

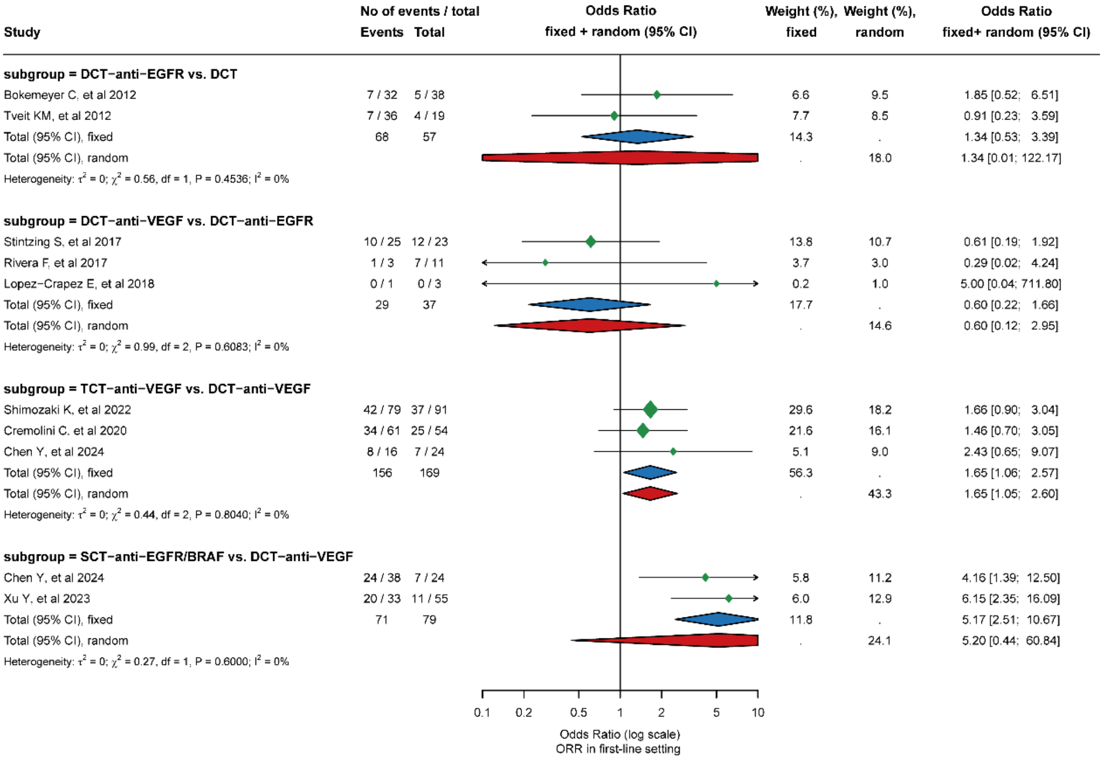
Study
No of events / total
Events Total
Odds Ratio fixed + random (95% CI)
Weight (%), fixed
Weight (%), random
Odds Ratio fixed+ random (95% CI)
subgroup = DCT−anti−EGFR vs. DCT
Bokemeyer C, et al 2012 7 / 32 5 / 38 6.6 9.5 1.85 [0.52; 6.51]
Tveit KM, et al 2012 7 / 36 4 / 19 7.7 8.5 0.91 [0.23; 3.59]
Total (95% CI), fixed 68 57 14.3 . 1.34 [0.53; 3.39]
Total (95% CI), random . 18.0 1.34 [0.01; 122.17]
Heterogeneity: τ^2 = 0; χ^2 = 0.56, df = 1, P = 0.4536; I^2 = 0%
subgroup = DCT−anti−VEGF vs. DCT−anti−EGFR
Stintzing S, et al 2017 10 / 25 12 / 23 13.8 10.7 0.61 [0.19; 1.92]
Rivera F, et al 2017 1 / 3 7 / 11 3.7 3.0 0.29 [0.02; 4.24]
Lopez−Crapez E, et al 2018 0 / 1 0 / 3 0.2 1.0 5.00 [0.04; 711.80]
Total (95% CI), fixed 29 37 17.7 . 0.60 [0.22; 1.66]
Total (95% CI), random . 14.6 0.60 [0.12; 2.95]
Heterogeneity: τ^2 = 0; χ^2 = 0.99, df = 2, P = 0.6083; I^2 = 0%
subgroup = TCT−anti−VEGF vs. DCT−anti−VEGF
Shimozaki K, et al 2022 42 / 79 37 / 91 29.6 18.2 1.66 [0.90; 3.04]
Cremolini C, et al 2020 34 / 61 25 / 54 21.6 16.1 1.46 [0.70; 3.05]
Chen Y, et al 2024 8 / 16 7 / 24 5.1 9.0 2.43 [0.65; 9.07]
Total (95% CI), fixed 156 169 56.3 . 1.65 [1.06; 2.57]
Total (95% CI), random . 43.3 1.65 [1.05; 2.60]
Heterogeneity: τ^2 = 0; χ^2 = 0.44, df = 2, P = 0.8040; I^2 = 0%
subgroup = SCT−anti−EGFR/BRAF vs. DCT−anti−VEGF
Chen Y, et al 2024 24 / 38 7 / 24 5.8 11.2 4.16 [1.39; 12.50]
Xu Y, et al 2023 20 / 33 11 / 55 6.0 12.9 6.15 [2.35; 16.09]
Total (95% CI), fixed 71 79 11.8 . 5.17 [2.51; 10.67]
Total (95% CI), random . 24.1 5.20 [0.44; 60.84]
Heterogeneity: τ^2 = 0; χ^2 = 0.27, df = 1, P = 0.6000; I^2 = 0%
0.1 0.2 0.5 1 2 5 10
Odds Ratio (log scale)
ORR in first-line setting

Figure S5. Eligible comparisons for disease control rate and grade 3 or higher AEs in the network meta-analysis.

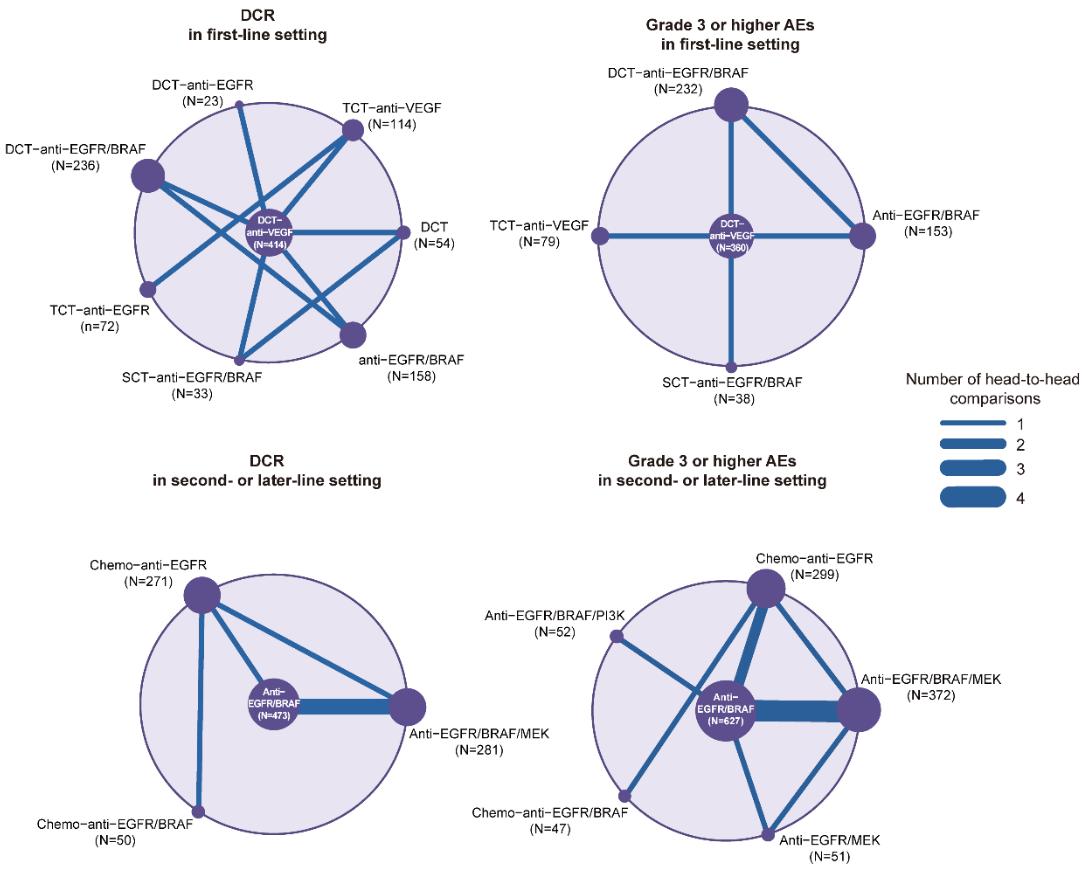

Figure S6: Pooled efficacy (objective response rate, ORR; disease control rate, DCR) and safety (grade 3 or higher AEs) estimates of multiple comparisons in first-line setting based on the network meta-analysis.

**ORR-Comparison**	DCT	DCT-anti-EGFR	DCT-anti-VEGF	DCT-anti-EGFR/VEGF	TCT-anti-EGFR	TCT-anti-VEGF	Anti-EGFR/BRAF	SCT-anti-EGFR/BRAF	DCT-anti-EGFR/BRAF
DCT	Reference	1.89 (0.54 to 6.35)	2.21 (0.57 to 8.79)	2.64 (0.25 to 31.4)	4.33 (0.82 to 103)	**4.74 (1.16 to 32.6)**	3.06 (0.37 to 26.9)	**12.1 (2.76 to 64.9)**	7.08 (0.84 to 62.6)
DCT-anti-EGFR	0.53 (0.16 to 1.86)	Reference	1.17 (0.35 to 4.23)	1.39 (0.14 to 15.9)	2.28 (0.48 to 51.2)	2.52 (0.68 to 16.0)	1.6 (0.22 to 13.4)	**6.32 (1.42 to 36.1)**	3.68 (0.51 to 31.9)
DCT-anti-VEGF	0.45 (0.11 to 1.74)	0.85 (0.24 to 2.89)	Reference	1.19 (0.17 to 9.25)	1.91 (0.54 to 33.9)	2.14 (1.00 to 7.86)	1.38 (0.26 to 7.36)	**5.47 (1.71 to 20.3)**	3.21 (0.61 to 17.8)
DCT-anti-EGFR/VEGF	0.38 (0.03 to 4.03)	0.72 (0.06 to 6.98)	0.84 (0.11 to 5.99)	Reference	1.63 (0.18 to 62.9)	1.82 (0.23 to 20.9)	1.16 (0.08 to 15.1)	4.61 (0.43 to 49.1)	2.69 (0.20 to 35.2)
TCT-anti-EGFR	0.23 (0.01 to 1.22)	0.44 (0.02 to 2.1)	0.52 (0.03 to 1.86)	0.61 (0.02 to 5.59)	Reference	1.13 (0.11 to 3.61)	0.75 (0.02 to 4.09)	2.81 (0.14 to 13.3)	1.75 (0.05 to 9.37)
TCT-anti-VEGF	**0.21 (0.03 to 0.86)**	0.40 (0.06 to 1.47)	0.47 (0.13 to 1.00)	0.55 (0.05 to 4.40)	0.88 (0.28 to 9.52)	Reference	0.66 (0.07 to 3.43)	2.51 (0.52 to 8.81)	1.53 (0.16 to 8.07)
Anti-EGFR/BRAF	0.33 (0.04 to 2.73)	0.62 (0.07 to 4.62)	0.72 (0.14 to 3.86)	0.86 (0.07 to 12.03)	1.33 (0.24 to 46.3)	1.51 (0.29 to 14.8)	Reference	3.95 (0.54 to 33.7)	2.33 (0.44 to 12.6)
SCT-anti-EGFR/BRAF	**0.08 (0.02 to 0.36)**	**0.16 (0.03 to 0.71)**	**0.18 (0.05 to 0.58)**	0.22 (0.02 to 2.32)	0.36 (0.07 to 6.92)	0.40 (0.11 to 1.93)	0.25 (0.03 to 1.84)	Reference	0.58 (0.07 to 4.29)
DCT-anti-EGFR/BRAF	0.14 (0.02 to 1.19)	0.27 (0.03 to 1.97)	0.31 (0.06 to 1.63)	0.37 (0.03 to 5.04)	0.57 (0.11 to 19.7)	0.65 (0.12 to 6.22)	0.43 (0.08 to 2.26)	1.72 (0.23 to 14.5)	Reference

**DCR-Comparison**	DCT	DCT-anti-EGFR	DCT-anti-VEGF	TCT-anti-EGFR	TCT-anti-VEGF	Anti-EGFR/BRAF	SCT-anti-EGFR/BRAF	DCT-anti-EGFR/BRAF
DCT	Reference	0.79 (0.01 to 115)	1.63 (0.05 to 53.7)	1.76 (0.01 to 818)	2.88 (0.02 to 412)	2.79 (0.02 to 391)	24.1 (0.59 to 1148)	4.14 (0.03 to 603)
DCT-anti-EGFR	1.26 (0.01 to 193)	Reference	2.09 (0.06 to 73.6)	2.29 (0.01 to 1052)	3.75 (0.02 to 560)	3.58 (0.02 to 533)	31.7 (0.17 to 5810)	5.32 (0.03 to 808)
DCT-anti-VEGF	0.61 (0.02 to 21.6)	0.48 (0.01 to 17.8)	Reference	1.09 (0.01 to 163)	1.76 (0.05 to 62.6)	1.71 (0.05 to 54.7)	14.7 (0.37 to 718)	2.54 (0.08 to 81.9)
TCT-anti-EGFR	0.57 (0.01 to 253)	0.44 (0.01 to 207)	0.92 (0.01 to 140)	Reference	1.63 (0.05 to 57.4)	1.57 (0.01 to 705)	13.9 (0.03 to 7832)	2.34 (0.01 to 1010)
TCT-anti-VEGF	0.35 (0.01 to 49.5)	0.27 (0.01 to 40.8)	0.57 (0.02 to 19.3)	0.62 (0.02 to 20.6)	Reference	0.97 (0.01 to 135)	8.42 (0.05 to 1552)	1.44 (0.01 to 197)
Anti-EGFR/BRAF	0.36 (0.01 to 51.9)	0.28 (0.01 to 42.6)	0.58 (0.02 to 18.9)	0.64 (0.01 to 272)	1.03 (0.01 to 147)	Reference	8.74 (0.05 to 1563)	1.49 (0.05 to 47.8)
SCT-anti-EGFR/BRAF	0.04 (0.01 to 1.71)	0.03 (0.01 to 5.73)	0.07 (0.01 to 2.73)	0.07 (0.01 to 35.8)	0.12 (0.01 to 20.2)	0.11 (0.01 to 18.2)	Reference	0.17 (0.01 to 28.27)
DCT-anti-EGFR/BRAF	0.24 (0.01 to 35.3)	0.19 (0.01 to 29.2)	0.39 (0.01 to 12.9)	0.43 (0.01 to 193)	0.70 (0.01 to 100)	0.67 (0.02 to 21.3)	5.84 (0.04 to 1037)	Reference

**Grade 3 or higher AEs-Comparison**	DCT-anti-VEGF	TCT-anti-VEGF	Anti-EGFR/BRAF	SCT-anti-EGFR/BRAF	DCT-anti-EGFR/BRAF
DCT-anti-VEGF	Reference	2.04 (0.06 to 79.2)	0.13 (0.01 to 4.54)	1.08 (0.03 to 41.7)	2.24 (0.06 to 78.0)
TCT-anti-VEGF	0.49 (0.01 to 17.6)	Reference	0.06 (0.01 to 9.69)	0.53 (0.01 to 88.9)	1.10 (0.01 to 170)
Anti-EGFR/BRAF	7.81 (0.22 to 300)	15.9 (0.10 to 2899)	Reference	8.55 (0.05 to 1500)	17.6 (0.48 to 654)
SCT-anti-EGFR/BRAF	0.93 (0.02 to 35.7)	1.89 (0.01 to 331)	0.12 (0.01 to 19.3)	Reference	2.07 (0.01 to 332)
DCT-anti-EGFR/BRAF	0.45 (0.01 to 16.1)	0.91 (0.01 to 163)	0.06 (0.01 to 2.07)	0.48 (0.01 to 83.2)	Reference

Figure S7. Pooled efficacy and safety estimates (objective response rate, ORR; disease control rate, DCR; grade-3 or higher AE) of the second- or later-line setting in single-arm meta-analysis.

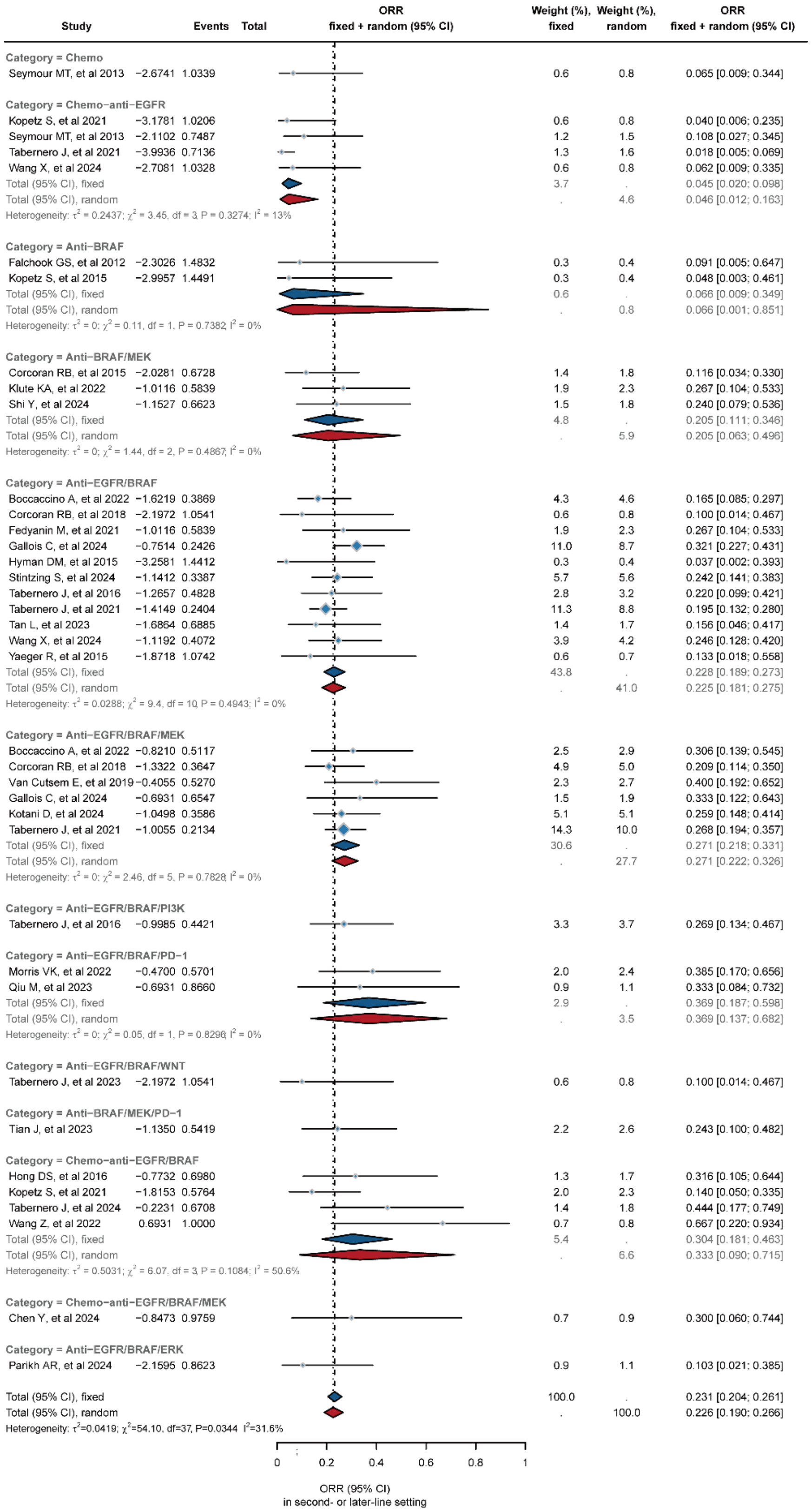

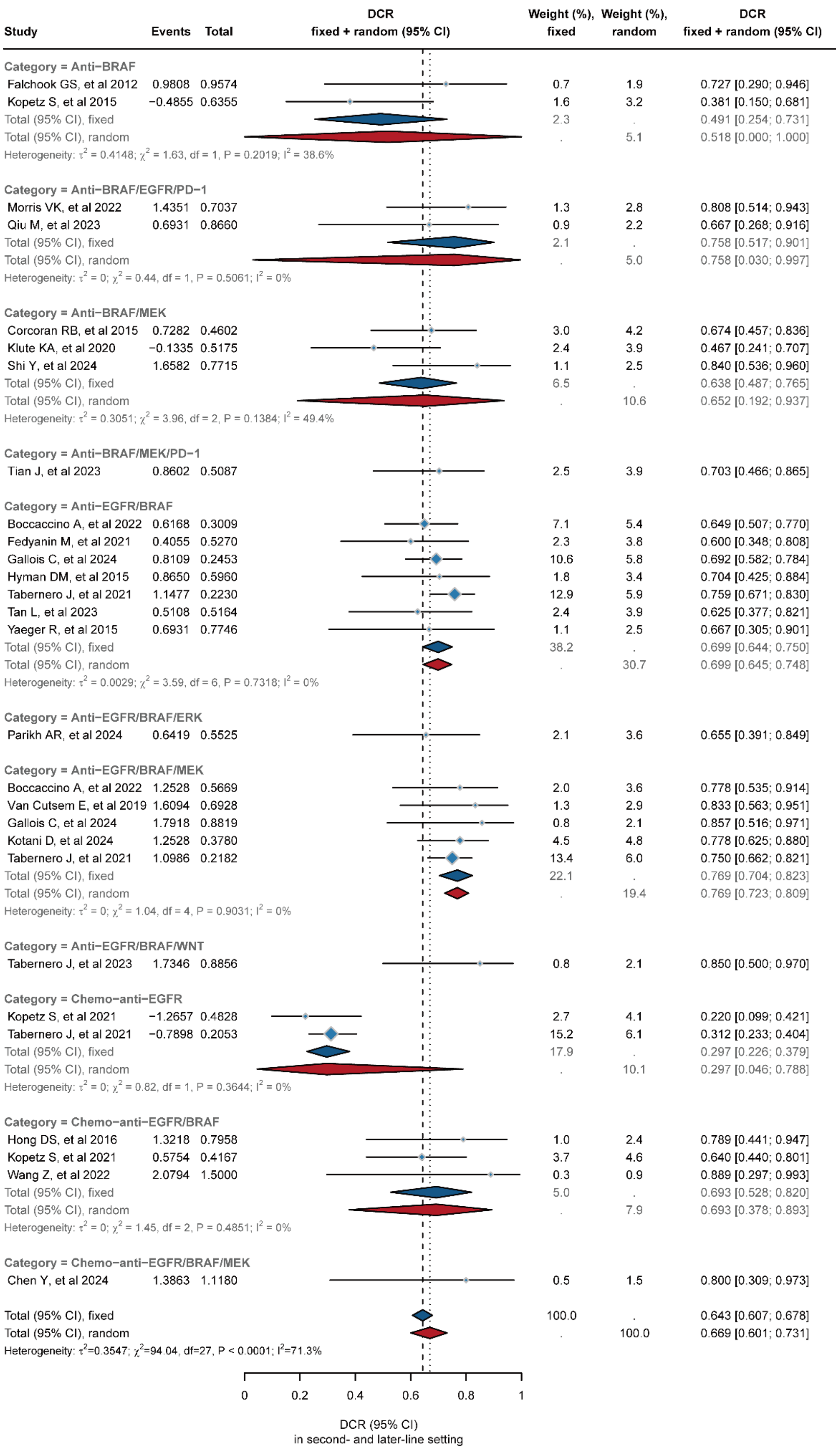
Study | Events | Total | DCR fixed + random (95% CI) | Weight (%), fixed | Weight (%), random | DCR fixed + random (95% CI)
Category = Anti−BRAF
Falchook GS, et al 2012 0.9808 0.9574 0.7 1.9 0.727 [0.290; 0.946]
Kopetz S, et al 2015 −0.4855 0.6355 1.6 3.2 0.381 [0.150; 0.681]
Total (95% CI), fixed 2.3 . 0.491 [0.254; 0.731]
Total (95% CI), random . 5.1 0.518 [0.000; 1.000]
Heterogeneity: τ^2 = 0.4148; χ^2 = 1.63, df = 1, P = 0.2019; I^2 = 38.6%
Category = Anti−BRAF/EGFR/PD−1
Morris VK, et al 2022 1.4351 0.7037 1.3 2.8 0.808 [0.514; 0.943]
Qiu M, et al 2023 0.6931 0.8660 0.9 2.2 0.667 [0.268; 0.916]
Total (95% CI), fixed 2.1 . 0.758 [0.517; 0.901]
Total (95% CI), random . 5.0 0.758 [0.030; 0.997]
Heterogeneity: τ^2 = 0; χ^2 = 0.44, df = 1, P = 0.5061; I^2 = 0%
Category = Anti−BRAF/MEK
Corcoran RB, et al 2015 0.7282 0.4602 3.0 4.2 0.674 [0.457; 0.836]
Klute KA, et al 2020 −0.1335 0.5175 2.4 3.9 0.467 [0.241; 0.707]
Shi Y, et al 2024 1.6582 0.7715 1.1 2.5 0.840 [0.536; 0.960]
Total (95% CI), fixed 6.5 . 0.638 [0.487; 0.765]
Total (95% CI), random . 10.6 0.652 [0.192; 0.937]
Heterogeneity: τ^2 = 0.3051; χ^2 = 3.96, df = 2, P = 0.1384; I^2 = 49.4%
Category = Anti−BRAF/MEK/PD−1
Tian J, et al 2023 0.8602 0.5087 2.5 3.9 0.703 [0.466; 0.865]
Category = Anti−EGFR/BRAF
Boccaccino A, et al 2022 0.6168 0.3009 7.1 5.4 0.649 [0.507; 0.770]
Fedyanin M, et al 2021 0.4055 0.5270 2.3 3.8 0.600 [0.348; 0.808]
Gallois C, et al 2024 0.8109 0.2453 10.6 5.8 0.692 [0.582; 0.784]
Hyman DM, et al 2015 0.8650 0.5960 1.8 3.4 0.704 [0.425; 0.884]
Tabernero J, et al 2021 1.1477 0.2230 12.9 5.9 0.759 [0.671; 0.830]
Tan L, et al 2023 0.5108 0.5164 2.4 3.9 0.625 [0.377; 0.821]
Yaeger R, et al 2015 0.6931 0.7746 1.1 2.5 0.667 [0.305; 0.901]
Total (95% CI), fixed 38.2 . 0.699 [0.644; 0.750]
Total (95% CI), random . 30.7 0.699 [0.645; 0.748]
Heterogeneity: τ^2 = 0.0029; χ^2 = 3.59, df = 6, P = 0.7318; I^2 = 0%
Category = Anti−EGFR/BRAF/ERK
Parikh AR, et al 2024 0.6419 0.5525 2.1 3.6 0.655 [0.391; 0.849]
Category = Anti−EGFR/BRAF/MEK
Boccaccino A, et al 2022 1.2528 0.5669 2.0 3.6 0.778 [0.535; 0.914]
Van Cutsem E, et al 2019 1.6094 0.6928 1.3 2.9 0.833 [0.563; 0.951]
Gallois C, et al 2024 1.7918 0.8819 0.8 2.1 0.857 [0.516; 0.971]
Kotani D, et al 2024 1.2528 0.3780 4.5 4.8 0.778 [0.625; 0.880]
Tabernero J, et al 2021 1.0986 0.2182 13.4 6.0 0.750 [0.662; 0.821]
Total (95% CI), fixed 22.1 . 0.769 [0.704; 0.823]
Total (95% CI), random . 19.4 0.769 [0.723; 0.809]
Heterogeneity: τ^2 = 0; χ^2 = 1.04, df = 4, P = 0.9031; I^2 = 0%
Category = Anti−EGFR/BRAF/WNT
Tabernero J, et al 2023 1.7346 0.8856 0.8 2.1 0.850 [0.500; 0.970]
Category = Chemo−anti−EGFR
Kopetz S, et al 2021 −1.2657 0.4828 2.7 4.1 0.220 [0.099; 0.421]
Tabernero J, et al 2021 −0.7898 0.2053 15.2 6.1 0.312 [0.233; 0.404]
Total (95% CI), fixed 17.9 . 0.297 [0.226; 0.379]
Total (95% CI), random . 10.1 0.297 [0.046; 0.788]
Heterogeneity: τ^2 = 0; χ^2 = 0.82, df = 1, P = 0.3644; I^2 = 0%
Category = Chemo−anti−EGFR/BRAF
Hong DS, et al 2016 1.3218 0.7958 1.0 2.4 0.789 [0.441; 0.947]
Kopetz S, et al 2021 0.5754 0.4167 3.7 4.6 0.640 [0.440; 0.801]
Wang Z, et al 2022 2.0794 1.5000 0.3 0.9 0.889 [0.297; 0.993]
Total (95% CI), fixed 5.0 . 0.693 [0.528; 0.820]
Total (95% CI), random . 7.9 0.693 [0.378; 0.893]
Heterogeneity: τ^2 = 0; χ^2 = 1.45, df = 2, P = 0.4851; I^2 = 0%
Category = Chemo−anti−EGFR/BRAF/MEK
Chen Y, et al 2024 1.3863 1.1180 0.5 1.5 0.800 [0.309; 0.973]
Total (95% CI), fixed 100.0 . 0.643 [0.607; 0.678]
Total (95% CI), random . 100.0 0.669 [0.601; 0.731]
Heterogeneity: τ^2=0.3547; χ^2=94.04, df=27, P < 0.0001; I^2=71.3%
0 0.2 0.4 0.6 0.8 1
DCR (95% CI)
in second- and later-line setting

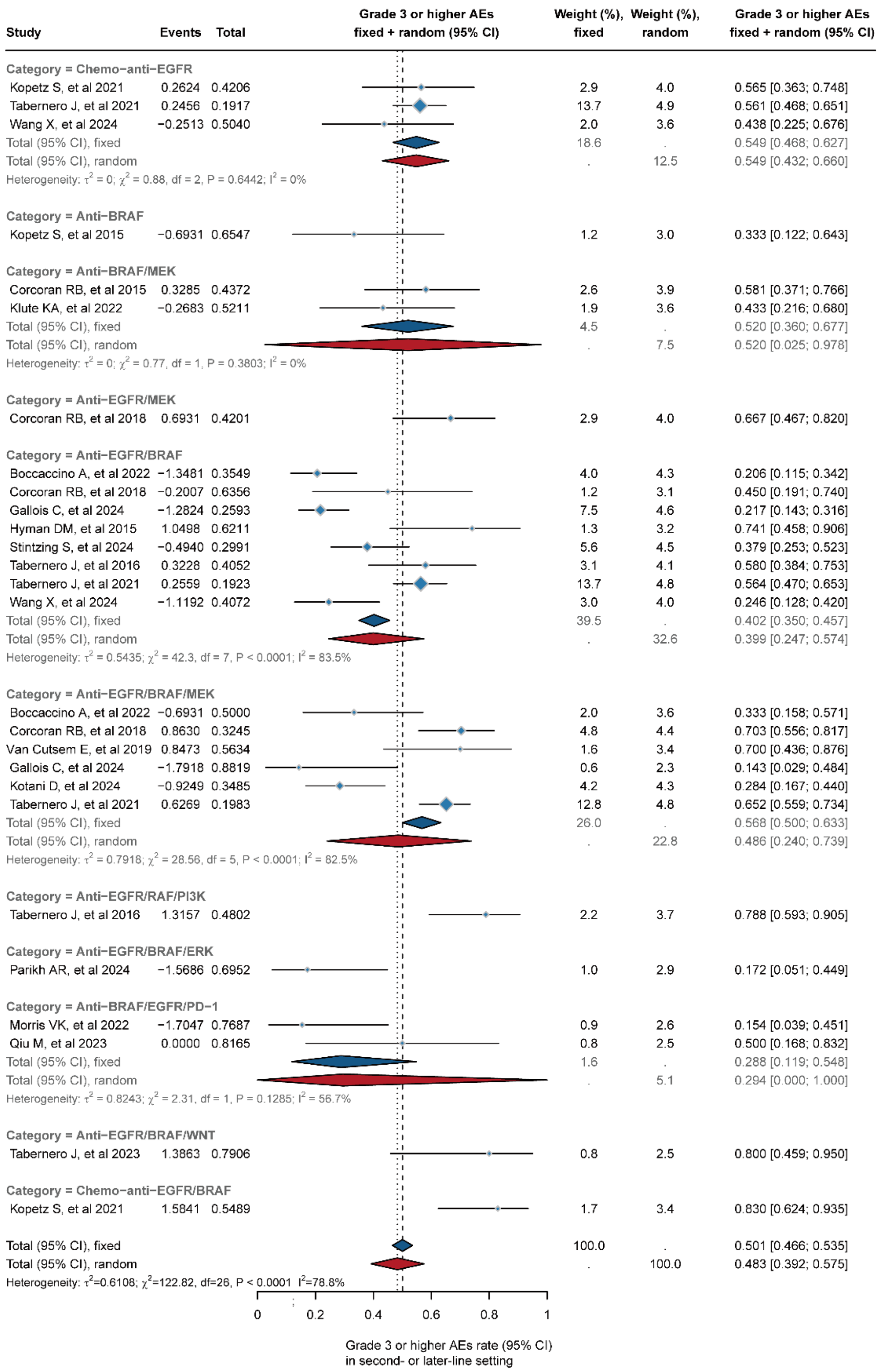
Study
Events
Total
Grade 3 or higher AEs fixed + random (95% CI)
Weight (%), fixed
Weight (%), random
Grade 3 or higher AEs fixed + random (95% CI)
Category = Chemo−anti−EGFR
Kopetz S, et al 2021 0.2624 0.4206 2.9 4.0 0.565 [0.363; 0.748]
Tabernero J, et al 2021 0.2456 0.1917 13.7 4.9 0.561 [0.468; 0.651]
Wang X, et al 2024 −0.2513 0.5040 2.0 3.6 0.438 [0.225; 0.676]
Total (95% CI), fixed 18.6 . 0.549 [0.468; 0.627]
Total (95% CI), random . 12.5 0.549 [0.432; 0.660]
Heterogeneity: τ^2 = 0; χ^2 = 0.88, df = 2, P = 0.6442; I^2 = 0%
Category = Anti−BRAF
Kopetz S, et al 2015 −0.6931 0.6547 1.2 3.0 0.333 [0.122; 0.643]
Category = Anti−BRAF/MEK
Corcoran RB, et al 2015 0.3285 0.4372 2.6 3.9 0.581 [0.371; 0.766]
Klute KA, et al 2022 −0.2683 0.5211 1.9 3.6 0.433 [0.216; 0.680]
Total (95% CI), fixed 4.5 . 0.520 [0.360; 0.677]
Total (95% CI), random . 7.5 0.520 [0.025; 0.978]
Heterogeneity: τ^2 = 0; χ^2 = 0.77, df = 1, P = 0.3803; I^2 = 0%
Category = Anti−EGFR/MEK
Corcoran RB, et al 2018 0.6931 0.4201 2.9 4.0 0.667 [0.467; 0.820]
Category = Anti−EGFR/BRAF
Boccaccino A, et al 2022 −1.3481 0.3549 4.0 4.3 0.206 [0.115; 0.342]
Corcoran RB, et al 2018 −0.2007 0.6356 1.2 3.1 0.450 [0.191; 0.740]
Gallois C, et al 2024 −1.2824 0.2593 7.5 4.6 0.217 [0.143; 0.316]
Hyman DM, et al 2015 1.0498 0.6211 1.3 3.2 0.741 [0.458; 0.906]
Stintzing S, et al 2024 −0.4940 0.2991 5.6 4.5 0.379 [0.253; 0.523]
Tabernero J, et al 2016 0.3228 0.4052 3.1 4.1 0.580 [0.384; 0.753]
Tabernero J, et al 2021 0.2559 0.1923 13.7 4.8 0.564 [0.470; 0.653]
Wang X, et al 2024 −1.1192 0.4072 3.0 4.0 0.246 [0.128; 0.420]
Total (95% CI), fixed 39.5 . 0.402 [0.350; 0.457]
Total (95% CI), random . 32.6 0.399 [0.247; 0.574]
Heterogeneity: τ^2 = 0.5435; χ^2 = 42.3, df = 7, P < 0.0001; I^2 = 83.5%
Category = Anti−EGFR/BRAF/MEK
Boccaccino A, et al 2022 −0.6931 0.5000 2.0 3.6 0.333 [0.158; 0.571]
Corcoran RB, et al 2018 0.8630 0.3245 4.8 4.4 0.703 [0.556; 0.817]
Van Cutsem E, et al 2019 0.8473 0.5634 1.6 3.4 0.700 [0.436; 0.876]
Gallois C, et al 2024 −1.7918 0.8819 0.6 2.3 0.143 [0.029; 0.484]
Kotani D, et al 2024 −0.9249 0.3485 4.2 4.3 0.284 [0.167; 0.440]
Tabernero J, et al 2021 0.6269 0.1983 12.8 4.8 0.652 [0.559; 0.734]
Total (95% CI), fixed 26.0 . 0.568 [0.500; 0.633]
Total (95% CI), random . 22.8 0.486 [0.240; 0.739]
Heterogeneity: τ^2 = 0.7918; χ^2 = 28.56, df = 5, P < 0.0001; I^2 = 82.5%
Category = Anti−EGFR/RAF/PI3K
Tabernero J, et al 2016 1.3157 0.4802 2.2 3.7 0.788 [0.593; 0.905]
Category = Anti−EGFR/BRAF/ERK
Parikh AR, et al 2024 −1.5686 0.6952 1.0 2.9 0.172 [0.051; 0.449]
Category = Anti−BRAF/EGFR/PD−1
Morris VK, et al 2022 −1.7047 0.7687 0.9 2.6 0.154 [0.039; 0.451]
Qiu M, et al 2023 0.0000 0.8165 0.8 2.5 0.500 [0.168; 0.832]
Total (95% CI), fixed 1.6 . 0.288 [0.119; 0.548]
Total (95% CI), random . 5.1 0.294 [0.000; 1.000]
Heterogeneity: τ^2 = 0.8243; χ^2 = 2.31, df = 1, P = 0.1285; I^2 = 56.7%
Category = Anti−EGFR/BRAF/WNT
Tabernero J, et al 2023 1.3863 0.7906 0.8 2.5 0.800 [0.459; 0.950]
Category = Chemo−anti−EGFR/BRAF
Kopetz S, et al 2021 1.5841 0.5489 1.7 3.4 0.830 [0.624; 0.935]
Total (95% CI), fixed 100.0 . 0.501 [0.466; 0.535]
Total (95% CI), random . 100.0 0.483 [0.392; 0.575]
Heterogeneity: τ^2=0.6108; χ^2=122.82, df=26, P < 0.0001 I^2=78.8%
0
0.2
0.4
0.6
0.8
1
Grade 3 or higher AEs rate (95% CI)
in second- or later-line setting

Figure S8. Pooled efficacy estimates (overall survival, OS; progression-free survival, PFS; objective response rate, ORR; disease control rate, DCR; grade 3 or higher AEs) of the second- or later-line regimen in head-to-head comparisons in pairwise meta-analysis.

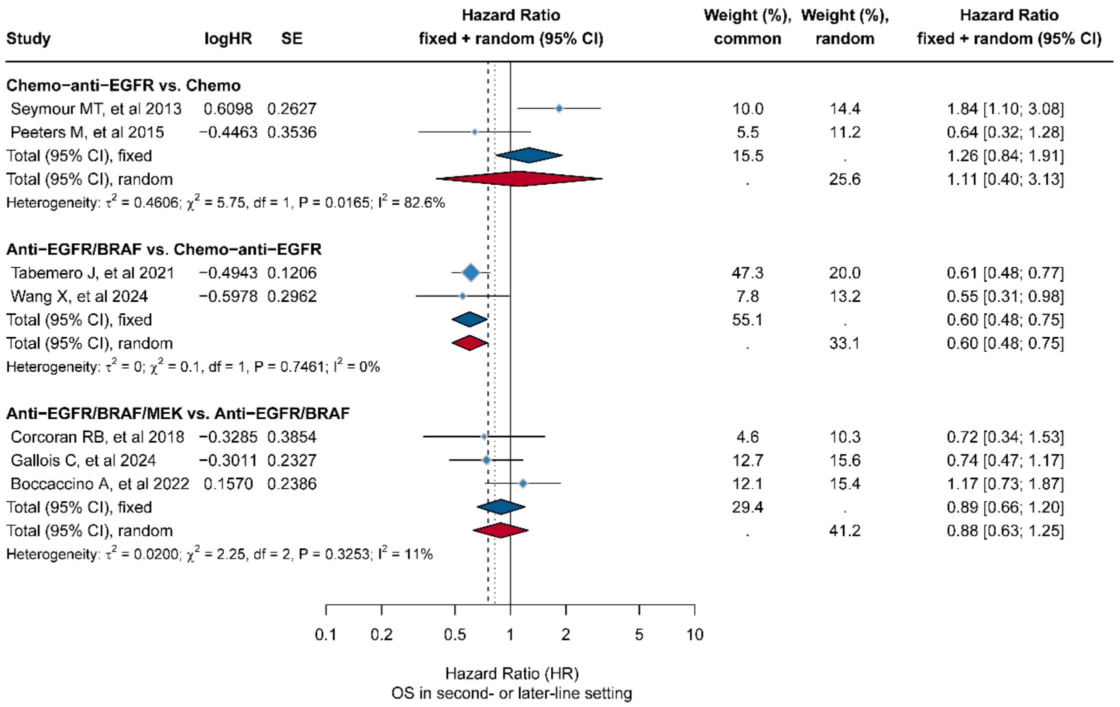

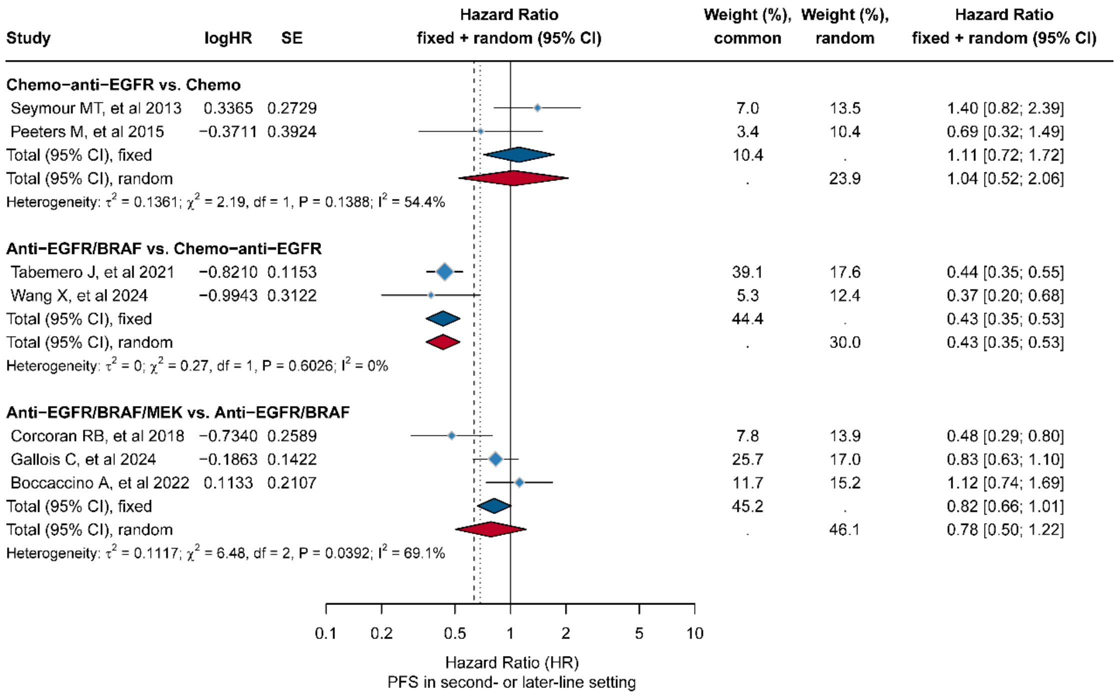

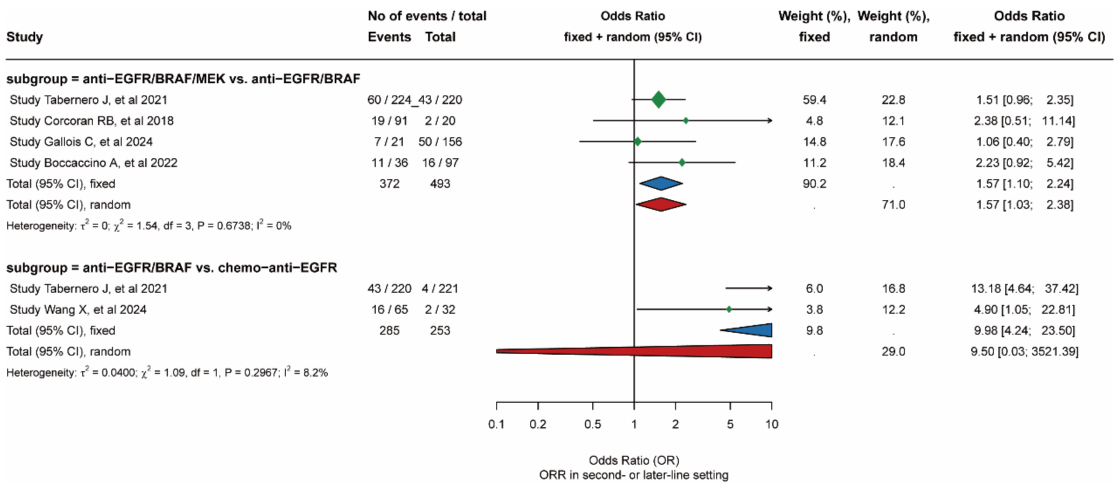
Study
No of events / total
Events Total
Odds Ratio
fixed + random (95% CI)
Weight (%), fixed
Weight (%), random
Odds Ratio
fixed + random (95% CI)
subgroup = anti−EGFR/BRAF/MEK vs. anti−EGFR/BRAF
Study Tabernero J, et al 2021 60 / 224 43 / 220 59.4 22.8 1.51 [0.96; 2.35]
Study Corcoran RB, et al 2018 19 / 91 2 / 20 4.8 12.1 2.38 [0.51; 11.14]
Study Gallois C, et al 2024 7 / 21 50 / 156 14.8 17.6 1.06 [0.40; 2.79]
Study Boccaccino A, et al 2022 11 / 36 16 / 97 11.2 18.4 2.23 [0.92; 5.42]
Total (95% CI), fixed 372 493 90.2 . 1.57 [1.10; 2.24]
Total (95% CI), random . 71.0 1.57 [1.03; 2.38]
Heterogeneity: τ^2 = 0; χ^2 = 1.54, df = 3, P = 0.6738; I^2 = 0%
subgroup = anti−EGFR/BRAF vs. chemo−anti−EGFR
Study Tabernero J, et al 2021 43 / 220 4 / 221 6.0 16.8 13.18 [4.64; 37.42]
Study Wang X, et al 2024 16 / 65 2 / 32 3.8 12.2 4.90 [1.05; 22.81]
Total (95% CI), fixed 285 253 9.8 . 9.98 [4.24; 23.50]
Total (95% CI), random . 29.0 9.50 [0.03; 3521.39]
Heterogeneity: τ^2 = 0.0400; χ^2 = 1.09, df = 1, P = 0.2967; I^2 = 8.2%
0.1 0.2 0.5 1 2 5 10
Odds Ratio (OR)
ORR in second- or later-line setting

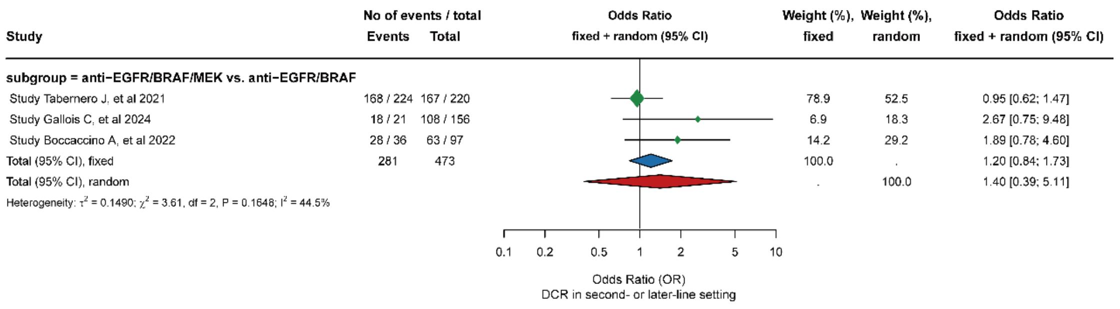
Study
No of events / total
Events Total
Odds Ratio
fixed + random (95% CI)
Weight (%), fixed
Weight (%), random
Odds Ratio
fixed + random (95% CI)
subgroup = anti−EGFR/BRAF/MEK vs. anti−EGFR/BRAF
Study Tabernero J, et al 2021 168 / 224 167 / 220 78.9 52.5 0.95 [0.62; 1.47]
Study Gallois C, et al 2024 18 / 21 108 / 156 6.9 18.3 2.67 [0.75; 9.48]
Study Boccaccino A, et al 2022 28 / 36 63 / 97 14.2 29.2 1.89 [0.78; 4.60]
Total (95% CI), fixed 281 473 100.0 . 1.20 [0.84; 1.73]
Total (95% CI), random . 100.0 1.40 [0.39; 5.11]
Heterogeneity: τ^2 = 0.1490; χ^2 = 3.61, df = 2, P = 0.1648; I^2 = 44.5%
0.1 0.2 0.5 1 2 5 10
Odds Ratio (OR)
DCR in second- or later-line setting

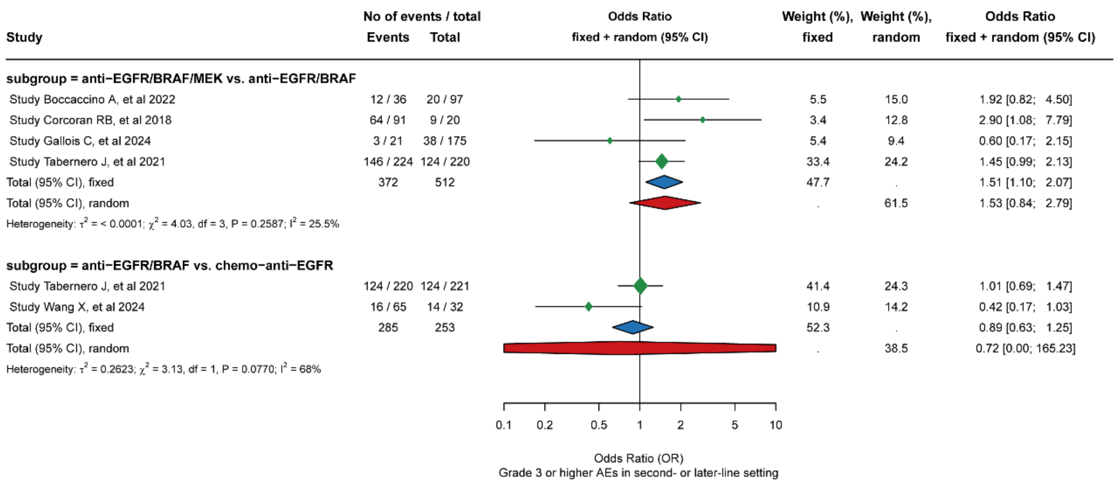
Study
No of events / total
Events Total
Odds Ratio
fixed + random (95% CI)
Weight (%), fixed
Weight (%), random
Odds Ratio
fixed + random (95% CI)
subgroup = anti−EGFR/BRAF/MEK vs. anti−EGFR/BRAF
Study Boccaccino A, et al 2022 12 / 36 20 / 97 5.5 15.0 1.92 [0.82; 4.50]
Study Corcoran RB, et al 2018 64 / 91 9 / 20 3.4 12.8 2.90 [1.08; 7.79]
Study Gallois C, et al 2024 3 / 21 38 / 175 5.4 9.4 0.60 [0.17; 2.15]
Study Tabernero J, et al 2021 146 / 224 124 / 220 33.4 24.2 1.45 [0.99; 2.13]
Total (95% CI), fixed 372 512 47.7 . 1.51 [1.10; 2.07]
Total (95% CI), random . 61.5 1.53 [0.84; 2.79]
Heterogeneity: τ^2 = < 0.0001; χ^2 = 4.03, df = 3, P = 0.2587; I^2 = 25.5%
subgroup = anti−EGFR/BRAF vs. chemo−anti−EGFR
Study Tabernero J, et al 2021 124 / 220 124 / 221 41.4 24.3 1.01 [0.69; 1.47]
Study Wang X, et al 2024 16 / 65 14 / 32 10.9 14.2 0.42 [0.17; 1.03]
Total (95% CI), fixed 285 253 52.3 . 0.89 [0.63; 1.25]
Total (95% CI), random . 38.5 0.72 [0.00; 165.23]
Heterogeneity: τ^2 = 0.2623; χ^2 = 3.13, df = 1, P = 0.0770; I^2 = 68%
0.1 0.2 0.5 1 2 5 10
Odds Ratio (OR)
Grade 3 or higher AEs in second- or later-line setting

Figure S9: Pooled efficacy (objective response rare, ORR; disease control rate, DCR) and safety (grade 3 or higher AEs) estimates of multiple comparisons in second- or later--line setting based on the network meta-analysis.

**ORR-Comparison**	Chemo	Chemo-anti-EGFR	Chemo-anti-EGFR/BRAF	Anti-EGFR/BRAF	Anti-EGFR/BRAF/PI3K	Anti-EGFR/BRAF/MEK
Chemo	Reference	1.86 (0.22, 22.4)	9.10 (0.49, 238)	**19.8 (1.65, 319)**	**26.1 (1.40, 614)**	**33.1 (2.70, 538)**
Chemo-anti-EGFR	0.54 (0.04, 4.49)	Reference	4.54 (0.65, 48.6)	**10.6 (3.06, 34.6)**	**14.0 (1.87, 93.4)**	**17.7 (4.87, 63.2)**
Chemo-anti-EGFR/BRAF	0.11 (0.01, 2.03)	0.22 (0.02, 1.53)	Reference	2.33 (0.16, 21.6)	3.02 (0.14, 44.5)	3.86 (0.26, 38.4)
Anti-EGFR/BRAF	**0.05 (0.01, 0.61)**	**0.09 (0.03, 0.33)**	0.43 (0.05, 6.24)	Reference	1.31 (0.29, 6.08)	1.65 (0.81, 3.71)
Anti-EGFR/BRAF/PI3K	**0.04 (0.01, 0.71)**	**0.07 (0.01, 0.53)**	0.33 (0.02, 7.09)	0.76 (0.16, 3.48)	Reference	1.25 (0.24, 7.13)
Anti-EGFR/BRAF/MEK	**0.03 (0.01, 0.37)**	**0.06 (0.02, 0.21)**	0.26 (0.03, 3.82)	0.60 (0.27, 1.23)	0.80 (0.14, 4.15)	Reference

**DCR-Comparison**	Chemo-anti-EGFR	Chemo-anti-EGFR/BRAF	Anti-EGFR/BRAF	Anti-EGFR/BRAF/MEK
Chemo-anti-EGFR	Reference	6.50 (0.78, 53.3)	5.67 (0.81, 32.0)	**8.19 (1.42, 55.9)**
Chemo-anti-EGFR/BRAF	0.15 (0.02, 1.29)	Reference	0.86 (0.05, 13.0)	1.26 (0.08, 22.2)
Anti-EGFR/BRAF	0.18 (0.03, 1.23)	1.16 (0.08, 20.9)	Reference	1.47 (0.51, 5.77)
Anti-EGFR/BRAF/MEK	0.12 (0.02, 0.70)	0.79 (0.05, 12.0)	0.68 (0.17, 1.97)	Reference

**AE-Comparison**	Chemo-anti-EGFR	Chemo-anti-EGFR/BRAF	Anti-EGFR/MEK	Anti-EGFR/BRAF	Anti-EGFR/BRAF/PI3K	Anti-EGFR/BRAF/MEK
Chemo-anti-EGFR	Reference	3.86 (0.82, 19.2)	1.27 (0.25, 6.19)	0.78 (0.28, 1.84)	2.13 (0.33, 11.6)	1.26 (0.40, 3.30)
Chemo-anti-EGFR/BRAF	0.26 (0.05, 1.22)	Reference	0.33 (0.03, 2.95)	0.20 (0.03, 1.13)	0.55 (0.05, 5.50)	0.32 (0.04, 1.92)
Anti-BRAF/MEK	0.79 (0.16, 4.08)	3.05 (0.34, 29.6)	Reference	0.61 (0.14, 2.32)	1.71 (0.2, 12.4)	0.99 (0.25, 3.61)
Anti-EGFR/BRAF	1.28 (0.54, 3.63)	4.97 (0.89, 33.9)	1.63 (0.43, 6.97)	Reference	2.76 (0.61, 12.6)	1.61 (0.77, 3.39)
Anti-EGFR/BRAF/PI3K	0.47 (0.09, 3.02)	1.81 (0.18, 21.2)	0.59 (0.08, 4.92)	0.36 (0.08, 1.64)	Reference	0.58 (0.11, 3.13)
Anti-EGFR/BRAF/MEK	0.79 (0.30, 2.51)	3.11 (0.52, 22.3)	1.01 (0.28, 4.08)	0.62 (0.29, 1.30)	1.72 (0.32, 9.33)	Reference

Figure S10: Pooled efficacy and safety estimates (objective response rate, ORR; disease control rate, DCR; grade-3 or higher AE) in single-arm meta-analysis after excluding conferences reports.

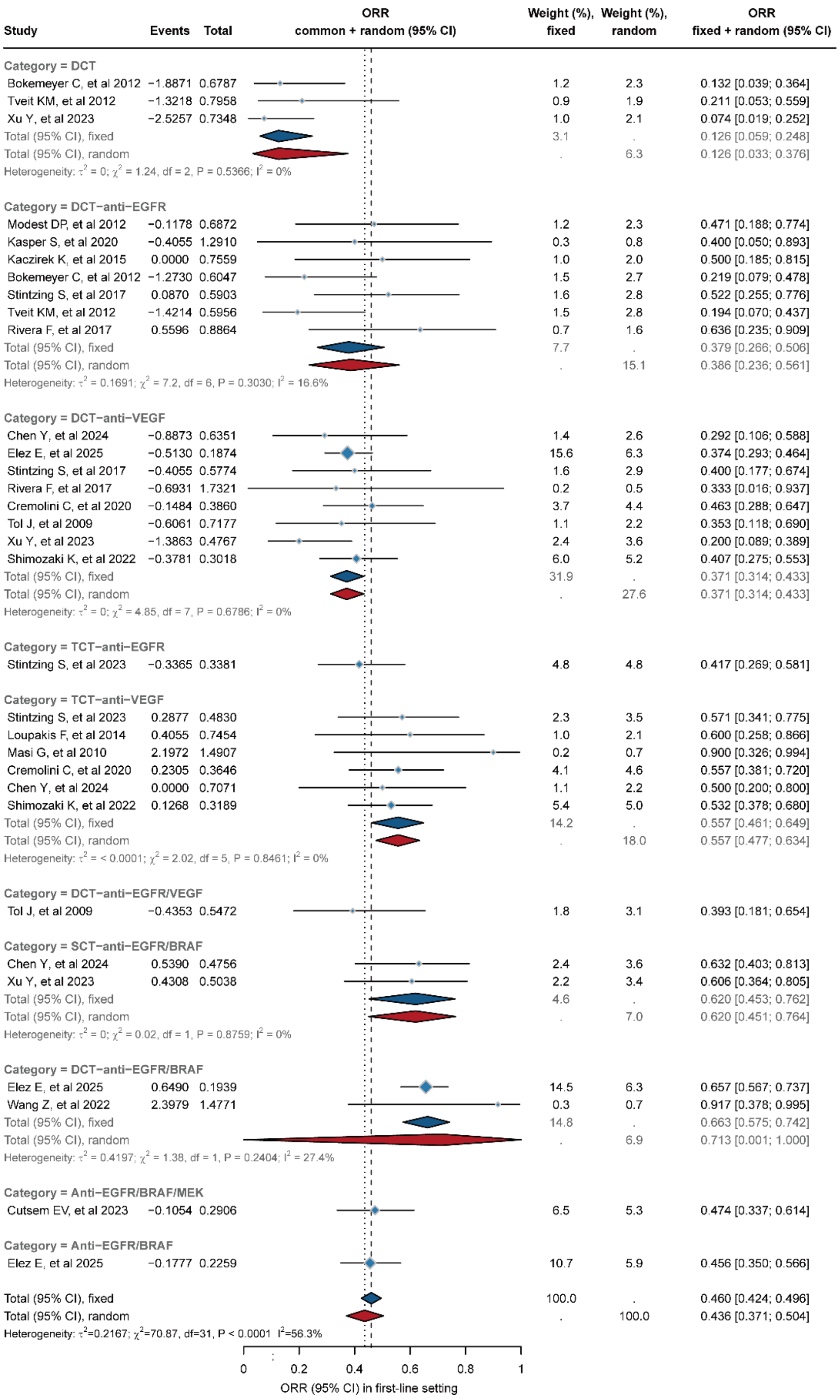

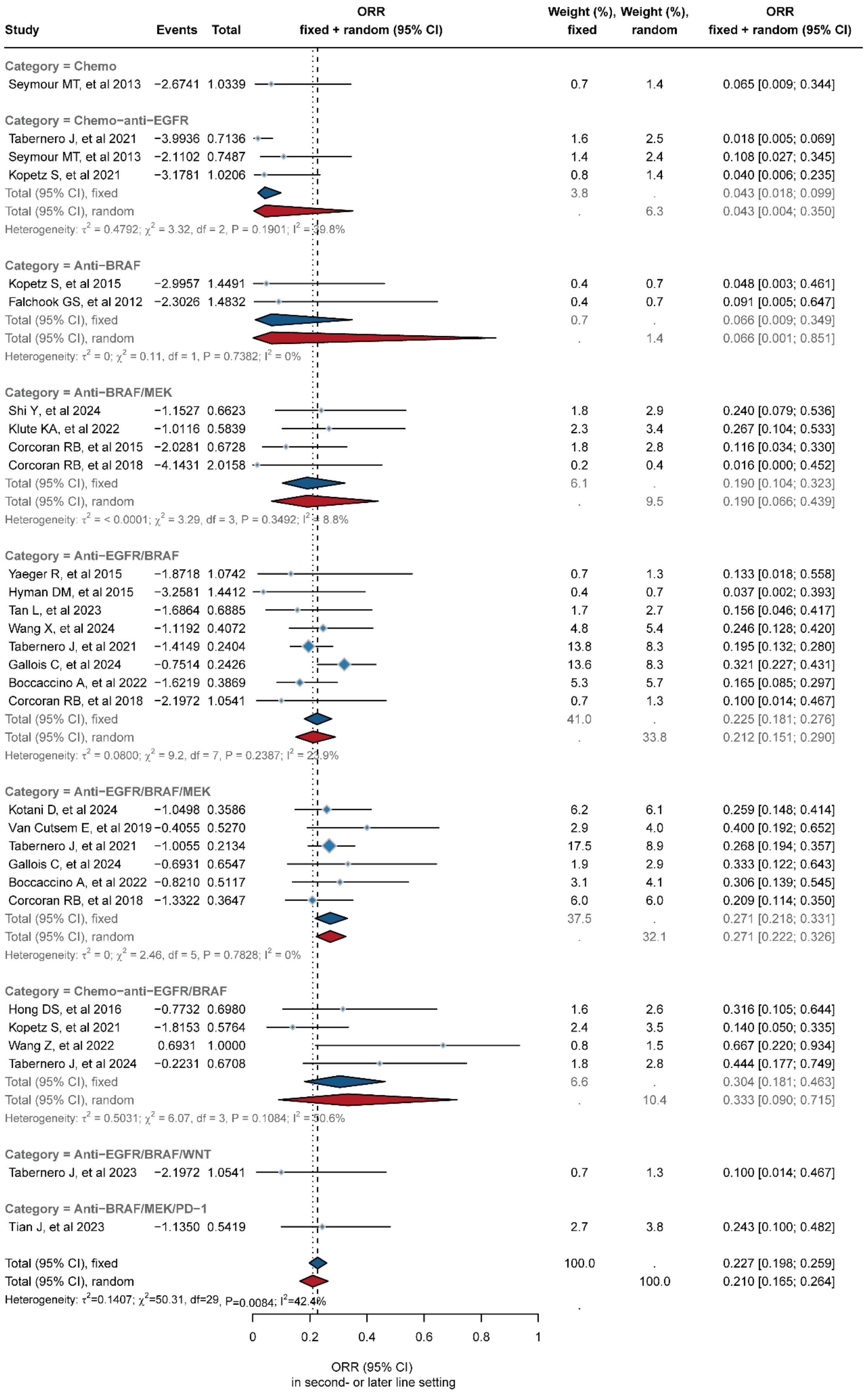
Study
Events
Total
ORR fixed + random (95% CI)
Weight (%), fixed
Weight (%), random
ORR fixed + random (95% CI)
Category = Chemo
Seymour MT, et al 2013 −2.6741 1.0339 0.7 1.4 0.065 [0.009; 0.344]
Category = Chemo−anti−EGFR
Tabernero J, et al 2021 −3.9936 0.7136 1.6 2.5 0.018 [0.005; 0.069]
Seymour MT, et al 2013 −2.1102 0.7487 1.4 2.4 0.108 [0.027; 0.345]
Kopetz S, et al 2021 −3.1781 1.0206 0.8 1.4 0.040 [0.006; 0.235]
Total (95% CI), fixed 3.8 . 0.043 [0.018; 0.099]
Total (95% CI), random . 6.3 0.043 [0.004; 0.350]
Heterogeneity: τ^2 = 0.4792; χ^2 = 3.32, df = 2, P = 0.1901; I^2 = 39.8%
Category = Anti−BRAF
Kopetz S, et al 2015 −2.9957 1.4491 0.4 0.7 0.048 [0.003; 0.461]
Falchook GS, et al 2012 −2.3026 1.4832 0.4 0.7 0.091 [0.005; 0.647]
Total (95% CI), fixed 0.7 . 0.066 [0.009; 0.349]
Total (95% CI), random . 1.4 0.066 [0.001; 0.851]
Heterogeneity: τ^2 = 0; χ^2 = 0.11, df = 1, P = 0.7382; I^2 = 0%
Category = Anti−BRAF/MEK
Shi Y, et al 2024 −1.1527 0.6623 1.8 2.9 0.240 [0.079; 0.536]
Klute KA, et al 2022 −1.0116 0.5839 2.3 3.4 0.267 [0.104; 0.533]
Corcoran RB, et al 2015 −2.0281 0.6728 1.8 2.8 0.116 [0.034; 0.330]
Corcoran RB, et al 2018 −4.1431 2.0158 0.2 0.4 0.016 [0.000; 0.452]
Total (95% CI), fixed 6.1 . 0.190 [0.104; 0.323]
Total (95% CI), random . 9.5 0.190 [0.066; 0.439]
Heterogeneity: τ^2 = < 0.0001; χ^2 = 3.29, df = 3, P = 0.3492; I^2 = 8.8%
Category = Anti−EGFR/BRAF
Yaeger R, et al 2015 −1.8718 1.0742 0.7 1.3 0.133 [0.018; 0.558]
Hyman DM, et al 2015 −3.2581 1.4412 0.4 0.7 0.037 [0.002; 0.393]
Tan L, et al 2023 −1.6864 0.6885 1.7 2.7 0.156 [0.046; 0.417]
Wang X, et al 2024 −1.1192 0.4072 4.8 5.4 0.246 [0.128; 0.420]
Tabernero J, et al 2021 −1.4149 0.2404 13.8 8.3 0.195 [0.132; 0.280]
Gallois C, et al 2024 −0.7514 0.2426 13.6 8.3 0.321 [0.227; 0.431]
Boccaccino A, et al 2022 −1.6219 0.3869 5.3 5.7 0.165 [0.085; 0.297]
Corcoran RB, et al 2018 −2.1972 1.0541 0.7 1.3 0.100 [0.014; 0.467]
Total (95% CI), fixed 41.0 . 0.225 [0.181; 0.276]
Total (95% CI), random . 33.8 0.212 [0.151; 0.290]
Heterogeneity: τ^2 = 0.0800; χ^2 = 9.2, df = 7, P = 0.2387; I^2 = 23.9%
Category = Anti−EGFR/BRAF/MEK
Kotani D, et al 2024 −1.0498 0.3586 6.2 6.1 0.259 [0.148; 0.414]
Van Cutsem E, et al 2019 −0.4055 0.5270 2.9 4.0 0.400 [0.192; 0.652]
Tabernero J, et al 2021 −1.0055 0.2134 17.5 8.9 0.268 [0.194; 0.357]
Gallois C, et al 2024 −0.6931 0.6547 1.9 2.9 0.333 [0.122; 0.643]
Boccaccino A, et al 2022 −0.8210 0.5117 3.1 4.1 0.306 [0.139; 0.545]
Corcoran RB, et al 2018 −1.3322 0.3647 6.0 6.0 0.209 [0.114; 0.350]
Total (95% CI), fixed 37.5 . 0.271 [0.218; 0.331]
Total (95% CI), random . 32.1 0.271 [0.222; 0.326]
Heterogeneity: τ^2 = 0; χ^2 = 2.46, df = 5, P = 0.7828; I^2 = 0%
Category = Chemo−anti−EGFR/BRAF
Hong DS, et al 2016 −0.7732 0.6980 1.6 2.6 0.316 [0.105; 0.644]
Kopetz S, et al 2021 −1.8153 0.5764 2.4 3.5 0.140 [0.050; 0.335]
Wang Z, et al 2022 0.6931 1.0000 0.8 1.5 0.667 [0.220; 0.934]
Tabernero J, et al 2024 −0.2231 0.6708 1.8 2.8 0.444 [0.177; 0.749]
Total (95% CI), fixed 6.6 . 0.304 [0.181; 0.463]
Total (95% CI), random . 10.4 0.333 [0.090; 0.715]
Heterogeneity: τ^2 = 0.5031; χ^2 = 6.07, df = 3, P = 0.1084; I^2 = 50.6%
Category = Anti−EGFR/BRAF/WNT
Tabernero J, et al 2023 −2.1972 1.0541 0.7 1.3 0.100 [0.014; 0.467]
Category = Anti−BRAF/MEK/PD−1
Tian J, et al 2023 −1.1350 0.5419 2.7 3.8 0.243 [0.100; 0.482]
Total (95% CI), fixed 100.0 . 0.227 [0.198; 0.259]
Total (95% CI), random . 100.0 0.210 [0.165; 0.264]
Heterogeneity: τ^2=0.1407; χ^2=50.31, df=29, P=0.0084; I^2=42.4%
0
0.2
0.4
0.6
0.8
1
ORR (95% CI)
in second- or later line setting

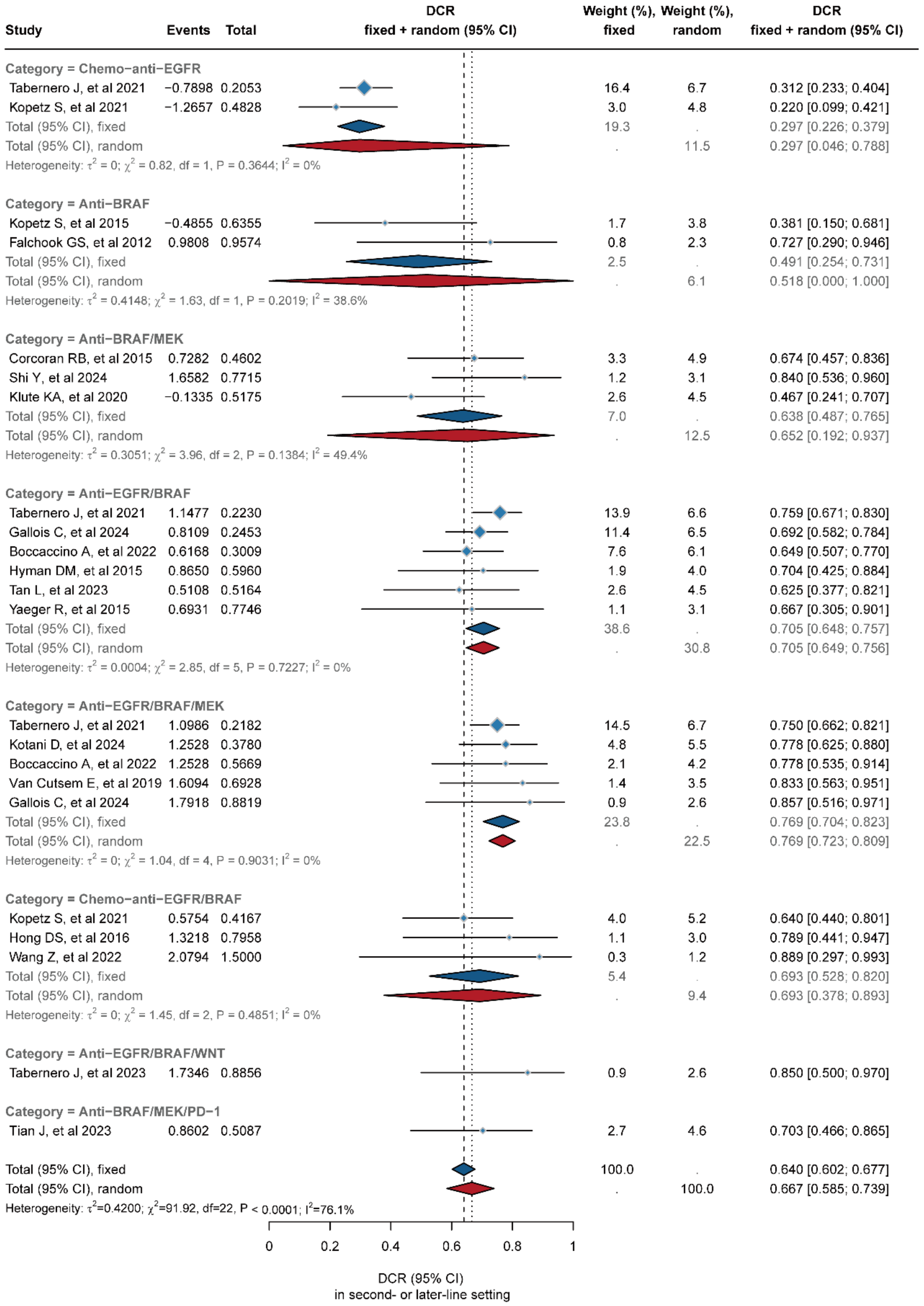
Study
Events
Total
DCR fixed + random (95% CI)
Weight (%), fixed
Weight (%), random
DCR fixed + random (95% CI)
Category = Chemo−anti−EGFR
Tabernero J, et al 2021 −0.7898 0.2053 16.4 6.7 0.312 [0.233; 0.404]
Kopetz S, et al 2021 −1.2657 0.4828 3.0 4.8 0.220 [0.099; 0.421]
Total (95% CI), fixed 19.3 . 0.297 [0.226; 0.379]
Total (95% CI), random . 11.5 0.297 [0.046; 0.788]
Heterogeneity: τ^2 = 0; χ^2 = 0.82, df = 1, P = 0.3644; I^2 = 0%
Category = Anti−BRAF
Kopetz S, et al 2015 −0.4855 0.6355 1.7 3.8 0.381 [0.150; 0.681]
Falchook GS, et al 2012 0.9808 0.9574 0.8 2.3 0.727 [0.290; 0.946]
Total (95% CI), fixed 2.5 . 0.491 [0.254; 0.731]
Total (95% CI), random . 6.1 0.518 [0.000; 1.000]
Heterogeneity: τ^2 = 0.4148; χ^2 = 1.63, df = 1, P = 0.2019; I^2 = 38.6%
Category = Anti−BRAF/MEK
Corcoran RB, et al 2015 0.7282 0.4602 3.3 4.9 0.674 [0.457; 0.836]
Shi Y, et al 2024 1.6582 0.7715 1.2 3.1 0.840 [0.536; 0.960]
Klute KA, et al 2020 −0.1335 0.5175 2.6 4.5 0.467 [0.241; 0.707]
Total (95% CI), fixed 7.0 . 0.638 [0.487; 0.765]
Total (95% CI), random . 12.5 0.652 [0.192; 0.937]
Heterogeneity: τ^2 = 0.3051; χ^2 = 3.96, df = 2, P = 0.1384; I^2 = 49.4%
Category = Anti−EGFR/BRAF
Tabernero J, et al 2021 1.1477 0.2230 13.9 6.6 0.759 [0.671; 0.830]
Gallois C, et al 2024 0.8109 0.2453 11.4 6.5 0.692 [0.582; 0.784]
Boccaccino A, et al 2022 0.6168 0.3009 7.6 6.1 0.649 [0.507; 0.770]
Hyman DM, et al 2015 0.8650 0.5960 1.9 4.0 0.704 [0.425; 0.884]
Tan L, et al 2023 0.5108 0.5164 2.6 4.5 0.625 [0.377; 0.821]
Yaeger R, et al 2015 0.6931 0.7746 1.1 3.1 0.667 [0.305; 0.901]
Total (95% CI), fixed 38.6 . 0.705 [0.648; 0.757]
Total (95% CI), random . 30.8 0.705 [0.649; 0.756]
Heterogeneity: τ^2 = 0.0004; χ^2 = 2.85, df = 5, P = 0.7227; I^2 = 0%
Category = Anti−EGFR/BRAF/MEK
Tabernero J, et al 2021 1.0986 0.2182 14.5 6.7 0.750 [0.662; 0.821]
Kotani D, et al 2024 1.2528 0.3780 4.8 5.5 0.778 [0.625; 0.880]
Boccaccino A, et al 2022 1.2528 0.5669 2.1 4.2 0.778 [0.535; 0.914]
Van Cutsem E, et al 2019 1.6094 0.6928 1.4 3.5 0.833 [0.563; 0.951]
Gallois C, et al 2024 1.7918 0.8819 0.9 2.6 0.857 [0.516; 0.971]
Total (95% CI), fixed 23.8 . 0.769 [0.704; 0.823]
Total (95% CI), random . 22.5 0.769 [0.723; 0.809]
Heterogeneity: τ^2 = 0; χ^2 = 1.04, df = 4, P = 0.9031; I^2 = 0%
Category = Chemo−anti−EGFR/BRAF
Kopetz S, et al 2021 0.5754 0.4167 4.0 5.2 0.640 [0.440; 0.801]
Hong DS, et al 2016 1.3218 0.7958 1.1 3.0 0.789 [0.441; 0.947]
Wang Z, et al 2022 2.0794 1.5000 0.3 1.2 0.889 [0.297; 0.993]
Total (95% CI), fixed 5.4 . 0.693 [0.528; 0.820]
Total (95% CI), random . 9.4 0.693 [0.378; 0.893]
Heterogeneity: τ^2 = 0; χ^2 = 1.45, df = 2, P = 0.4851; I^2 = 0%
Category = Anti−EGFR/BRAF/WNT
Tabernero J, et al 2023 1.7346 0.8856 0.9 2.6 0.850 [0.500; 0.970]
Category = Anti−BRAF/MEK/PD−1
Tian J, et al 2023 0.8602 0.5087 2.7 4.6 0.703 [0.466; 0.865]
Total (95% CI), fixed 100.0 . 0.640 [0.602; 0.677]
Total (95% CI), random . 100.0 0.667 [0.585; 0.739]
Heterogeneity: τ^2=0.4200; χ^2=91.92, df=22, P < 0.0001; I^2=76.1%
0
0.2
0.4
0.6
0.8
1
DCR (95% CI)
in second- or later-line setting

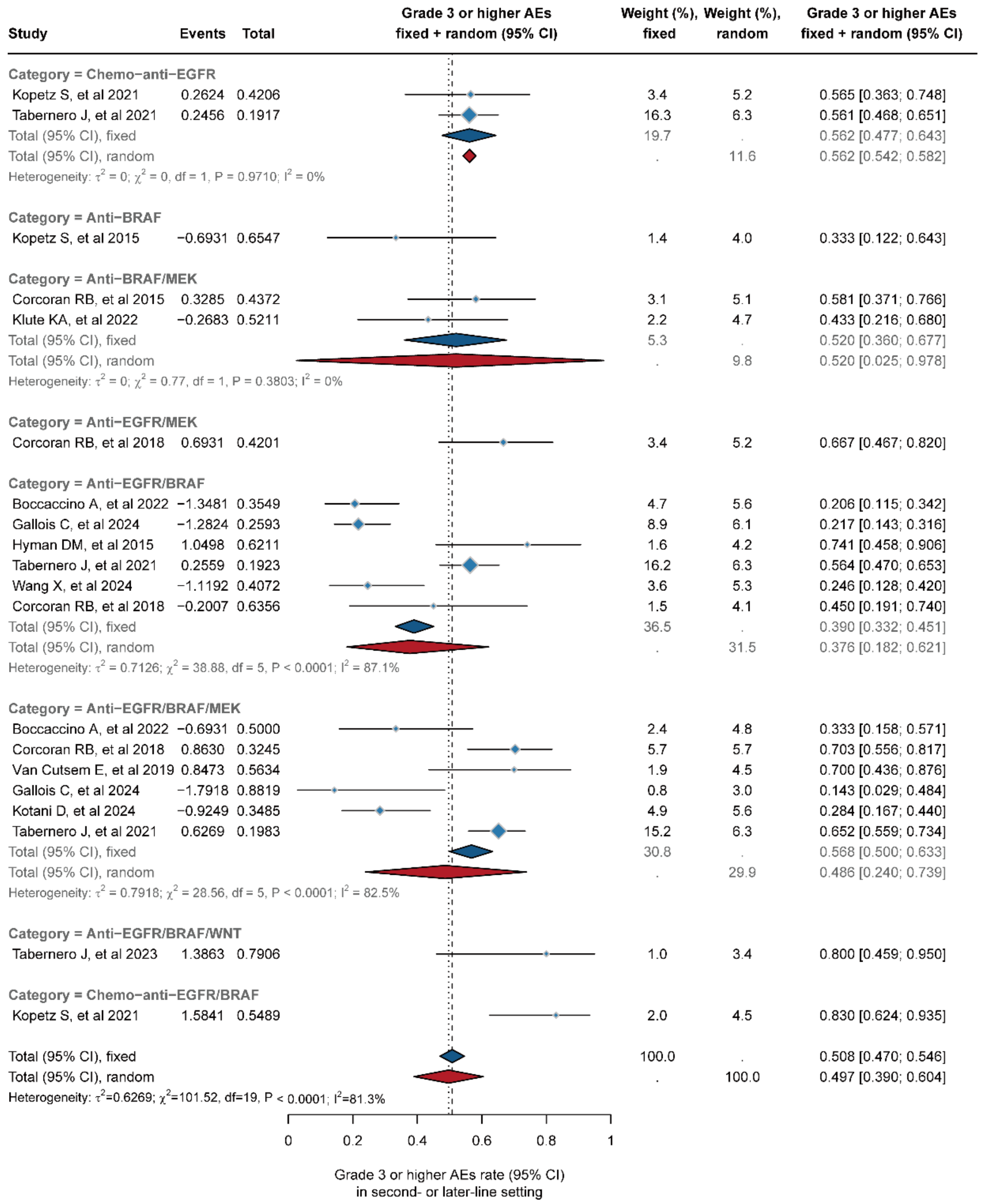
Study Events Total
Grade 3 or higher AEs fixed + random (95% CI)
Weight (%), fixed
Weight (%), random
Grade 3 or higher AEs fixed + random (95% CI)
Category = Chemo−anti−EGFR
Kopetz S, et al 2021 0.2624 0.4206 3.4 5.2 0.565 [0.363; 0.748]
Tabernero J, et al 2021 0.2456 0.1917 16.3 6.3 0.561 [0.468; 0.651]
Total (95% CI), fixed 19.7 . 0.562 [0.477; 0.643]
Total (95% CI), random . 11.6 0.562 [0.542; 0.582]
Heterogeneity: τ² = 0; χ² = 0, df = 1, P = 0.9710; I² = 0%
Category = Anti−BRAF
Kopetz S, et al 2015 −0.6931 0.6547 1.4 4.0 0.333 [0.122; 0.643]
Category = Anti−BRAF/MEK
Corcoran RB, et al 2015 0.3285 0.4372 3.1 5.1 0.581 [0.371; 0.766]
Klute KA, et al 2022 −0.2683 0.5211 2.2 4.7 0.433 [0.216; 0.680]
Total (95% CI), fixed 5.3 . 0.520 [0.360; 0.677]
Total (95% CI), random . 9.8 0.520 [0.025; 0.978]
Heterogeneity: τ² = 0; χ² = 0.77, df = 1, P = 0.3803; I² = 0%
Category = Anti−EGFR/MEK
Corcoran RB, et al 2018 0.6931 0.4201 3.4 5.2 0.667 [0.467; 0.820]
Category = Anti−EGFR/BRAF
Boccaccino A, et al 2022 −1.3481 0.3549 4.7 5.6 0.206 [0.115; 0.342]
Gallois C, et al 2024 −1.2824 0.2593 8.9 6.1 0.217 [0.143; 0.316]
Hyman DM, et al 2015 1.0498 0.6211 1.6 4.2 0.741 [0.458; 0.906]
Tabernero J, et al 2021 0.2559 0.1923 16.2 6.3 0.564 [0.470; 0.653]
Wang X, et al 2024 −1.1192 0.4072 3.6 5.3 0.246 [0.128; 0.420]
Corcoran RB, et al 2018 −0.2007 0.6356 1.5 4.1 0.450 [0.191; 0.740]
Total (95% CI), fixed 36.5 . 0.390 [0.332; 0.451]
Total (95% CI), random . 31.5 0.376 [0.182; 0.621]
Heterogeneity: τ² = 0.7126; χ² = 38.88, df = 5, P < 0.0001; I² = 87.1%
Category = Anti−EGFR/BRAF/MEK
Boccaccino A, et al 2022 −0.6931 0.5000 2.4 4.8 0.333 [0.158; 0.571]
Corcoran RB, et al 2018 0.8630 0.3245 5.7 5.7 0.703 [0.556; 0.817]
Van Cutsem E, et al 2019 0.8473 0.5634 1.9 4.5 0.700 [0.436; 0.876]
Gallois C, et al 2024 −1.7918 0.8819 0.8 3.0 0.143 [0.029; 0.484]
Kotani D, et al 2024 −0.9249 0.3485 4.9 5.6 0.284 [0.167; 0.440]
Tabernero J, et al 2021 0.6269 0.1983 15.2 6.3 0.652 [0.559; 0.734]
Total (95% CI), fixed 30.8 . 0.568 [0.500; 0.633]
Total (95% CI), random . 29.9 0.486 [0.240; 0.739]
Heterogeneity: τ² = 0.7918; χ² = 28.56, df = 5, P < 0.0001; I² = 82.5%
Category = Anti−EGFR/BRAF/WNT
Tabernero J, et al 2023 1.3863 0.7906 1.0 3.4 0.800 [0.459; 0.950]
Category = Chemo−anti−EGFR/BRAF
Kopetz S, et al 2021 1.5841 0.5489 2.0 4.5 0.830 [0.624; 0.935]
Total (95% CI), fixed 100.0 . 0.508 [0.470; 0.546]
Total (95% CI), random . 100.0 0.497 [0.390; 0.604]
Heterogeneity: τ²=0.6269; χ²=101.52, df=19, P < 0.0001; I²=81.3%
0 0.2 0.4 0.6 0.8 1
Grade 3 or higher AEs rate (95% CI)
in second- or later-line setting

Figure S11. Forest plot of the pooled estimates using direct comparison, indirect comparison, and network meta-analysis via the node-splitting method. (A p-value greater than 0.05 indicates no statistically significant difference).

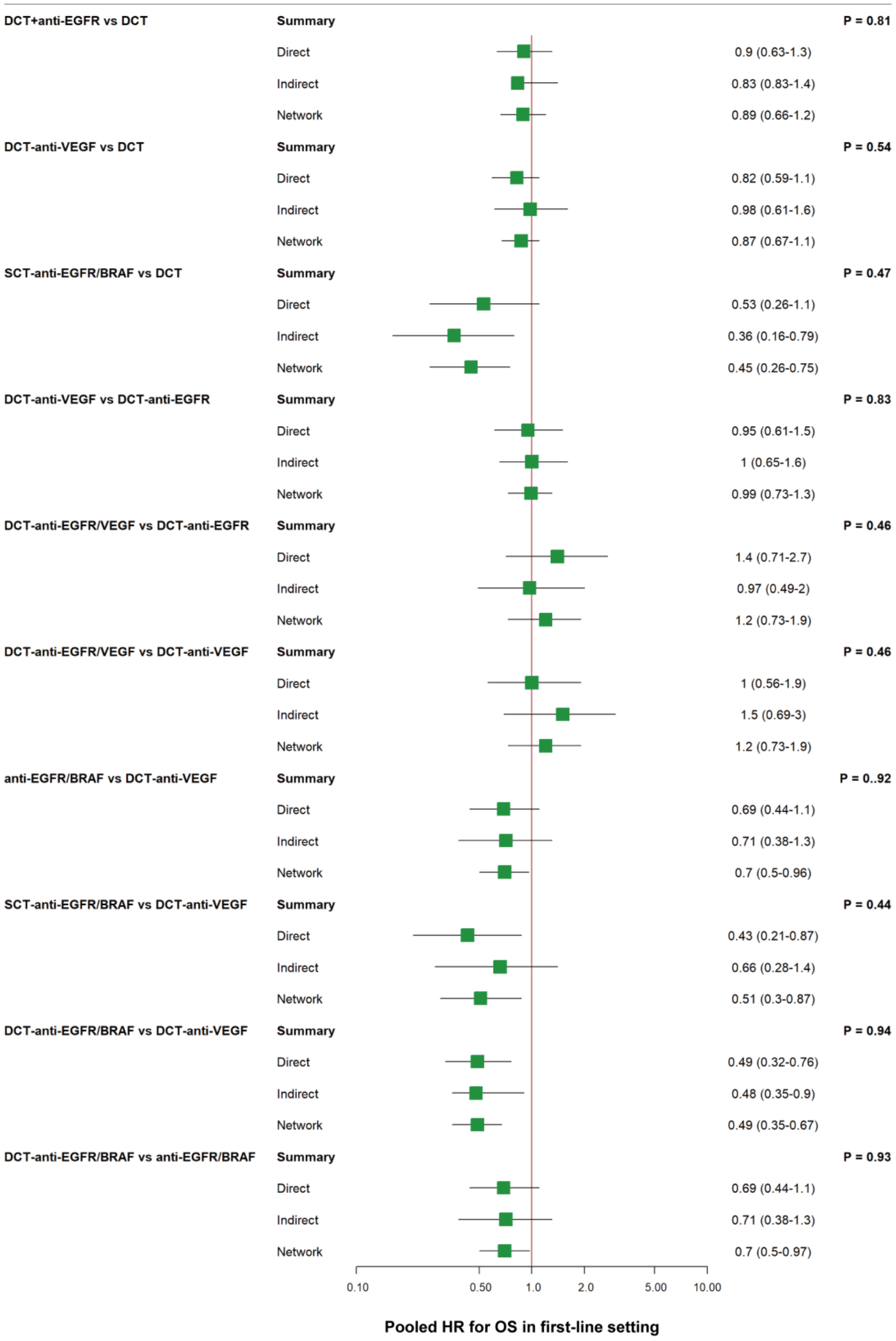

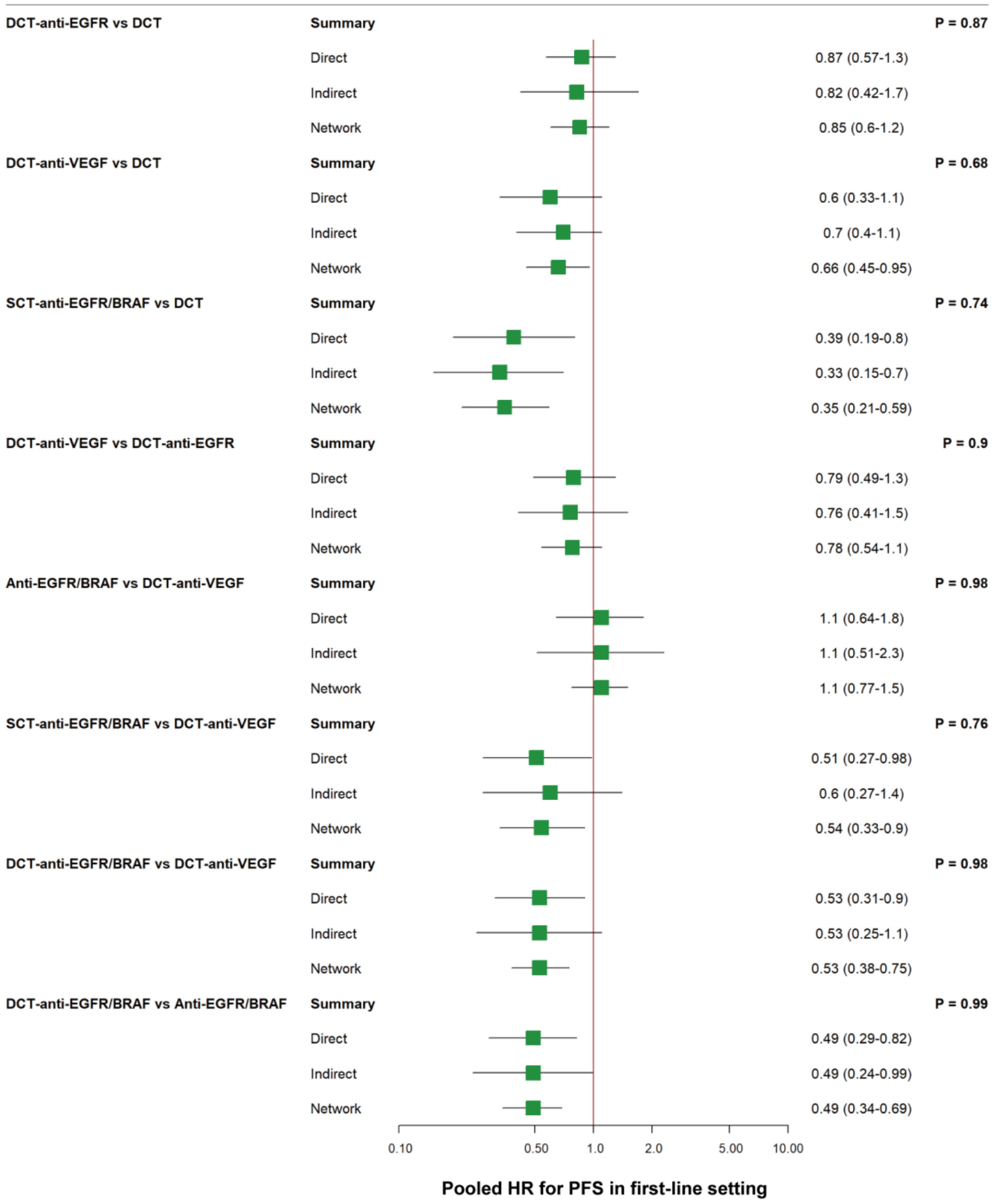
DCT-anti-EGFR vs DCT
Summary
P = 0.87
Direct
0.87 (0.57-1.3)
Indirect
0.82 (0.42-1.7)
Network
0.85 (0.6-1.2)
DCT-anti-VEGF vs DCT
Summary
P = 0.68
Direct
0.6 (0.33-1.1)
Indirect
0.7 (0.4-1.1)
Network
0.66 (0.45-0.95)
SCT-anti-EGFR/BRAF vs DCT
Summary
P = 0.74
Direct
0.39 (0.19-0.8)
Indirect
0.33 (0.15-0.7)
Network
0.35 (0.21-0.59)
DCT-anti-VEGF vs DCT-anti-EGFR
Summary
P = 0.9
Direct
0.79 (0.49-1.3)
Indirect
0.76 (0.41-1.5)
Network
0.78 (0.54-1.1)
Anti-EGFR/BRAF vs DCT-anti-VEGF
Summary
P = 0.98
Direct
1.1 (0.64-1.8)
Indirect
1.1 (0.51-2.3)
Network
1.1 (0.77-1.5)
SCT-anti-EGFR/BRAF vs DCT-anti-VEGF
Summary
P = 0.76
Direct
0.51 (0.27-0.98)
Indirect
0.6 (0.27-1.4)
Network
0.54 (0.33-0.9)
DCT-anti-EGFR/BRAF vs DCT-anti-VEGF
Summary
P = 0.98
Direct
0.53 (0.31-0.9)
Indirect
0.53 (0.25-1.1)
Network
0.53 (0.38-0.75)
DCT-anti-EGFR/BRAF vs Anti-EGFR/BRAF
Summary
P = 0.99
Direct
0.49 (0.29-0.82)
Indirect
0.49 (0.24-0.99)
Network
0.49 (0.34-0.69)
0.10
0.50
1.0
2.0
5.00
10.00
Pooled HR for PFS in first-line setting

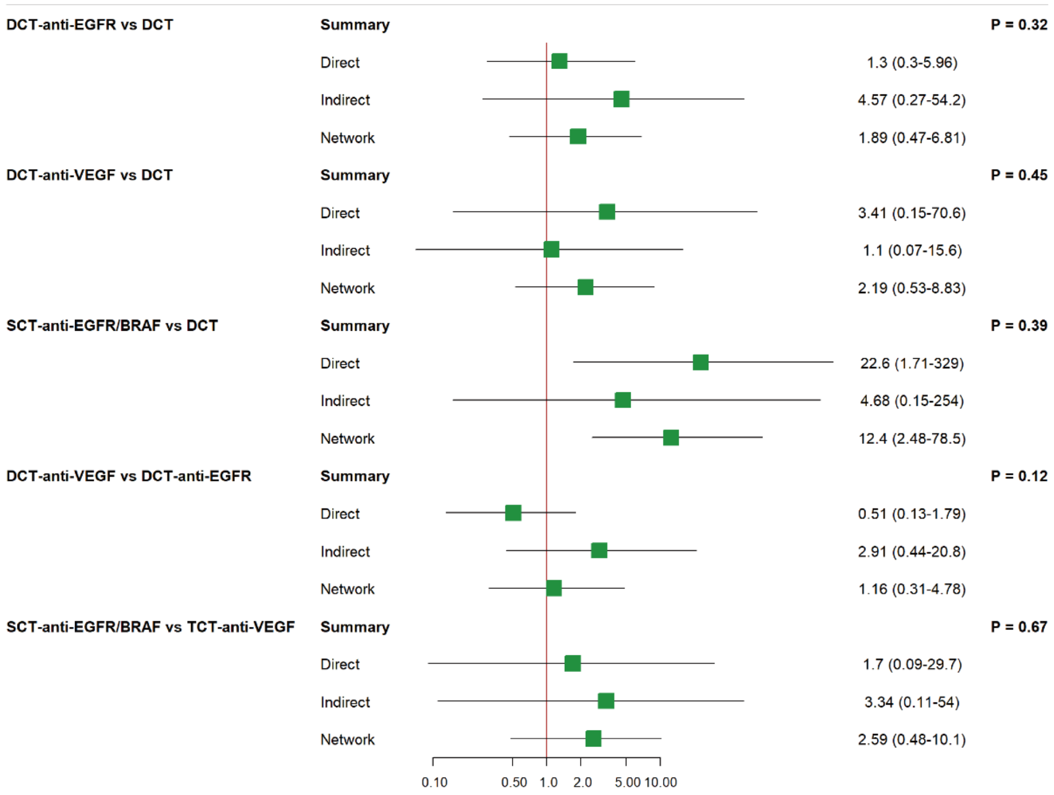

Pooled OR for ORR in first-line setting

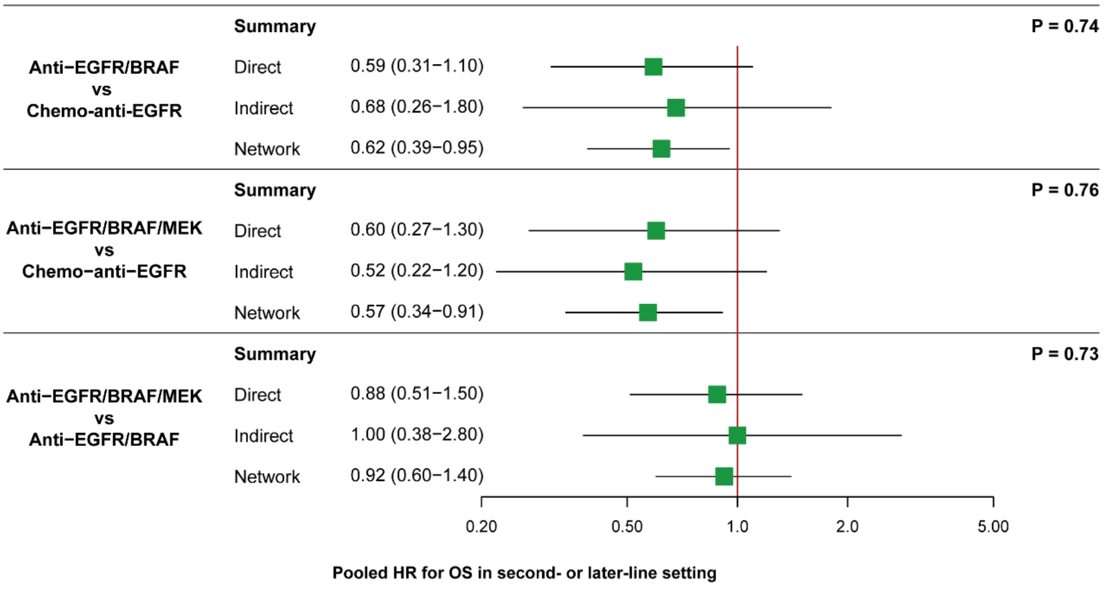
Summary
P = 0.74
Anti−EGFR/BRAF
vs
Chemo-anti-EGFR
Direct 0.59 (0.31−1.10)
Indirect 0.68 (0.26−1.80)
Network 0.62 (0.39−0.95)
Summary
P = 0.76
Anti−EGFR/BRAF/MEK
vs
Chemo−anti−EGFR
Direct 0.60 (0.27−1.30)
Indirect 0.52 (0.22−1.20)
Network 0.57 (0.34−0.91)
Summary
P = 0.73
Anti−EGFR/BRAF/MEK
vs
Anti−EGFR/BRAF
Direct 0.88 (0.51−1.50)
Indirect 1.00 (0.38−2.80)
Network 0.92 (0.60−1.40)
0.20
0.50
1.0
2.0
5.00
Pooled HR for OS in second- or later-line setting

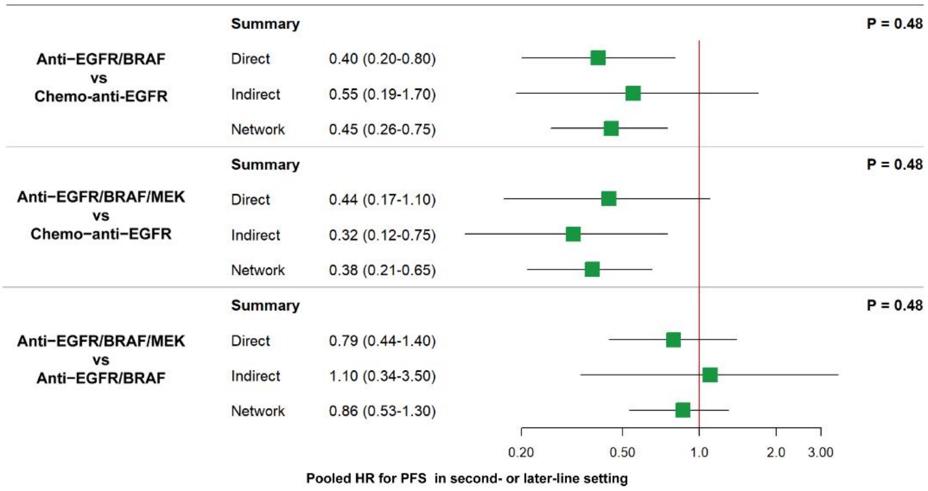
Summary
P = 0.48
Anti−EGFR/BRAF
vs
Chemo-anti-EGFR
Direct 0.40 (0.20-0.80)
Indirect 0.55 (0.19-1.70)
Network 0.45 (0.26-0.75)
Summary
P = 0.48
Anti−EGFR/BRAF/MEK
vs
Chemo−anti−EGFR
Direct 0.44 (0.17-1.10)
Indirect 0.32 (0.12-0.75)
Network 0.38 (0.21-0.65)
Summary
P = 0.48
Anti−EGFR/BRAF/MEK
vs
Anti−EGFR/BRAF
Direct 0.79 (0.44-1.40)
Indirect 1.10 (0.34-3.50)
Network 0.86 (0.53-1.30)
0.20
0.50
1.0
2.0
3.00
Pooled HR for PFS in second- or later-line setting

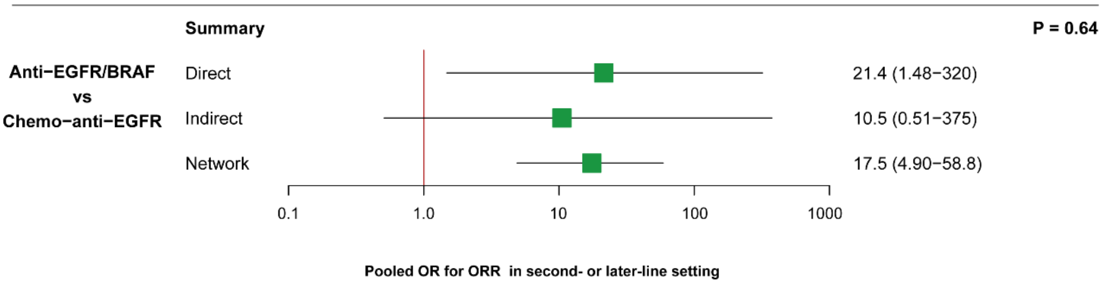
Summary
P = 0.64
Anti−EGFR/BRAF
vs
Chemo−anti−EGFR
Direct 21.4 (1.48−320)
Indirect 10.5 (0.51−375)
Network 17.5 (4.90−58.8)
0.1
1.0
10
100
1000
Pooled OR for ORR in second- or later-line setting

Figure S12. Risk of bias assessment for the included single-arm trials and non-randomised controlled trial according to the MINORS Evaluation Criteria[5].

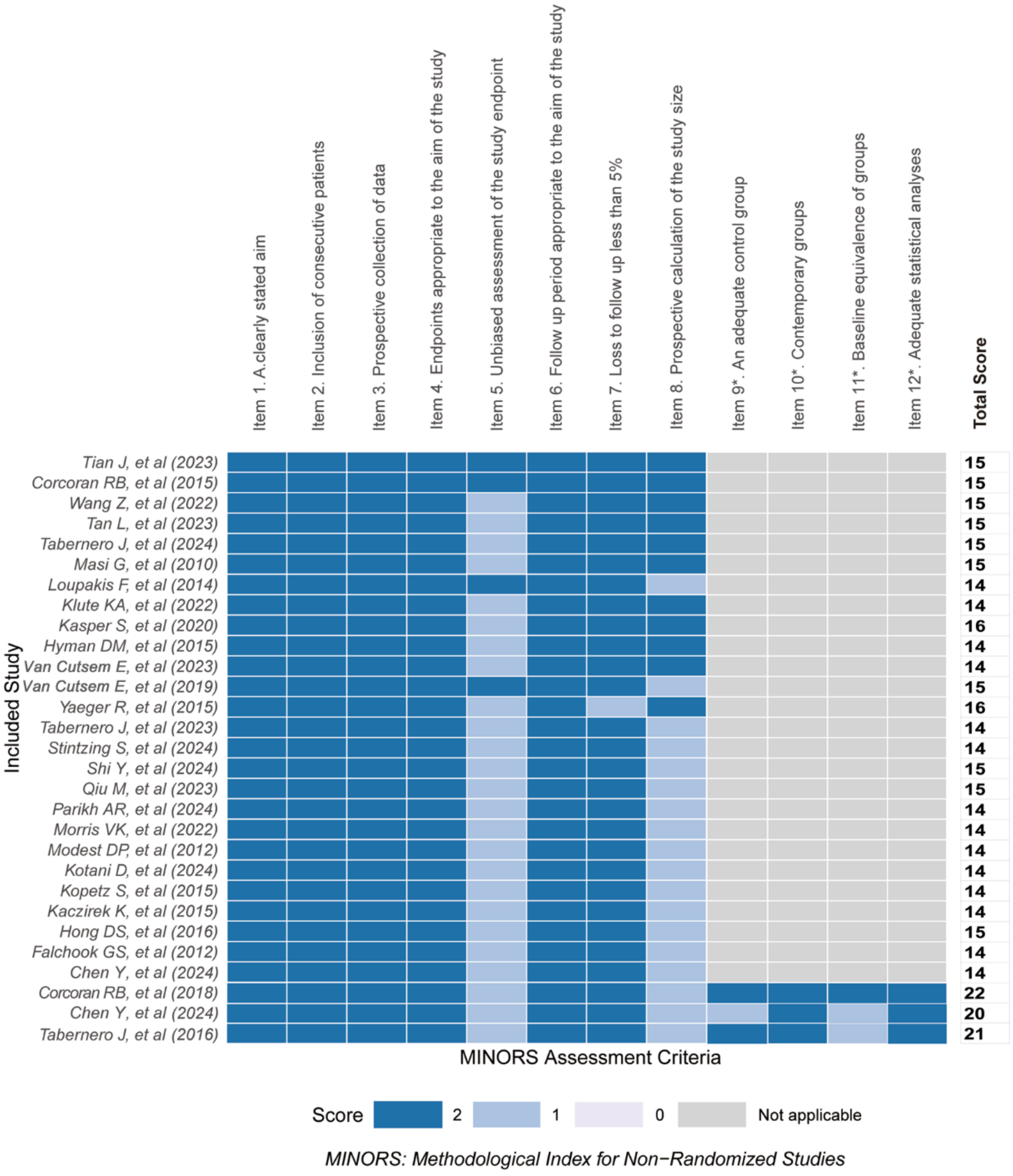

*For comparative studies only (Item 9-12).

Total scores of at least 75% were considered high quality with low risk for bias; scores between 50% and 75% were considered medium risk for bias; scores of less than or equal to 50% were considered high risk for bias. For noncomparative studies, the maximum score was 16, while the maximum score for comparative studies was 24.

Figure S13. Risk of bias assessments for the included randomised controlled trials according to the ROB2 tool[6].

Unique ID	Study ID	Experimental	Comparator	Outcome	D1	D2	D3	D4	D5	Overall
Schmoll HJ, et al (2024)	CHARTA	TCT-anti-VEGF	DCT-anti-VEGF	OS, PFS, ORR	!	+	+	+	+	+
Gruenberger T, et al (2015)	OLIVIA	TCT-anti-VEGF	DCT-anti-VEGF	OS, PFS, ORR	!	+	+	+	+	!
Hurwitz HI, et al (2019)	STEAM	TCT-anti-VEGF	DCT-anti-VEGF	OS, PFS, ORR	!	+	+	+	+	+
Cremolini C, et al (2015)	TRIBE	TCT-anti-VEGF	DCT-anti-VEGF	OS, PFS, ORR	!	+	+	+	+	+
Cremolini C, et al (2020)	TRIBE2	TCT-anti-VEGF	TCT-anti-VEGF vs DCT-anti-VEGF	OS, PFS, ORR	!	+	+	+	+	+
Bond MJG, et al (2023)	CAIRO5	TCT-anti-VEGF	DCT-anti-VEGF	OS, PFS, ORR	+	+	+	+	+	+
Maughan TS, et al (2011)	COIN	DCT-anti-EGFR	DCT	OS, PFS	!	+	+	+	+	+
Innocenti F, et al (2019)	CALGB80405	DCT-anti-EGFR/VEGF	DCT-anti-VEGF vs DCT-EGFR	OS, PFS	!	+	+	+	+	+
Van Cutsem E, et al (2009)	CRYSTAL	DCT-anti-VEGF	DCT	OS, PFS, ORR	!	+	+	+	+	+
Bokemeyer C, et al (2009)	OPUS	DCT-anti-VEGF	DCT	OS, PFS, ORR	!	+	+	+	+	+
Douillard JY, et al (2013)	PRIME	DCT-anti-EGFR	DCT-anti-VEGF	OS, PFS	!	+	+	+	+	+
Rivera F, et al (2017)	PEAK	DCT-anti-EGFR	DCT-anti-VEGF	OS, PFS, ORR	!	+	+	+	+	+
Stintzing S, et al (2023)	FIRE-4.5	TCT-anti-EGFR	TCT-anti-VEGF	OS, PFS, ORR, DCR, AE	+	+	+	+	+	+
Ten Hoorn S, et al (2022)	CAIRO2	DCT-anti-EGFR/VEGF	DCT-anti-VEGF	OS	!	+	+	+	+	+
Elez E, et al (2025)	BREAKWATER	DCT-anti-EGFR/BRAF	DCT-anti-VEGF	OS, ORR, AE	+	+	+	+	+	+
Stintzing S, et al (2017)	FIRE-3	TCT-anti-EGFR	TCT-anti-VEGF	OS, PFS, ORR, DCR	!	+	+	+	+	+
Tol J, et al (2009)	NA	DCT-anti-EGFR/VEGF	DCT-anti-VEGF	ORR	!	+	+	+	!	!
Tveit KM, et al 2012	NORDIC-VII	DCT-anti-EGFR	DCT-anti-EGFR vs DCT	ORR	!	+	+	+	+	+
Aranda E, et al (2020)	VISNÚ-1	TCT-anti-VEGF	DCT-anti-VEGF	PFS	!	+	+	+	+	+
Lopez-Crapez E, et al (2018)	METHEP2	DCT-anti-EGFR	TCT-anti-EGFR	ORR	!	+	+	+	+	!
Tabernero J, et al (2021)	BEACON	Anti-EGFR/BRAF/MEK	Anti-EGFR/BRAF vs Chemo-anti-EGFR	OS, PFS, ORR, DCR, AE	+	+	+	+	+	+
Tabernero J, et al (2016)	NA	Anti-EGFR/BRAF/PI3K	Anti-EGFR/BRAF	OS, PFS, ORR, AE	+	+	+	+	+	+
Seymour MT, et al (2013)	PICCOLO	Chemo-anti-EGFR	Chemo	OS, PFS, ORR	!	+	+	+	+	+
Peeters M, et al (2015)	20050181	Chemo-anti-EGFR	Chemo	OS, PFS	!	+	+	+	+	+
Kopetz S, et al (2021)	SWOG S1406	Chem-anti-EGFR/BRAF	Chem-EGFR	OS, PFS, ORR, DCR, AE	+	+	+	+	+	+
Wang X, et al (2024)	NAUTICAL CRC	Anti-EGFR/BRAF	Chemo-anti-EGFR	OS, PFS, ORR, AE	+	+	+	+	+	+

D1 Randomisation process
D2 Deviations from the intended interventions
D3 Missing outcome data
D4 Measurement of the outcome
D5 Selection of the reported result

+ Low risk
! Some concerns
- High risk

Figure S14. Risk of bias assessment for the included real-world study according to the ArRoWS Evaluation Criteria[7].

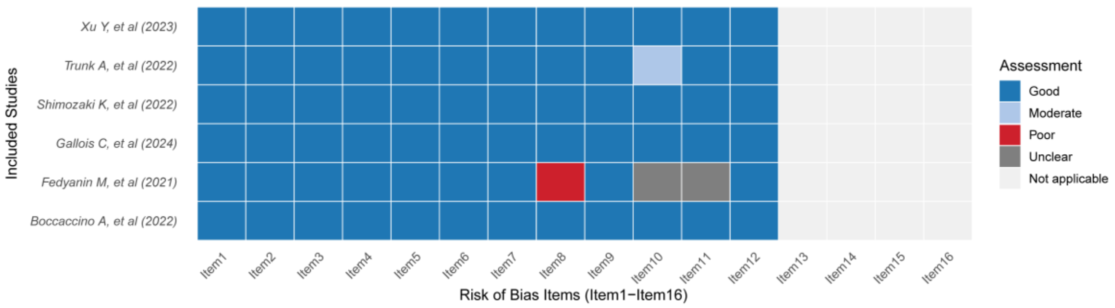

Figure S15. Risk of bias assessment for the included real-world study according to the ROBINS-I Evaluation Criteria[8].

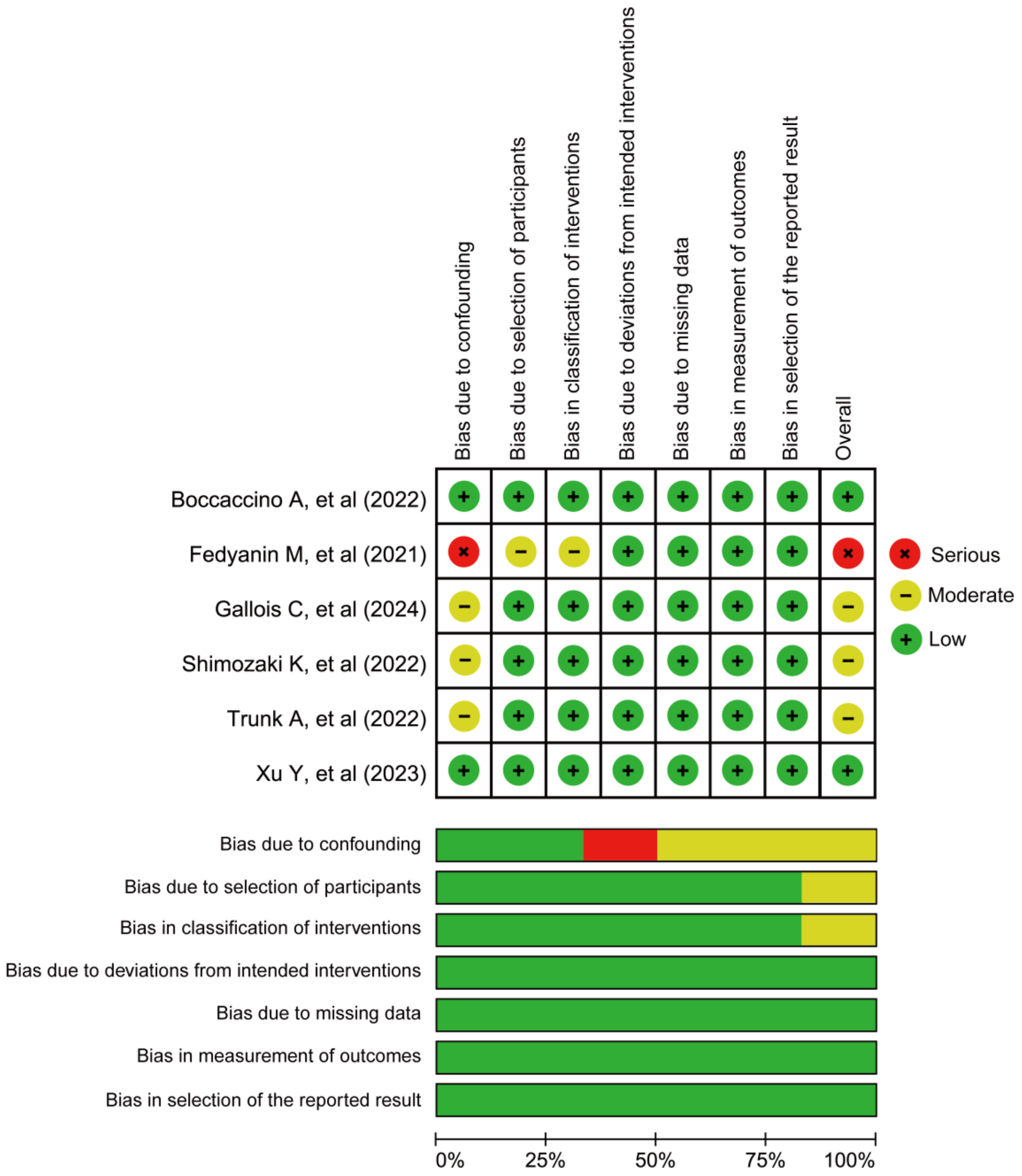

References:

1. Dias S, Sutton AJ, Welton NJ, et al. Evidence synthesis for decision making 3: heterogeneity--subgroups, meta-regression, bias, and bias-adjustment. *Med Decis Making* 2013;33(5):618-40. doi: 10.1177/0272989x13485157
2. Spiegelhalter DJ, Best NG, Carlin BP, et al. Bayesian Measures of Model Complexity and Fit. *Journal of the Royal Statistical Society Series B: Statistical Methodology* 2002;64(4):583-639. doi: 10.1111/1467-9868.00353
3. Nikolakopoulou A, Higgins JPT, Papakonstantinou T, et al. CINeMA: An approach for assessing confidence in the results of a network meta-analysis. *PLoS Med* 2020;17(4):e1003082. doi: 10.1371/journal.pmed.1003082 [published Online First: 20200403]
4. Brooks SP, and Gelman A. General Methods for Monitoring Convergence of Iterative Simulations. *Journal of Computational and Graphical Statistics* 1998;7(4):434-55. doi: 10.1080/10618600.1998.10474787
5. Slim K, Nini E, Forestier D, et al. Methodological index for non-randomized studies (minors): development and validation of a new instrument. *ANZ J Surg* 2003;73(9):712-6. doi: 10.1046/j.1445-2197.2003.02748.x
6. Sterne JAC, Savović J, Page MJ, et al. RoB 2: a revised tool for assessing risk of bias in randomised trials. *Bmj* 2019;366:l4898. doi: 10.1136/bmj.l4898 [published Online First: 20190828]
7. Coles B, Tyrer F, Hussein H, et al. Development, content validation, and reliability of the Assessment of Real-World Observational Studies (ArRoWS) critical appraisal tool. *Ann Epidemiol* 2021;55:57-63.e15. doi: 10.1016/j.annepidem.2020.09.014 [published Online First: 20201001]
8. Sterne JA, Hernán MA, Reeves BC, et al. ROBINS-I: a tool for assessing risk of bias in non-randomised studies of interventions. *Bmj* 2016;355:i4919. doi: 10.1136/bmj.i4919 [published Online First: 20161012]

Supplementary Note 1

Systematic Review Protocol

**Title:**	Targeted therapy in advanced BRAF-mutated colorectal cancer: a comprehensive systematic review and network meta-analysis
**Registration :**	PROSPERO CRD420250653959
**Review affiliation:**	Shanghai Changzheng Hospital
**Pricinple Investigator:**	Professor Bao-Dong Qin<br>Professor Xiao-Dong Jiao<br>Professor Yuan-Sheng Zang
**Contact:**	Professor Yuan-Sheng Zang<br>E-mail: doctorzangys@163.com<br>Address: No.415 Fengyang Road, Shanghai, 200003, Republic of China
**Protocol Version:**	2.0 version
**Data:**	June 10th, 2025

Confidentiality Statement:

This document and associated unpublished materials (including but not limited to: preliminary data analyses, unpublished data, draft manuscripts, privileged communications, and pre-publication findings) contain confidential information solely for the purpose of conducting this systematic review and meta-analysis.

Protocol Summary

**Title**	Targeted therapy in advanced BRAF-mutated colorectal cancer: a comprehensive systematic review and network meta-analysis
**Objectives**	To assess the efficacy and safety of targeted therapy regimens among advanced colorectal cancer patients harboring BRAF mutation
**Study Population**	Patients diagnosed with advanced BRAF-mutated colorectal cancer
**Reporting framework**	This systematic review, meta-analysis, and network meta-analysis were conducted using a predetermined protocol following the Preferred Reporting Items for Systematic Reviews and Meta-Analyses (PRISMA) extension statement for network meta-analyses
**Search database**	PubMed, EMBASE, Cochrane library, ClinicalTrials.gov as well as some important international conferences (e.g. American Society of Clinical Oncology, European Society for Medical Oncology, American Association for Cancer Research)
**Inclusion criteria**	Studies that met the following criteria were included:<br>1) published and unpublished phase I, II, and III clinical trials or multicentre, large-sample, real-world studies;<br>2) enrolling participants with histological or cytologically confirmed mCRC with BRAF mutation;<br>3) evaluating targeted-therapy based regimen on at least one of the clinical outcomes of interest, including overall survival (OS), progression-free survival (PFS), objective response rate (ORR), disease control rate (DCR), as well as grade ≥3 adverse events (Grade≥3 AEs) or sufficient data that could calculate these outcomes
**Exclusion criteria**	Other exclusion criteria were as follows: 1) studies including patients with advanced BRAFmut CRC but relevant data for the BRAF-mutant population were not independently reported; 2) trials that contained insufficient published data or original data for meta-analysis; and 3) the studies focused on populations with non-BRAF V600E mutations. Studies reporting updated data multiple times were

	included based on the most recent version.
**Language restriction**	English
**Publication data restriction**	31th May, 2025
**Outcomes**	Primary Endpoint: Overall Survival (OS)<br>Secondary Endpoints: Progression-Free Survival (PFS), Objective Response Rate (ORR), Disease Control Rate (DCR), and safety (grade 3 or higher adverse events).
**Data Presentation**	Hazard ratios (HRs) with 95% credible intervals (CIs) were used to quantify PFS and OS, whereas odds ratios (ORs) with 95% CIs were used to assess ORR, DCR, and grade≥3 AEs.
**Evaluation of Heterogeneity**	Heterogeneity across studies was evaluated using the $\chi 2$-based Q test and was quantified using the inconsistency index (I^2) test. Random-effects models were implemented when $I^2>50\%$ or Q-test $p<0.10$. Otherwise, fixed-effects models were used.
**Data synthesis methods**	Three core methodological methods were used as follows: 1) A single-arm meta-analysis was performed to synthesize pooled estimates for ORR, DCR, and grade≥3 AEs occurrence rates for each regimen, based on all available arm-level data from the included studies reporting quantifiable outcome measures. 2) Pairwise meta-analyses were restricted to direct comparisons between two or more controlled trials. 3) Network meta-analysis was performed in a Bayesian framework, comparing any two targeted therapy-based strategies by simultaneously synthesizing direct and indirect evidence.
**Core Results Display**	Forest Plots for single-arm meta-analysis and pairwise meta-analysis; Matrix diagram, Bayesian ranking plots, cumulative ranking plots for Network meta-analysis
**Other analysis**	Meta-regression analysis; Sensitivity analysis; Trace plots; Density plots; the Brooks–Gelman–Rubin method; the fit goodness between consistency and inconsistency models based on DIC values; the node-splitting method.
**Statistical analysis**	Data analyses were performed in R (version 4.3.2) by implementing three core methodologies: single-arm proportion synthesis and pairwise meta-analyses using the meta package, and Bayesian network meta-analysis using gemtc package.

Contents

1. BACKGROUND 1

2. OBJECTIVE OF THIS SYSTEMATIC REVIEW 2

2.1 Specify the disease/health problem of interest..2

2.2 Specify the population studied...2

2.3 Specify the intervention/exposure 2

2.4 specify the control population..3

2.5 state your research question 3

3. METHOD 3

3.1 Study guidance...3

3.2 Search and study identification..3

3.2.1 Identify literature database 3

3.2.2 Define electronic search strategy...4

3.2.3 Study selection procedure 5

3.2.4 Specify number of observers per screening phase.....................................5

3.2.5 De-duplication 6

3.3 Study Selection criteria...6

3.3.1 Type of study design..6

3.3.2 Study Selection..6

3.3.3 Outcome measures of interest 6

3.3.4 Language restrictions 6

3.3.5 Publication data restrictions: 7

3.3.6 Exclusion criteria per screening phase 7

4. STUDY CHARACTERISTICS TO BE EXTRACTED 7

4.1 Study identification...7

4.2 Study design characteristics..7

4.3 Publication type 7

4.4 Patients characteristics...7

4.4.1 Treatment background 7
4.4.2 BRAF mutation type 8
4.4.3 Data source 8
4.4.4 Sample size of each included study 8
4.4.5 Intervention characteristics 9
4.4.6 Data collection 10
4.4.7 Methods of data extraction and retrieval 11
5. DATA ANALYSIS AND DATA SYNTHESIS 11
5.1 Meta-analysis methods 11
5.1.1 single-arm meta-analysis 12
5.1.2 Pairwise meta-analysis 12
5.1.3 Network meta-analysis 12
5.2 Data presentation 12
4.3 Visualization of the key outcomes 12
5.4 Risk of bias assessment of each included study 12
5.5 Statistical analysis 13
5.6 Model selection 13
5.7 Other analysis 13
6. STRENGTHS AND INNOVATIONS 14
7. POSSIBLE RISK 14
8. Conflict of Interest 14
9.Reference 15

1. BACKGROUND

Colorectal cancer (CRC) is the most common malignant tumors in the digestive system, which is a serious threat to human health[1]. Almost 1/4 patients were developed advanced stage at the time of diagnosed, half of CRC patients could develop recurrence and distant metastasis, resulting in poor prognoses and short survival[2]. For advanced or metastatic CRC, chemotherapy is one of the main treatment methods, which plays an important role in controlling the disease, prolonging the survival period and improving the quality of life. There are three commonly used chemotherapy drugs: fluorouracil, oxaliplatin and irinotecan. The researches have not made breakthrough progress on other chemotherapy drugs. In addition, targeted therapy also plays an important role in the treatment of advanced CRC. At present, the combination of chemotherapy and targeted therapy (anti-VEGF or anti-EGFR) is the standard-of-care recommended by NCCN or ESMO guidelines[3 4]. Recently, the rise of precision medicine dominated by genotyping provides a new field and direction for targeted therapy. Meanwhile, precision medicine also provides some treatment strategies for refractory CRC including BRAF mutated CRC.

BRAF is one of the most important oncogenes in human, and about 8% of human tumors harbor BRAF mutation. BRAF V600E mutation is the most common BRAF mutation, which could be found mainly in melanoma, colon and thyroid cancer. This mutation could induce the activation of downstream MEK-ERK signaling pathway, which is very important for the growth, proliferation and invasion and metastasis of cancer cells. The incidence of BRAF V600E mutation in CRC (BRAFmut CRC) is approximately 8-15%, which is more common among CRC patients with right colon lesion, lymph node and peritoneal metastasis, and less common in CRC patients with lung metastasis. The prognosis of BRAFmut CRC patients is poor. If using the traditional standard treatment, the median progression free survival (PFS) of first-line therapy is 6 months, the median PFS of second-line therapy is only 2-3 months, and the overall survival (OS) is only 10 months. In contrast, the OS of advanced CRC patients with RAS/RAF wild type can reach 30 months. Thus, it is urgent to find effective regimen for BRAFmut CRC patients.

Owing to the limited efficacy of conventional therapeutic strategies for BRAFmut CRC, BRAF-targeted therapies have been explored to improve therapeutic efficacy. However, BRAF inhibitor monotherapy has not yielded significant clinical improvements[5 6], likely due to

compensatory feedback reactivation of EGFR-mediated mitogen-activated protein kinase signalling pathways, a well-documented resistance mechanism in preclinical models[7 8]. Subsequently, the finding of EGFR-mediated feedback activation has driven the exploration of dual EGFR/BRAF blockade in BRAFmut CRC. Trials, including BEACON, have demonstrated the efficacy of EGFR/BRAF co-inhibition in second-line settings, whereas BREAKWATER and SWOG1406 further established the superiority of anti-EGFR/BRAF regimens combined with chemotherapy in both first- and second-line treatments[9-11]. However, critical uncertainties persist due to the lack of direct comparisons between emerging therapeutic strategies.

Therefore, we conducted the present systematic review and meta-analysis to comprehensively investigate the individual and comparative efficacy and safety of targeted therapy-based strategies in advanced BRAFmut CRC.

2. OBJECTIVE OF THIS SYSTEMATIC REVIEW

2.1 Specify the disease/health problem of interest

In the current systematic review, we have included studies that satisfy the following definition: advanced colorectal cancer with BRAF mutation (AJCC-TNM Stage IV).

2.2 Specify the population studied

In the current systematic review, we will focus on patients diagnosed with advanced colorectal cancer who harboring BRAF mutation. Animal studies, preclinical research were not eligible. All eligible patients must be advanced or metastatic colorectal cancer. Patients with molecularly confirmed BRAF mutations receiving advanced systemic anticancer therapy were included. Studies investigating early-stage interventions (including surgical resection, neoadjuvant therapy, or adjuvant therapy) for patients with BRAF mutations were excluded.

2.3 Specify the intervention/exposure

The intervention applied for the treatment of advanced BRAFmut CRCs should be anti-tumor drugs therapy in current systematic review, mainly including targeted-therapy combinations. Anti-EGFR and anti-VEGF therapies represent established backbone treatments for advanced colorectal cancer. Doublet or triplet chemotherapy regimens remain cornerstone cytotoxic approaches. Recently, pathway-directed agents targeting downstream EGFR signaling—specifically BRAF inhibitors and MEK inhibitors—have been integrated into management algorithms for advanced

BRAFmut CRC. Collectively, these targeted therapeutic strategies constitute the investigational interventions eligible for inclusion in this systematic review.

2.4 specify the control population

This systematic review did not pre-specify control groups. All retrieved treatment regimens will undergo comprehensive efficacy and safety analyses. Where feasible, both direct comparisons (head-to-head pairwise meta-analyses) and indirect comparisons (network meta-analyses) will be employed to evaluate comparative efficacy and safety profiles across interventions, thereby achieving the study's primary objective.

2.5 state your research question

There are several critical uncertainties persist due to the lack of direct comparisons between emerging therapeutic strategies among BRAFmut CRC patients, including: 1) Is the targeted therapy necessary for improving clinical outcomes among BRAFmut CRC patients, or whether novel targeted therapy-based strategies are better than traditional regimens? 2) There are several combinations targeting VEGF, EGFR signalling pathway, which can optimize outcomes? 3) Is targeted therapy alone sufficient to achieve therapeutic efficacy? Should targeted therapy be combined with chemotherapy, and if so, with single-agent or doublet chemotherapy? 4) Does earlier initiation of targeted-based therapy in the first-line setting confer superior survival compared to later-line therapy? 5) What is the safety profile of targeted therapy-based regimens, or do targeted therapy-based regimens increase toxicity while maintaining acceptable safety? Thus, we will conduct this systematic review and meta-analysis to answer the above question.

3. METHOD

3.1 Study guidance

This systematic review and meta-analysis would be conducted using a predetermined protocol following the Preferred Reporting Items for Systematic Reviews and Meta-Analyses (PRISMA) extension statement for network meta-analyses [12].

3.2 Search and study identification

3.2.1 Identify literature database

A systematic literature search of electronic databases, including PubMed, EMBASE, Cochrane library, and ClinicalTrials.gov would be performed for eligible studies investigating the efficacy and

safety of targeted therapy-based strategies for advanced BRAFmut CRC. Abstracts and presentations from some important international conferences, including the American Society of Clinical Oncology, European Society for Medical Oncology, and the American Association for Cancer Research, would be also searched.

3.2.2 Define electronic search strategy

The detailed search strategies for PubMed, Embase, Cochrane Library, ClinicalTrials.gov were shown in Table 1. Conference abstracts and presentations were manually retrieved from the official websites of major relevant conferences. The multiple rounds of verification and cross-checking of these search strategy were conducted with our research team members Zhan Wang and Ke Liu.

Table 1: Literature search strategy.

**ID**	**Search**
**PubMed**	
#1	"Colorectal Neoplasms"[MeSH Terms] OR "colorectal cancer"[Title/Abstract]
#2	"intestinal neoplasms"[MeSH Terms] OR "colonic neoplasms"[MeSH Terms] OR "rectal neoplasms"[MeSH Terms]
#3	"bowel cancer"[Title/Abstract] OR "colon cancer"[Title/Abstract] OR "rectal cancer"[Title/Abstract]
#4	#1 OR #2 OR #3
#5	"Proto-Oncogene Proteins B-raf"[MeSH Terms] OR "braf"[All Fields] OR "braf"[Title/Abstract]
#6	"clinical trial"[Publication Type] OR "randomized controlled trial"[Publication Type] OR "trial"[Title/Abstract] OR "randomized"[Title/Abstract] OR "randomised"[Title/Abstract] OR "randomly"[Title/Abstract] OR (("real-world"[All Fields] AND ("studies"[All Fields] OR "study"[All Fields] OR "study s"[All Fields] OR "studying"[All Fields] OR "studys"[All Fields])) OR "real world study"[Title/Abstract] OR "real world cohort"[Title/Abstract] OR ("real"[All Fields] AND ("world"[All Fields] OR "worlds"[All Fields] OR "worlds"[All Fields]) AND ("cohort"[All Fields] OR "cohort s"[All Fields] OR "cohorte"[All Fields] OR "cohorts"[All Fields])))
#7	0001/01/01:2025/05/31[Date - Publication]
#8	#1 and #2 and #3 and #4
#9	#1 and #2 and #3 and #4 Filters: English
**Embase**	

#1	'colorectal cancer'/exp OR 'colorectal cancer':ab,ti OR 'intestine cancer'/exp OR 'intestine cancer':ab,ti OR 'colon cancer'/exp OR 'colon cancer':ab,ti OR 'rectal cancer'/exp OR 'rectal cancer':ab,ti
#2	'B Raf kinase'/exp OR 'BRAF':ab,ti
#3	'clinical trial'/exp OR 'clinical study'/exp OR 'controlled clinical trial'/exp OR 'randomized controlled trial'/exp OR 'real world study'/exp OR 'real world data'/exp OR 'real world evidence'/exp OR 'clinical trial' OR 'real world study' OR 'real world data' OR 'real world evidence' OR 'clinical study' OR 'clinical trial'
#4	'gene mutation'/exp OR 'mutation':ab,ti
#5	'patient'/exp OR 'patient':ab,ti OR 'patients':ab,ti
#6	'therapy'/exp OR 'treatment'/exp
#7	#1 and #2 and #3 and #4 and #5 and #6 [english]/lim
#8	#1 and #2 and #3 and #4 and #5 and #6 [english]/lim AND [01-01-0001]/sd NOT [31-05-2025]/sd
**Cochrane Library**	
#1	'colorectal cancer [MeSH]' OR (colorectal cancer):ab,ti,kw OR (bowel cancer):ab,ti,kw OR (colon cancer):ab,ti,kw OR (rectal cancer):ab,ti,kw
#2	'BRAF [MeSH]' OR (BRAF):ab,ti,kw
#3	'mutation [MeSH]' OR (mutation):ab,ti,kw
#4	#1 and #2 and #3
#5	#4 Cochrane Library publication date Between Jan 0001 and May 2025, in Trials
**ClinicalTrials.gov**	
#1	"colorectal cancer" OR "colorectal carcinoma" OR "colo-rectal cancer" OR "colorectal cancers" OR "bowel cancer" OR "colon cancer" OR "rectal cancer" | Other terms: BRAF Gene Mutation

3.2.3 Study selection procedure

Study selection procedure includes two screening phases: 1) Title/abstract screening phase; 2) Full-text screening phase.

3.2.4 Specify number of observers per screening phase

Two independent investigators (Bao-Dong Qin and Xiao-Dong Jiao) would screen the titles and abstracts, and the full texts of potentially eligible studies sequentially. Any disagreements would be resolved by a third investigator (Yuan-Sheng Zang).

3.2.5 De-duplication

Literature retrieved from PubMed, EMBASE, and Cochrane Library will be exported in EndNote format. Duplicate records will be removed using EndNote's automated deduplication feature based on matching titles, authors, and publication years. Manual deduplication will be performed for records from ClinicalTrials.gov and conference abstracts through independent dual-reviewer verification.

3.3 Study Selection criteria

3.3.1 Type of study design

Phase I, Phase II, Phase III clinical trials are eligible, as well as the multicentre real-world studies. Review, case reports, case series could be not included.

3.3.2 Study Selection

The study must meet these following inclusion criteria could be included. Inclusion criteria: 1) published and unpublished phase I, II, and III clinical trials or multicentre, large-sample, real-world studies; 2) enrolling participants with histological or cytologically confirmed mCRC with *BRAF* mutation; 3) evaluating targeted-therapy based regimen on at least one of the clinical outcomes of interest, including overall survival (OS), progression-free survival (PFS), objective response rate (ORR), disease control rate (DCR), as well as grade ≥3 adverse events (Grade≥3 AEs) or sufficient data that could calculate these outcomes[13 14]. Meanwhile, the studies meet the following exclusion criteria could be excluded. Exclusion criteria: 1) studies including patients with advanced BRAFmut CRC but relevant data for the BRAF-mutant population were not independently reported; 2) trials that contained insufficient published data or original data for meta-analysis; and 3) the studies focused on populations with non-BRAF V600E mutations.

3.3.3 Outcome measures of interest

Primary endpoint of interest is overall survival (OS), while secondary endpoint of interest included progression-free survival (PFS), objective response rate (ORR), disease control rate (DCR), grade 3 or higher adverse events.

3.3.4 Language restrictions

The literature search for the current systematic review was limited to the English language.

3.3.5 Publication data restrictions:

The publication date of literature search was set to May 31, 2025.

3.3.6 Exclusion criteria per screening phase

The selection phases for eligible studies included title/abstract screening phase and full text selection phase. a) Exclusion criteria in title/abstract screening phase included: a) no colorectal cancer; b) no anti-tumor therapy; c) No original data (e.g. review, case reports, etc.); d) preclinical study; e) basic mechanism research. B) Exclusion criteria in title/abstract screening phase included: a) no advanced colorectal cancer (e.g. early-stage patients); b) no BRAF mutation; c) no reported data associated with any endpoint of interest; d) no sufficient data for calculating the endpoint of interest; e) no anti-tumor drug therapy (e.g. surgery, radiotherapy, cell therapy, cancer vaccines, etc.); f) no first-line, second- or later-line therapy (e.g. neoadjuvant therapy, adjuvant therapy, etc.).

4. STUDY CHARACTERISTICS TO BE EXTRACTED

4.1 Study identification

Study identification data were extracted including first author, publication year, the country of where the study was conducted, trial registration, trial identification, published literature or conference data.

4.2 Study design characteristics

The published and unpublished phase I, II, and III clinical trials or multicentre, large-sample, real-world studies were included.

4.3 Publication type

(Published literature vs Conference report): categorized by publication types (Published literature indicated peer-reviewed full-text articles in indexed journals, as opposed to conference reports containing available data from conference abstract or presentations)

4.4 Patients characteristics

4.4.1 Treatment background

Patient treatment setting will be categorized as first-line therapy and second-line or later therapy (second-line and beyond). It is recognized that in some studies, cohorts described as receiving second-line therapy may potentially include patients who received third-line or subsequent therapies. Then, the cohorts were divided into two groups: first-line setting, second- or later-line

setting.

4.4.2 BRAF mutation type

The BRAF V600E mutation represents the most common BRAF mutation subtype in advanced colorectal cancer patients. Based on our prior review of existing literature, we recognize that some prior studies may have included cohorts containing a small proportion of non-V600E BRAF mutations. Data extraction strategy regarding BRAF mutation status: 1) preference will be given to extracting data specifically from the BRAF V600E mutant subgroup whenever reported separately within a study; b) if a study only reports combined data for all BRAF mutations, without data on isolating V600E, the overall BRAF mutant cohort data will be extracted. Then, studies providing only combined BRAF mutation data will be classified into a distinct group: Mixed mutation group (defined as cohorts containing a small proportion of non-V600E mutations). This will allow for comparison against the BRAF V600E group (cohorts confirmed to harbor only the V600E mutation) in subsequent analyses.

4.4.3 Data source

Historically, BRAF mutations are observed in approximately 8-15% of all patients with advanced colorectal cancer. Consequently, in earlier studies, BRAF-mutant populations were typically analyzed only as a subgroup within advanced colorectal cancer cohorts when exploring the efficacy of safety of anti-cancer therapy among advanced colorectal cancer patients. With advancements in and widespread adoption of genetic testing technologies, an increasing number of patients with advanced BRAF-mutant colorectal cancer are being identified, leading to greater research focus on this specific population. Recent studies now frequently focus on BRAF-mutant CRC as a distinct cohort.

To distinguish the level of evidence based on study population characteristics: Studies which all enrolled patients had confirmed BRAF mutations will be classified as providing "Trial-Level Evidence". Studies which BRAF-mutant patients constituted a specifically defined subgroup** within a larger cohort will be classified as providing "Subgroup-Level Evidence".

4.4.4 Sample size of each included study

The number of advanced BRAFmut colorectal cancer patients from each included study was also extracted.

4.4.5 Intervention characteristics

We will comprehensively search for all currently reported therapeutic regimens used in advanced BRAF-mutant colorectal cancer patients across first-line, second- or later-line therapy. Potential treatment classification plan was as follows: Regimens will be categorized into **three primary classes: a) chemotherapy; b) targeted therapy; c) targeted therapy combinations (e.g. chemotherapy plus targeted therapy). Recognizing that chemotherapy alone has historically been used less frequently in this specific population, this study will intentionally focus on targeted therapy and targeted combination regimens. However, studies involving chemotherapy monotherapy will still be included to enable the comparative analysis via network meta-analysis (NMA).

Table 2: The detailed classification framework is outlined in the following table.

Chemotherapy category	e.g.<br>1. Single-agent chemotherapy (SCT);<br>2. Doublet chemotherapy (DCT);<br>3. Triplet chemotherapy (TCT);<br>4. etc.
Targeted therapy category	e.g.<br>1. EGFR inhibitor (anti-EGFR);<br>2. VEGF inhibitor (anti-VEGF);<br>3. BRAF inhibitor (anti-BRAF);<br>4. MEK inhibitor (anti-MEK);<br>5. PI3K inhibitor (anti-PI3K);<br>6. ERK inhibitor (anti-ERK);<br>7. WNT inhibitor (anti-WNT)<br>8. PD-1 inhibitor (anti-PD-1)<br>9. etc.
Combination strategies in first-line setting	e.g.<br>1. EGFR inhibitor-based combinations including:<br>Doublet chemotherapy-anti-EGFR (DCT-anti-EGFR);<br>Triplet chemotherapy-anti-EGFR (TCT-anti-EGFR);<br>2. VEGF inhibitor-based combinations including:<br>Doublet chemotherapy-anti-VEGF (DCT-anti-VEGF;<br>Triplet chemotherapy-anti-VEGF (TCT-anti-VEGF);<br>3. EGFR/VEGF inhibitors-based combinations including:<br>Doublet chemotherapy-anti-EGFR/VEGF(DCT-anti-EGFR/VEGF)

	4. EGFR/BRAF inhibitor-based combinations including:<br>Single-agent chemotherapy-anti-EGFR/BRAF (SCT-anti-EGFR/BRAF)<br>Doublet chemotherapy-anti-EGFR/BRAF (DCT-anti-EGFR/BRAF);<br>5.Targeted therapy alone regimens including:<br>Anti-EGFR/BRAF<br>Anti-EGFR/BRAF/MEK<br>6. etc.
Combination strategies in second- or later-line setting	e.g.<br>1. Chemotherapy plus EGFR inhibitor (Chemotherapy-anti-EGFR);<br>2. Chemotherapy plus EGFR/BRAF inhibitor (chemotherapy-anti-EGFR/BRAF)<br>3. Chemotherapy plus EGFR/BRAF/MEK inhibitors (chemotherapy-anti-EGFR/BRAF/MEK),<br>4. EGFR/MEK inhibitors (anti-EGFR/MEK),<br>5. EGFR/BRAF inhibitors (anti-EGFR/BRAF),<br>6. EGFR/BRAF/MEK inhibitors (anti-EGFR/BRAF/MEK),<br>7. EGFR/BRAF/PI3K inhibitors (anti-EGFR/BRAF/PI3K),<br>8. EGFR/BRAF/ERK inhibitors (anti-EGFR/BRAF/ERK),<br>9. EGFR/BRAF/WNT inhibitor (anti-EGFR/BRAF/WNT),<br>10. EGFR/BRAF/PD-1 inhibitors (anti-EGFR/BRAF/PD-1),<br>11.BRAF/MEK/PD-1 inhibitors (anti-BRAF/MEK/PD-1).<br>12. etc

In addition, if studies investigating second- or later-line therapies include a minimal proportion of treatment-naïve patients, we would stratify them into two distinct cohorts: uniform-exposure cohort: exclusively patients with documented prior systemic therapy; and mixed-exposure cohort: predominantly pretreated population with minimal treatment-naïve individuals.

4.4.6 Data collection

Data on endpoint of interest were extracted (HRs for OS and PFS, and the number of participants with ORR, DCR, and grade≥3 AEs). Several requirements were applied during data extraction: 1) we preferred the blinded independent central review (BICR)-assessed data derived from the intention-to-treat population; 2) the latest updated data from repeated reports of individual trials with varying follow-up periods were prioritized for inclusion; 3) targeted therapy-based regimen with fewer than 10 patients were deemed insufficient for robust statistical analysis; 4) the data from single-arm studies were exclusively used for single-arm meta-analyses of ORR, DCR,

and grade≥3 AEs, while they were not incorporated into pairwise meta-analysis or network meta-analyses as these studies lack comparator arms.

4.4.7 Methods of data extraction and retrieval

Two investigators (Bao-Dong Qin and Xiao-Dong Jiao) will independently extract data from the included studies using a prespecified extraction spreadsheet. Any discrepancies will be resolved through discussion with another investigators.

Table 3: Key data items extracted from each study

Study characteristics	first author, publication year, the country of where the study was conducted, trial registration, trial identification, published literature or conference data, PMID number if feasible, etc.
Methodological elements	Study design, sample size, etc.
Participant characteristics	Treatment background, BRAF mutation type, data source, etc
Intervention details	Line-based category, treatment regimen details, specific therapeutic agents, therapeutic regimen category, combination type, regimens comparison, etc.
Outcome measures	Endpoints of interest: PFS, OS, ORR, DCR, grade>3 or higher AEs; If feasible, HRs with 95%CI would be extracted for OS and PFS, as well as the number of participants with ORR, DCR, and grade≥3 AEs. If no available analysis data, the raw data would be extracted.
Risk-of-bias assessment	Each included study would be evaluated using corresponding assessment tools.

5. DATA ANALYSIS AND DATA SYNTHESIS

5.1 Meta-analysis methods

We will employ three distinct meta-analysis methods, selected based on the characteristics of the available data, to conduct this study: single-arm meta-analysis, pairwise meta-analysis, and network meta-analysis.

5.1.1 single-arm meta-analysis

Single-arm meta-analysis would be used to pooled the binary variable data including ORR, DCR, grade-3 or higher AEs from each single-arm studies and comparative studies, to estimate the pooled ORR, DCR, grade-3 or higher AEs of each regimen.

5.1.2 Pairwise meta-analysis

Pairwise meta-analysis would be used to perform direct comparisons of specific regimen based on the existing head-to-head trials including OS, PFS, ORR, DCR, AE. Certain studies may not be feasible due to insufficient sample size.

5.1.3 Network meta-analysis

Network meta-analysis would be used to perform indirect comparisons of all regimens based on the existing head-to-head trials including OS, PFS, ORR, DCR, AE. Certain studies may not be feasible due to insufficient sample size.

5.2 Data presentation

For single-arm meta-analysis: pooled ORR, DCR, grade 3 or higher AEs risk with 95%CI;

For pairwise meta-analysis: odds ratio with 95%CI for binary variable (e.g. ORR, DCR, grade 3 or higher AEs) when comparing specific regimens; Hazards ratio with 95%CI for survival data (e.g. OS and PFS) when comparing specific regimens.

For Network meta-analysis: odds ratio with 95%CI for ORR, DCR, grade 3 or higher AEs, hazard ratio with 95%CI for PFS and OS for all regimens comparison.

4.3 Visualization of the key outcomes

Forest plots would be used to visualize the main results from single-arm meta-analysis and pairwise meta-analysis. For Network meta-analysis, key visualizations will include the network plot, rankograms, cumulative ranking curves (e.g., SUCRA), and related NMA diagnostic graphs, etc.

5.4 Risk of bias assessment of each included study

The methodological quality of the included studies would be rigorously evaluated using three validated assessment tools: the Cochrane Risk of Bias tool version 2 (RoB 2.0) for randomized controlled trials[15], the Methodological Index for Non-randomised Studies (MINORS) for single-arm interventional studies and non-randomised controlled trials[16], the Assessment of Real-World Observational Studies (ArRoWS) framework and the Risk of bias in nonrandomized studies of

interventions version 2 (ROBINS-I V2) for real-world evidence investigations[17].

5.5 Statistical analysis

Data analyses were performed in R (version 4.3.2) by implementing three core methodologies: single-arm proportion synthesis and pairwise meta-analyses using the meta package, Bayesian network meta-analysis using gemtc package.

5.6 Model selection

Heterogeneity across studies was evaluated using the $\chi 2$-based Q test and was quantified using the inconsistency index (I^2) test. Random-effects models were implemented when I^2>50% or Q-test p<0.10. Otherwise, fixed-effects models were used. When conducting network meta-analysis, the consistency or inconsistency model were selected based on the Deviance Information Criterion (DIC) values. Lower DIC value indicates superior model fit. When comparing models, a DIC difference exceeding 5 warrants selection of the model with the lower DIC value.

5.7 Other analysis

Studies with strict patient allocation and an optimized balance for the same conditions would be included to ensure the transitivity of the key assumptions underlying the network meta-analysis. The transitivity assumption underlying indirect comparisons was evaluated using Bayesian meta-regression analyses, which examined the potential modifying effects of covariates, including sample size (Number of patients), study design (clinical trials versus real-world studies), data source (Trial-level data versus Subgroup-level data), mutation type (BRAF V600E versus Mixed type), prior therapy line (Uniform-exposure group vs Mixed-exposure group), data evidence (Published literature vs Conference report). Inconsistency was evaluated locally by comparing the pooled estimates from network and pairwise meta-analyses and globally by comparing the fit and parsimony of consistency and inconsistency models using the deviance information criterion (DIC) value, respectively[18]. Lower Deviance Information Criterion (DIC) values indicate superior model fit. When comparing models, a DIC difference exceeding 5 warrants selection of the model with the lower DIC value. Inconsistencies in the entire network were also assessed using the node-splitting method, in which direct and indirect evidence were compared separately. The convergence of the modes was evaluated using trace plots, density plots, and the Brooks–Gelman–Rubin method.

6. STRENGTHS AND INNOVATIONS

Previous meta-analyses were constrained by narrow therapeutic contexts (e.g., first-line comparisons of anti-VEGF vs. anti-EGFR regimens), small sample sizes, and the exclusion of emerging therapeutic strategies such as dual/triple target inhibition[1920]. Our study would address these limitations through several key innovations. For example, we would incorporate the most recent data covering new and updated data, enabling comprehensive evaluation of both first-line and subsequent-line targeted therapies, including novel therapeutic strategies, such as chemo-anti-EGFR/BRAF and targeted immunotherapy, reflecting current clinical trial priorities. Most importantly, robustness and reliability are enhanced by integrating data from RCTs, single-arm trials, and high-quality real-world evidence using diverse meta-analytical methods.

7. POSSIBLE RISK

First, due to the relatively limited number of studies specifically investigating BRAF-mutated colorectal cancer and the dispersed nature of treatment approaches, the number of eligible studies available for inclusion may be low. This scarcity could compromise the stability and robustness of our overall conclusions. To mitigate this risk, we will employ a broad inclusion strategy encompassing various study designs. Specifically, we will include Phase I, Phase II, and Phase III clinical trials, as well as real-world evidence (RWE) studies. This approach aims to maximize the pool of relevant data and enhance the reliability of our findings.

Second, the deliberate inclusion of diverse study types (e.g., RCTs alongside observational RWE studies) may introduce significant clinical and methodological heterogeneity. Furthermore, if the total number of included studies remains insufficient, it will preclude meaningful subgroup analyses and sensitivity analyses. These limitations could obscure nuanced effects and reduce confidence in the robustness of the pooled results. To address heterogeneity and explore the influence of potential effect modifiers (e.g., study design, line of therapy, specific regimens) despite the anticipated limitations on subgroup analysis, we will utilize meta-regression. This analytical method will allow us to investigate and quantify the impact of these covariates on the primary outcome estimates.

8. Conflict of Interest

All investigators declared no conflict of interest.

9.Reference

1. Bray F, Laversanne M, Sung H, et al. Global cancer statistics 2022: GLOBOCAN estimates of incidence and mortality worldwide for 36 cancers in 185 countries. *CA Cancer J Clin* 2024;74(3):229-63. doi: 10.3322/caac.21834 [published Online First: 20240404]
2. Van Cutsem E, Cervantes A, Nordlinger B, et al. Metastatic colorectal cancer: ESMO Clinical Practice Guidelines for diagnosis, treatment and follow-up. *Ann Oncol* 2014;25 Suppl 3:iii1-9. doi: 10.1093/annonc/mdu260 [published Online First: 20140904]
3. Cervantes A, Adam R, Roselló S, et al. Metastatic colorectal cancer: ESMO Clinical Practice Guideline for diagnosis, treatment and follow-up. *Ann Oncol* 2023;34(1):10-32. doi: 10.1016/j.annonc.2022.10.003 [published Online First: 20221025]
4. Benson AB, Venook AP, Adam M, et al. Colon Cancer, Version 3.2024, NCCN Clinical Practice Guidelines in Oncology. *J Natl Compr Canc Netw* 2024;22(2 d) doi: 10.6004/jnccn.2024.0029
5. Kopetz S, Desai J, Chan E, et al. Phase II Pilot Study of Vemurafenib in Patients With Metastatic BRAF-Mutated Colorectal Cancer. *J Clin Oncol* 2015;33(34):4032-8. doi: 10.1200/jco.2015.63.2497 [published Online First: 20151012]
6. Karapetis CS, Jonker D, Daneshmand M, et al. PIK3CA, BRAF, and PTEN status and benefit from cetuximab in the treatment of advanced colorectal cancer--results from NCIC CTG/AGITG CO.17. *Clin Cancer Res* 2014;20(3):744-53. doi: 10.1158/1078-0432.Ccr-13-0606 [published Online First: 20131111]
7. Corcoran RB, Ebi H, Turke AB, et al. EGFR-mediated re-activation of MAPK signaling contributes to insensitivity of BRAF mutant colorectal cancers to RAF inhibition with vemurafenib. *Cancer Discov* 2012;2(3):227-35. doi: 10.1158/2159-8290.Cd-11-0341 [published Online First: 20120116]
8. Prahallad A, Sun C, Huang S, et al. Unresponsiveness of colon cancer to BRAF(V600E) inhibition through feedback activation of EGFR. *Nature* 2012;483(7387):100-3. doi: 10.1038/nature10868 [published Online First: 20120126]
9. Tabernero J, Grothey A, Van Cutsem E, et al. Encorafenib Plus Cetuximab as a New Standard of Care for Previously Treated BRAF V600E-Mutant Metastatic Colorectal Cancer: Updated

Survival Results and Subgroup Analyses from the BEACON Study. *J Clin Oncol* 2021;39(4):273-84. doi: 10.1200/jco.20.02088

10. Kopetz S, Yoshino T, Van Cutsem E, et al. Encorafenib, cetuximab and chemotherapy in BRAF-mutant colorectal cancer: a randomized phase 3 trial. *Nat Med* 2025;31(3):901-08. doi: 10.1038/s41591-024-03443-3 [published Online First: 20250125]
11. Kopetz S, Guthrie KA, Morris VK, et al. Randomized Trial of Irinotecan and Cetuximab With or Without Vemurafenib in BRAF-Mutant Metastatic Colorectal Cancer (SWOG S1406). *J Clin Oncol* 2021;39(4):285-94. doi: 10.1200/jco.20.01994 [published Online First: 20201223]
12. Cornell JE. The PRISMA extension for network meta-analysis: bringing clarity and guidance to the reporting of systematic reviews incorporating network meta-analyses. *Annals of internal medicine* 2015;162(11):797-8. doi: 10.7326/m15-0930 [published Online First: 2015/06/02]
13. Tierney JF, Burdett S, Fisher DJ. Practical methods for incorporating summary time-to-event data into meta-analysis: updated guidance. *Syst Rev* 2025;14(1):84. doi: 10.1186/s13643-025-02752-z [published Online First: 20250410]
14. Tierney JF, Stewart LA, Ghersi D, et al. Practical methods for incorporating summary time-to-event data into meta-analysis. *Trials* 2007;8:16. doi: 10.1186/1745-6215-8-16 [published Online First: 20070607]
15. Sterne JAC, Savović J, Page MJ, et al. RoB 2: a revised tool for assessing risk of bias in randomised trials. *Bmj* 2019;366:l4898. doi: 10.1136/bmj.l4898 [published Online First: 20190828]
16. Slim K, Nini E, Forestier D, et al. Methodological index for non-randomized studies (minors): development and validation of a new instrument. *ANZ J Surg* 2003;73(9):712-6. doi: 10.1046/j.1445-2197.2003.02748.x
17. Coles B, Tyrer F, Hussein H, et al. Development, content validation, and reliability of the Assessment of Real-World Observational Studies (ArRoWS) critical appraisal tool. *Ann Epidemiol* 2021;55:57-63.e15. doi: 10.1016/j.annepidem.2020.09.014 [published Online First: 20201001]

18. Dias S, Welton NJ, Caldwell DM, et al. Checking consistency in mixed treatment comparison meta-analysis. *Stat Med* 2010;29(7-8):932-44. doi: 10.1002/sim.3767 [published Online First: 2010/03/10]

19. Cremolini C, Antoniotti C, Stein A, et al. Individual Patient Data Meta-Analysis of FOLFOXIRI Plus Bevacizumab Versus Doublets Plus Bevacizumab as Initial Therapy of Unresectable Metastatic Colorectal Cancer. *J Clin Oncol* 2020:Jco2001225. doi: 10.1200/jco.20.01225 [published Online First: 20200820]

20. Petrelli F, Antista M, Dottorini L, et al. First line therapy in stage IV BRAF mutated colorectal cancer. *Heliyon* 2024;10(17):e36497. doi: 10.1016/j.heliyon.2024.e36497 [published Online First: 20240822]
